# Supplementary material for: NOTCH1 intracellular domain stabilization by MDM2 plays a major role in NSCLC response to platinum
Source: EMBO Mol Med. 2026 Jan 16;18(2):514–41. doi: 10.1038/s44321-025-00354-9 (PMC12905330; doi:10.1038/s44321-025-00354-9)
Supplement: Supplementary file 2 — Source data Fig. 1 [file 44321_2025_354_MOESM2_ESM.zip › Fig1/101123 resultaTs authentif cell lines A549.pdf]

Eurofins Genomics Europe Food/Environment/White Biotech Products & Services GmbH, Anzinger Str. 7 a, D-85560 Ebersberg

eric fabbrizio
INSERM U1194, IRCM
208 rue des apothicaires
34298 Montpellier Cedex5
France

eurofins | Genomics

SOP_APG_Zelllinienauthentizität_A04_2.0

Person in charge: Dr. Torsten Brendel
Report date: 09.11.2023
Sample received on: 02.11.2023
Start / End of Analysis: 02.11.2023/09.11.2023

Analytical Report:
Cell Line Authentication Test
Order ID: 11108644027

Method:
DNA isolation was carried out from cell pellet (cell layer).
Genetic characteristics were determined by PCR-single-locus-technology.
16 independent PCR-systems D8S1179, D21S11, D7S820, CSF1PO, D3S1358, TH01, D13S317, D16S539, D2S1338, AMEL, D5S818, FGA, D19S433, vWA, TPOX and D18S51 were investigated.
(ASN-0002 core markers are colored grey, Thermo Fisher, AmpFlSTR® Identifiler® Plus PCR Amplification Kit)
In parallel, positive and negative controls were carried out yielding correct results.
Method details are given in **SOP_APG_Zelllinienauthentizität_2.0**

Result:

**Client Sample Name**	EBC1	BEAS-CLONE6	BEAS-CLONE23	EBC1 LISA	A549-GFP LUC	A549S	A549R	H460S	H460R
**Sample Code**	CL00015463	CL00015464	CL00015465	CL00015466	CL00015467	CL00015468	CL00015469	CL00015470	CL00015471
D8S1179	14,14	13,15	13,15	14,14	13,14	13,14	13,14	12,12	12,12
D21S11	29,30	28,30	28,30	29,30	29,29	29,29	29,29	30,30	30,30
D7S820	10,11	10,13	10,13	10,11	8,11	8,11	8,11	9,12	9,12
CSF1PO	10,10	9,12	9,12	10,10	10,12	10,12	10,12	11,12	11,12
D3S1358	15,15	15,17	15,17	15,15	16,16	16,16	16,16	15,18	15,18
TH01	7,7	7,9.3	7,9.3	7,7	8,9.3	8,9.3	8,9.3	9.3,9.3	9.3,9.3
D13S317	12,12	13,13	13,13	12,12	11,11	11,11	11,11	13,13	13,13
D16S539	9,9	12,12	12,12	9,9	11,12,13	11,12,13	11,12	9,9	9,9
D2S1338	16,19	22,23	22,23	16,19	24,24	24,24	24,24	17,25	17,25
D19S433	14,14	13.2,15.2*	13.2,15.2	14,14	13,13	13,13	13,13	14,14	14,14
vWA	16,17	17,18	17,18	16,17	14,14	14,14	14,14	17,17	17,17
TPOX	8,8	6,11	6,11	8,8	8,11	8,11	8,11	8,8	8,8
D18S51	12,17	*	18,18	12,17	14,17	14,17	17,17	13,15	13,15
AMEL	X,X	X,Y	X,Y	X,X	X,Y	X,Y	X,X	X,Y	X,X
D5S818	11,11	12,13	12,13	11,11	11,11	11,11	11,11	9,10,11	9,10
FGA	22,22	*	20,24	22,22	23,23	23,23	23,23	21,23	21,23

* Weak signal/No signal

The laboratory is accredited acc. to **DIN EN ISO/IEC 17025:2018**. All analyses have been carried out with greatest care and on the basis of state of the art scientific knowledge. The results refer solely to the analysed samples, as received. The duplication and publication also in parts requires a written authorization by this laboratory. Our General Terms and Conditions apply exclusively and are available under eurofinsgenomics.com

ilac-MRA DAkkS Deutsche Akkreditierungsstelle D-PL-13372-01-00

This report was created automatically and is therefore valid without a signature.
