# Supplementary material for: NOTCH1 intracellular domain stabilization by MDM2 plays a major role in NSCLC response to platinum
Source: EMBO Mol Med. 2026 Jan 16;18(2):514–41. doi: 10.1038/s44321-025-00354-9 (PMC12905330; doi:10.1038/s44321-025-00354-9)
Supplement: Supplementary file 2 — Source data Fig. 1 [file 44321_2025_354_MOESM2_ESM.zip › Fig1/20230719 resultatas authentif h358.pdf]

Eurofins Genomics Europe Applied Genomics GmbH, Anzinger Str. 7 a, D-85560 Ebersberg

eric fabbrizio
INSERM U1194, IRCM
208 rue des apothicaires
34298 Montpellier Cedex5
France

eurofins | Genomics

SOP_APG_Zelllinienauthentizität_A04_2.0

Person in charge: Dr. Torsten Brendel
Report date: 19.07.2023
Sample received on: 06.07.2023
Start / End of Analysis: 13.07.2023/19.07.2023

Analytical Report:
Cell Line Authentication Test
Order ID: 11108498713

Method:
DNA isolation was carried out from cell pellet (cell layer).
Genetic characteristics were determined by PCR-single-locus-technology.
16 independent PCR-systems D8S1179, D21S11, D7S820, CSF1PO, D3S1358, TH01, D13S317, D16S539, D2S1338, AMEL, D5S818, FGA, D19S433, vWA, TPOX and D18S51 were investigated.
(ASN-0002 core markers are colored grey, Thermo Fisher, AmpFlSTR® Identifiler® Plus PCR Amplification Kit)
In parallel, positive and negative controls were carried out yielding correct results.
Method details are given in **SOP_APG_Zelllinienauthentizität_2.0**

Result:

**Client Sample Name**	H358R-2023	H358R-2018	H358R-2017	H358S	H358R-MICE
**Sample Code**	CL00014226	CL00014227	CL00014223	CL00014225	CL00014224
D8S1179	12,12	12,12	12,12	13,14	12,12
D21S11	30,30	30,30	30,30	28,30	30,30
D7S820	9,12	9,12	*	10,11	9,12
CSF1PO	11,13	11,12,13	*	11,12	11,12
D3S1358	15,18	15,18	15,18	14,18	15,18
TH01	9.3,9.3	9.3,9.3	9.3,9.3	6,6	9.3,9.3
D13S317	13,13	13,13	13,13	8,12	13,13
D16S539	9,9	9,9	9,9	12,13	9,9
D2S1338	17,25	17,25	*	17,23	17,25
D19S433	14,15	14,14	14,14	13,14	14,14
vWA	17,17	17,17	17,17	17,17	17,17
TPOX	8,8	8,8	8,8	8,9	8,8
D18S51	13,15	13,15	*	14,15	13,13
AMEL	X,X	X,X	X,X	X,Y	X,X
D5S818	9,10	9,10	9,10	10,12	9,10
FGA	21,23	21,23	21,23	20,21	21,23

* No signal

The laboratory is accredited acc. to **DIN EN ISO/IEC 17025:2018**. All analyses have been carried out with greatest care and on the basis of state of the art scientific knowledge. The results refer solely to the analysed samples, as received. The duplication and publication also in parts requires a written authorization by this laboratory. Our General Terms and Conditions apply exclusively and are available under eurofinsgenomics.com

ilac-MRA DAkkS Deutsche Akkreditierungsstelle D-PL-13372-01-00

This report was created automatically and is therefore valid without a signature.

Eurofins Genomics Europe Applied Genomics GmbH
Anzinger Straße 7 a
85560 Ebersberg
Germany

Tel.: +49 7531 816068
Email: support-eu@genomics.eurofinseu.com
Web: www.eurofinsgenomics.com

Managing Director: Dr. Michael Hadem
Authorized Officer: Matthias Dittmer
Register Court Munich HRB 207710
VAT ID: DE815473648

HypoVereinsbank
IBAN: DE23 2073 0017 7000 0006 50
SWIFT: HYVEDEMME17
