# Supplementary figures and images for: NOTCH1 intracellular domain stabilization by MDM2 plays a major role in NSCLC response to platinum (part 1 of 3)
Source: EMBO Mol Med. 2026 Jan 16;18(2):514–41. doi: 10.1038/s44321-025-00354-9 (PMC12905330; doi:10.1038/s44321-025-00354-9)

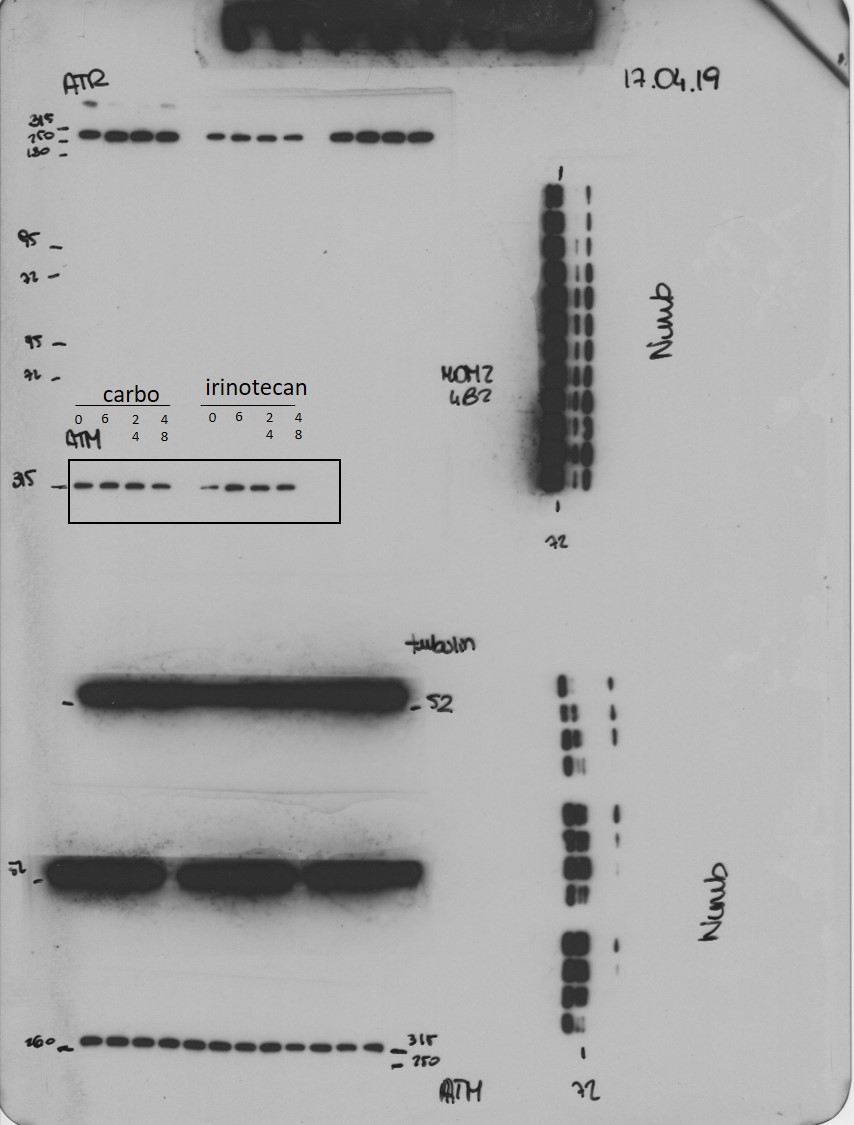

Supplement: Supplementary file 2 — Source data Fig. 1 [file 44321_2025_354_MOESM2_ESM.zip › Fig1/Fig 1A/replicate carbo, irinotecan/western blot ATM replicate.jpg]

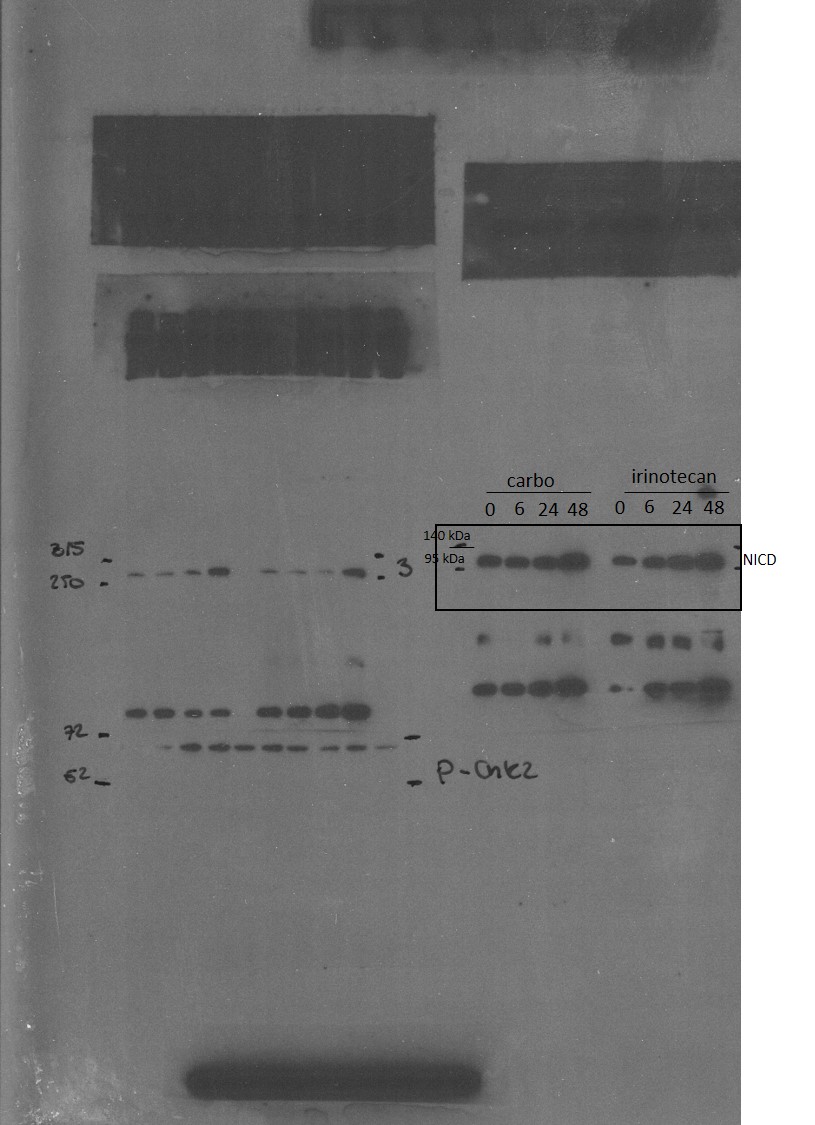

Supplement: Supplementary file 2 — Source data Fig. 1 [file 44321_2025_354_MOESM2_ESM.zip › Fig1/Fig 1A/replicate carbo, irinotecan/western blot nicd replicate.jpg]

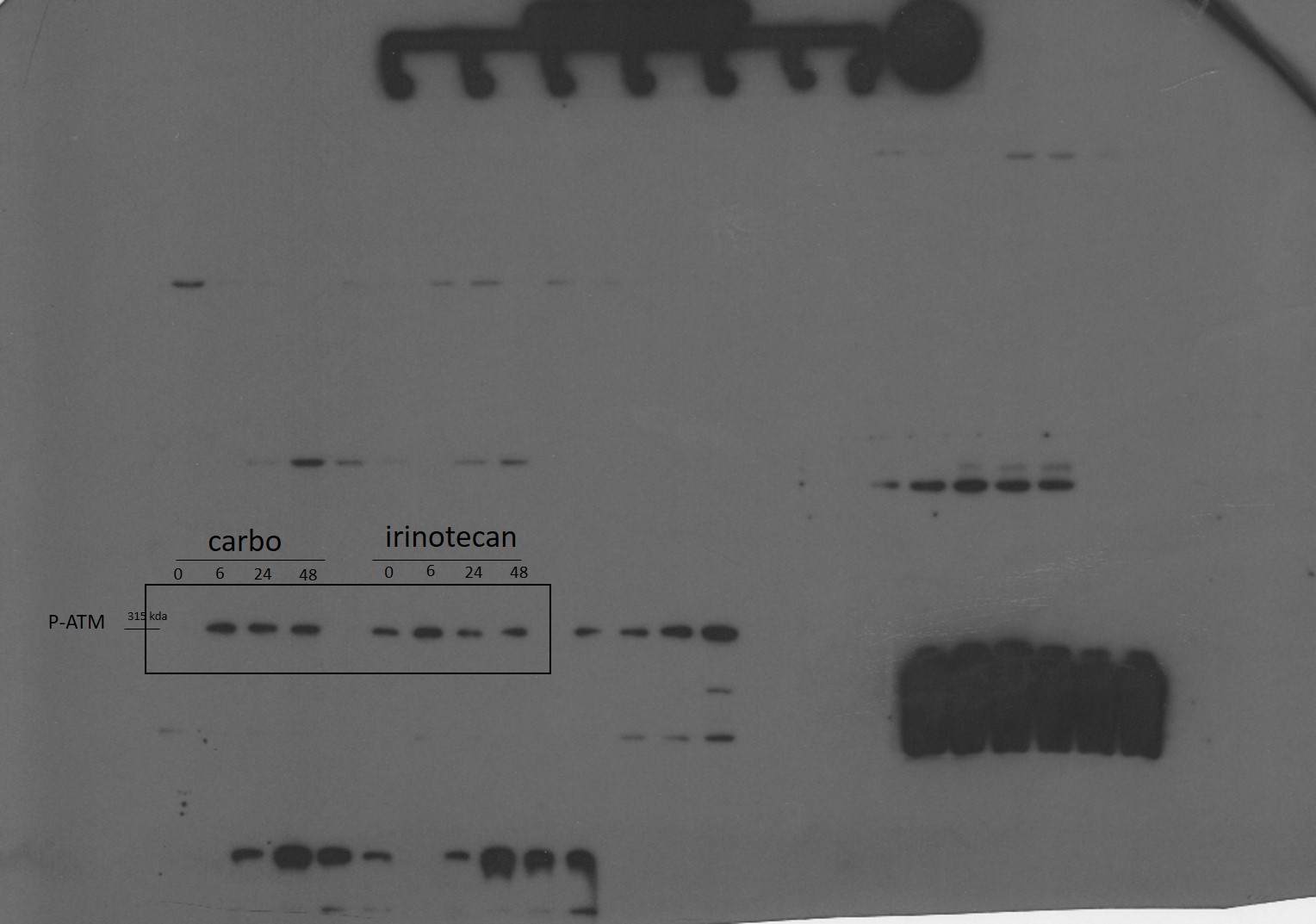

Supplement: Supplementary file 2 — Source data Fig. 1 [file 44321_2025_354_MOESM2_ESM.zip › Fig1/Fig 1A/replicate carbo, irinotecan/western blot p-ATM replicate.jpg]

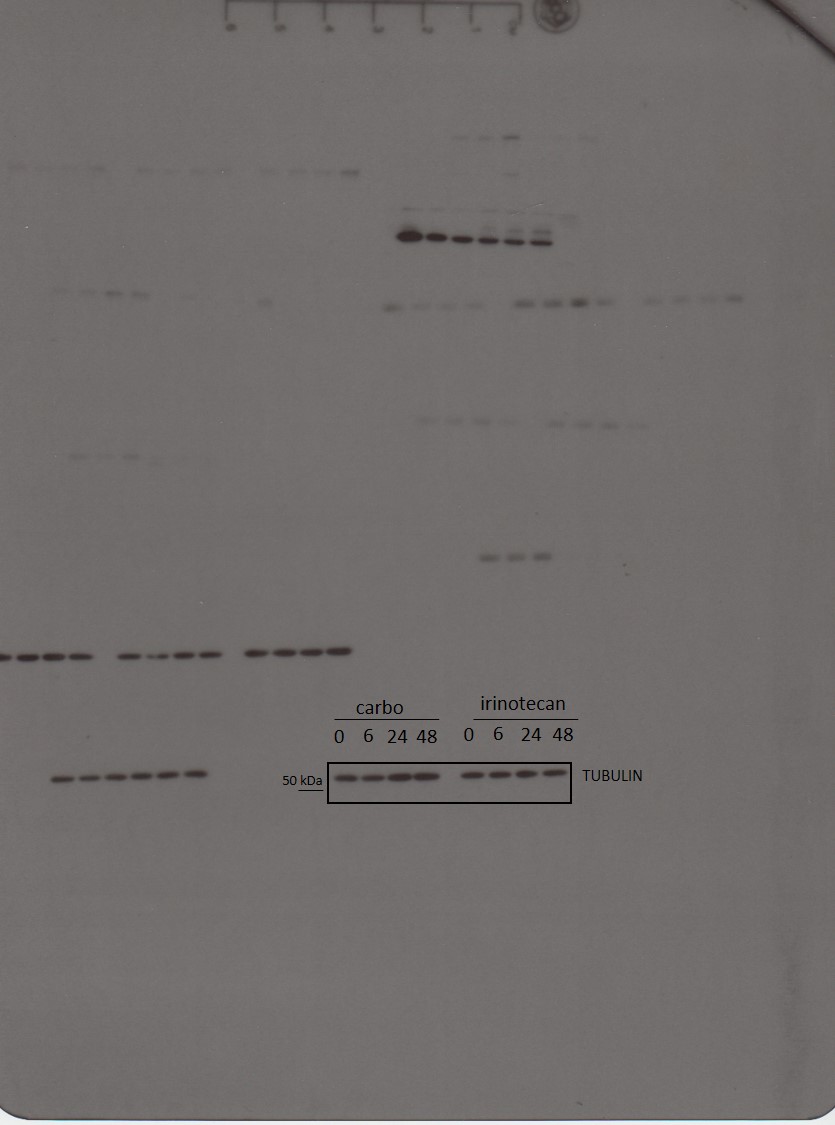

Supplement: Supplementary file 2 — Source data Fig. 1 [file 44321_2025_354_MOESM2_ESM.zip › Fig1/Fig 1A/replicate carbo, irinotecan/western blot tubulin replicate.jpg]

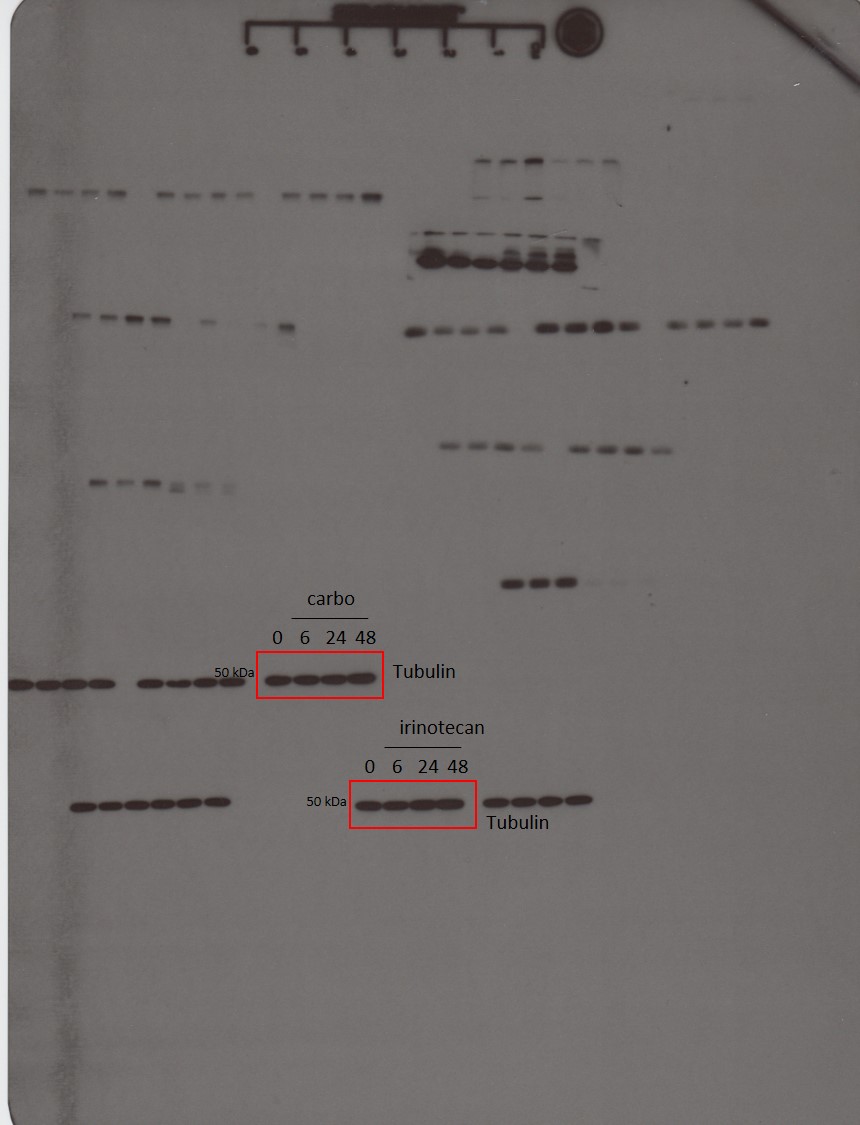

Supplement: Supplementary file 2 — Source data Fig. 1 [file 44321_2025_354_MOESM2_ESM.zip › Fig1/Fig 1A/tubulin carbo, irinotecan, wb#2.jpg]

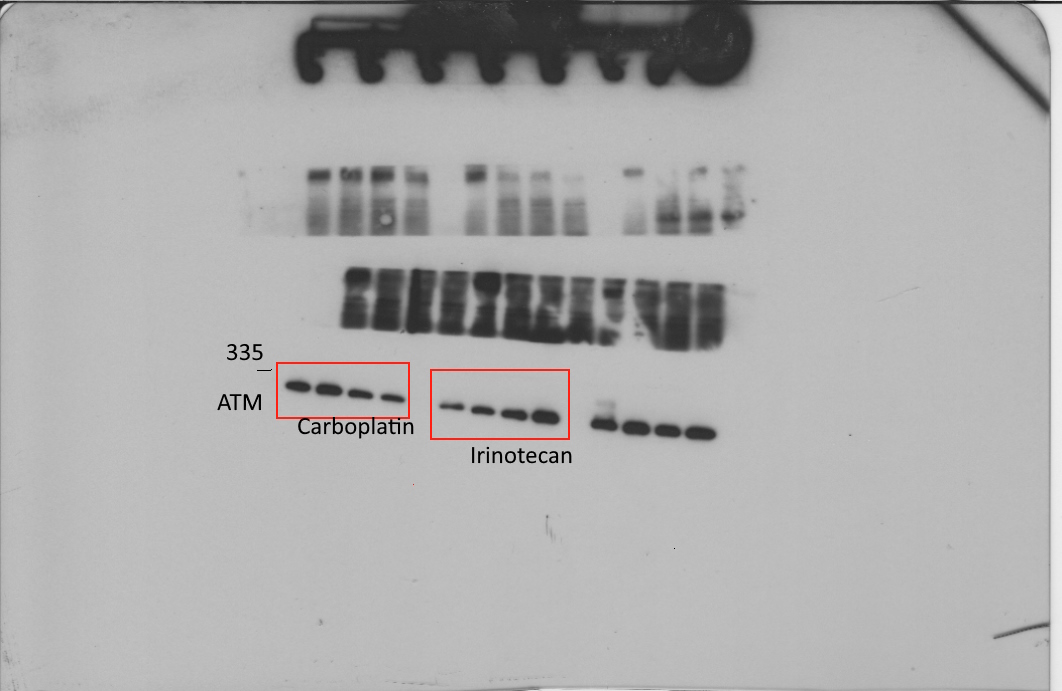

Supplement: Supplementary file 2 — Source data Fig. 1 [file 44321_2025_354_MOESM2_ESM.zip › Fig1/Fig 1A/western ATM, carboplatin, irinotecan#1.jpg]

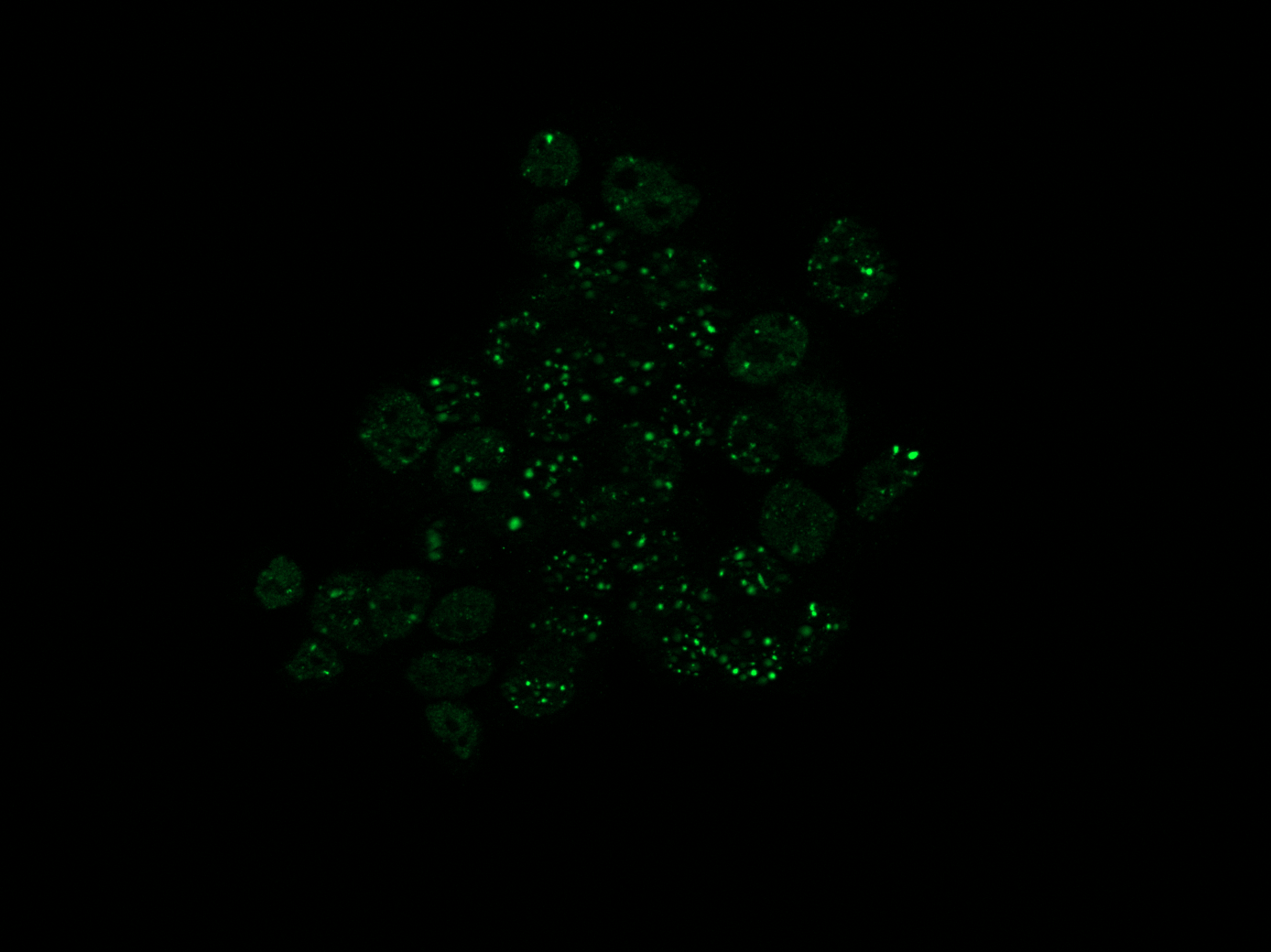

Supplement: Supplementary file 2 — Source data Fig. 1 [file 44321_2025_354_MOESM2_ESM.zip › Fig1/Fig 1B/0/IP-ApoTome-13_53BP1.tif]

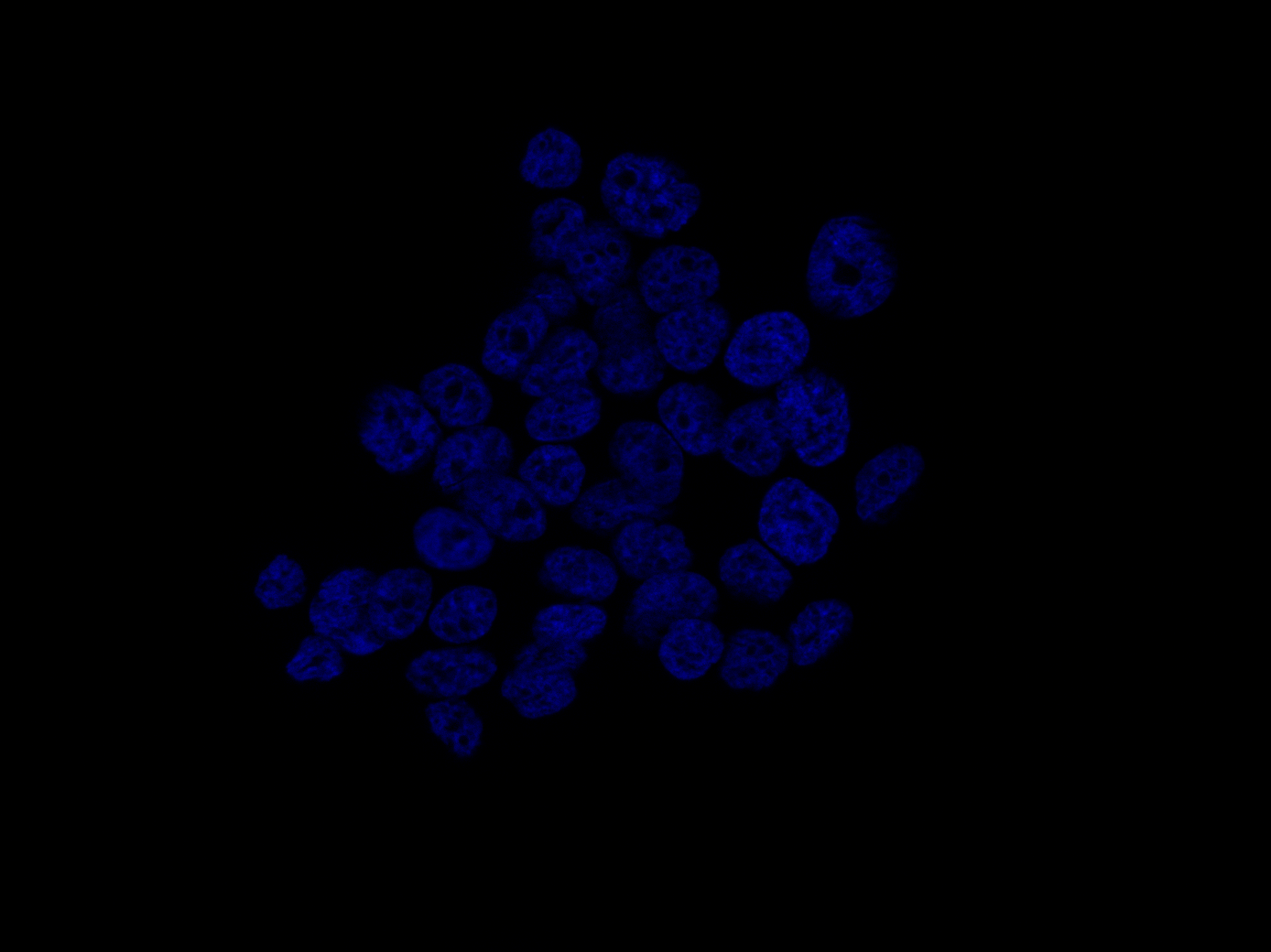

Supplement: Supplementary file 2 — Source data Fig. 1 [file 44321_2025_354_MOESM2_ESM.zip › Fig1/Fig 1B/0/IP-ApoTome-13_DAPI.tif]

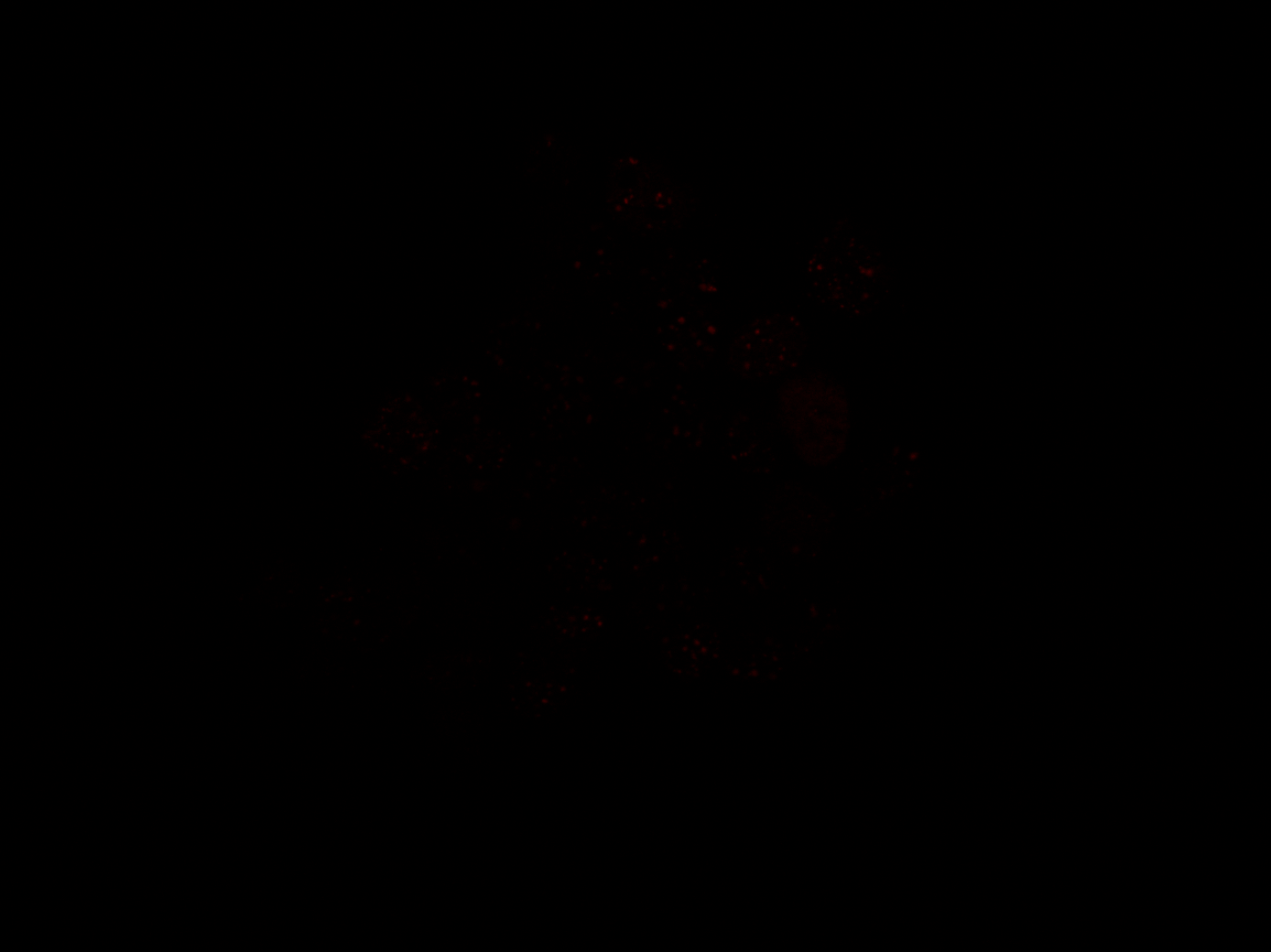

Supplement: Supplementary file 2 — Source data Fig. 1 [file 44321_2025_354_MOESM2_ESM.zip › Fig1/Fig 1B/0/IP-ApoTome-13_H2AX.tif]

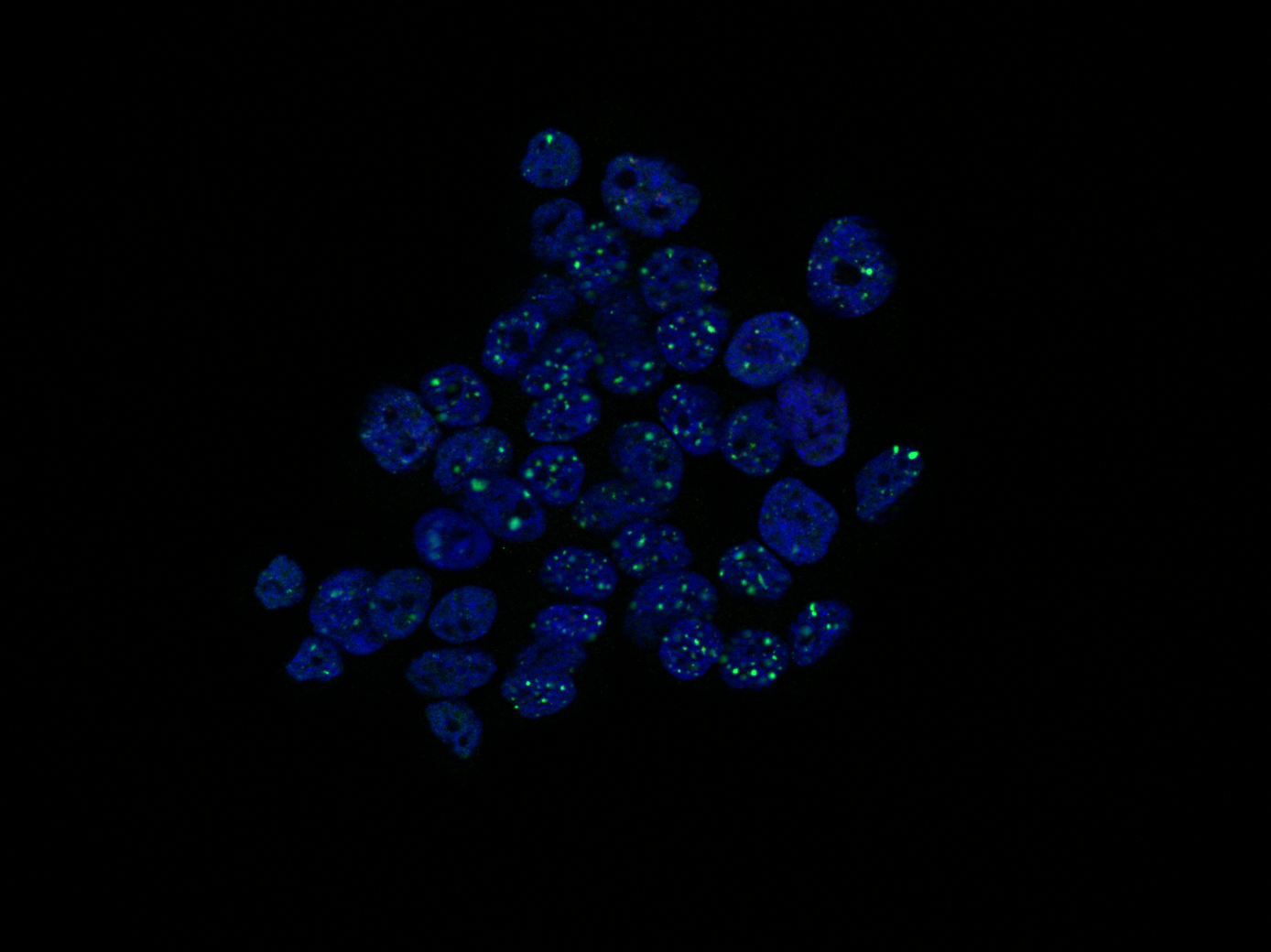

Supplement: Supplementary file 2 — Source data Fig. 1 [file 44321_2025_354_MOESM2_ESM.zip › Fig1/Fig 1B/0/IP-ApoTome-13_merge.tif]

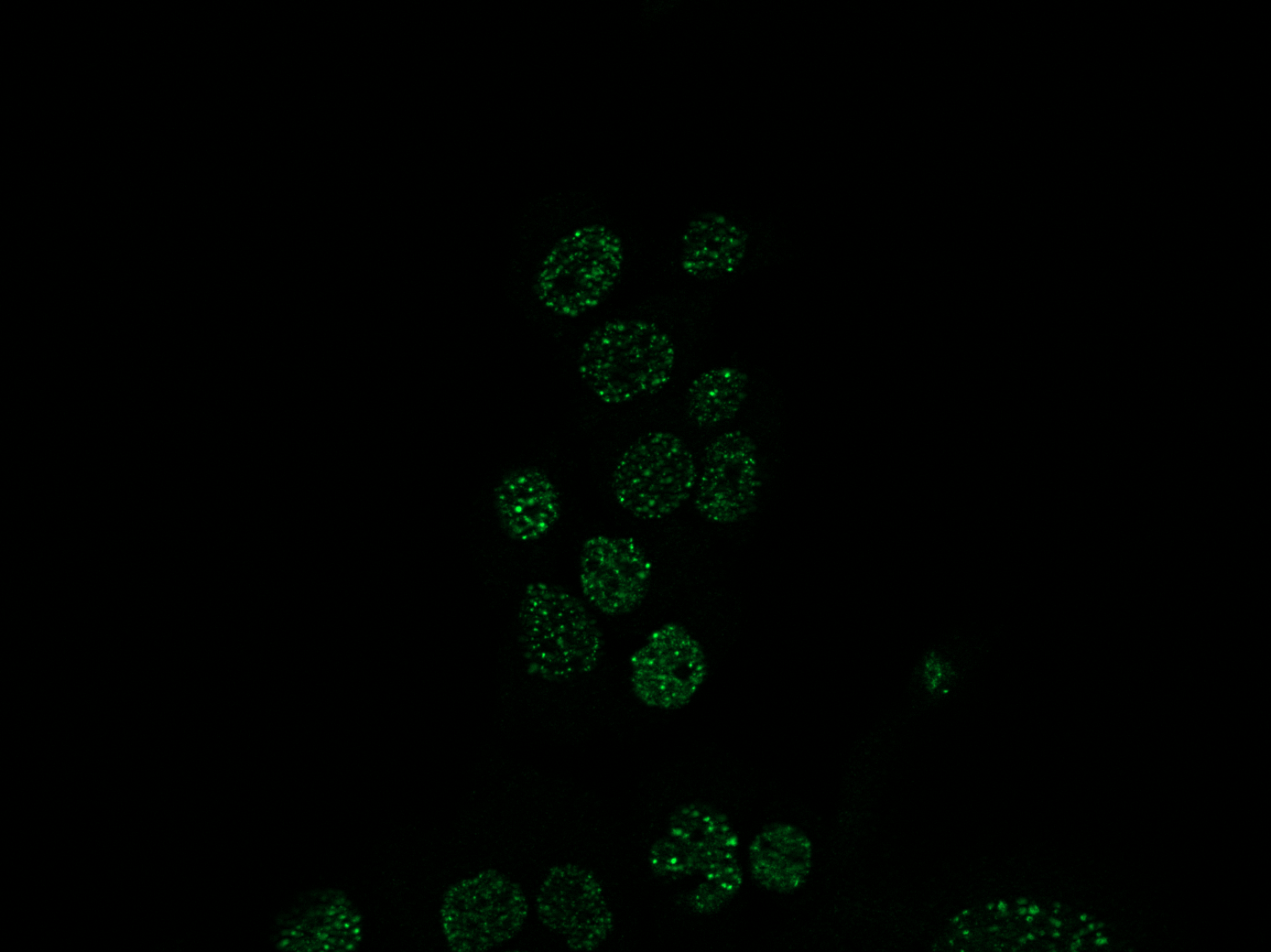

Supplement: Supplementary file 2 — Source data Fig. 1 [file 44321_2025_354_MOESM2_ESM.zip › Fig1/Fig 1B/24/IP-ApoTome-11_53bp1.tif]

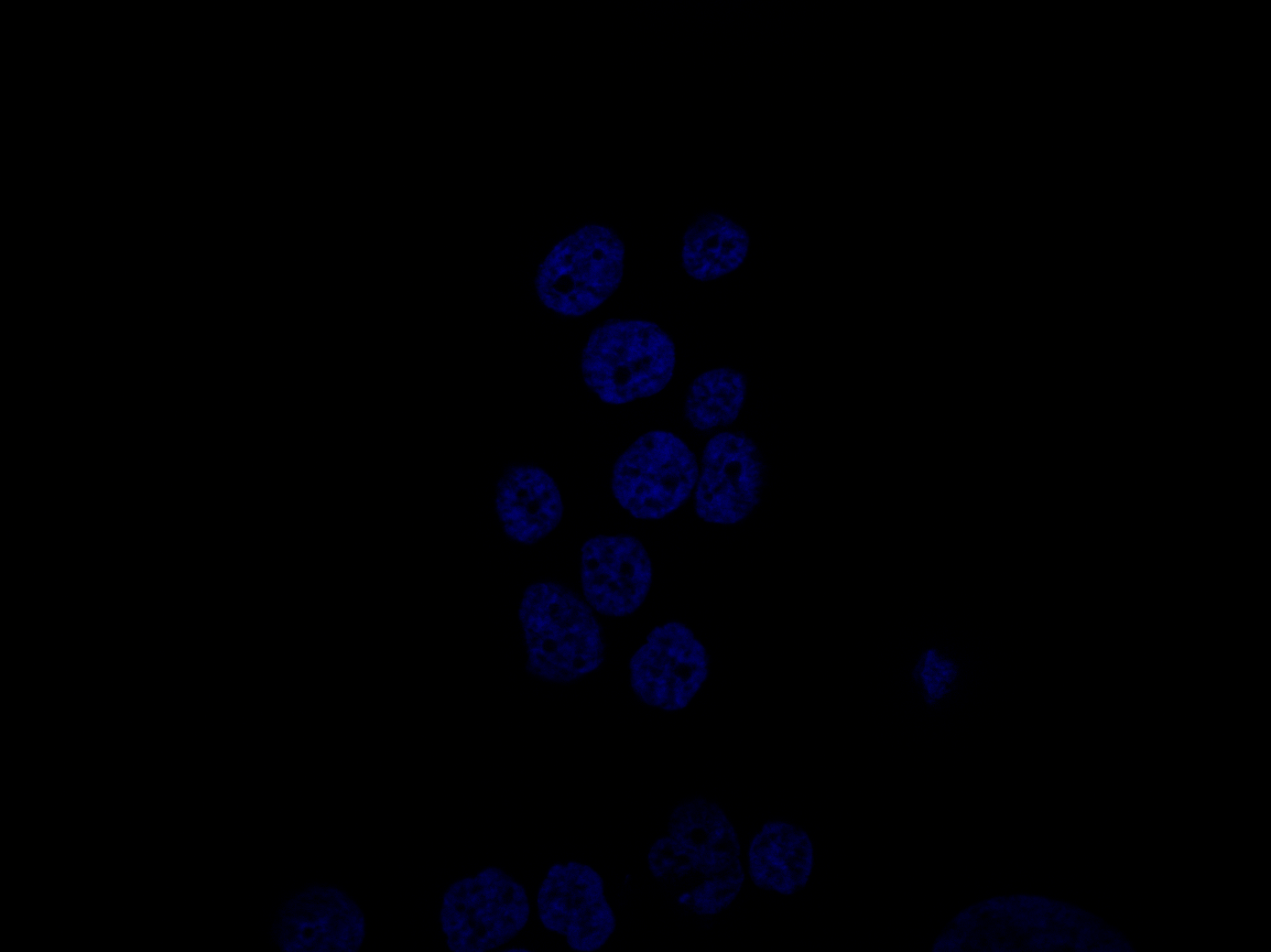

Supplement: Supplementary file 2 — Source data Fig. 1 [file 44321_2025_354_MOESM2_ESM.zip › Fig1/Fig 1B/24/IP-ApoTome-11_DAPI.tif]

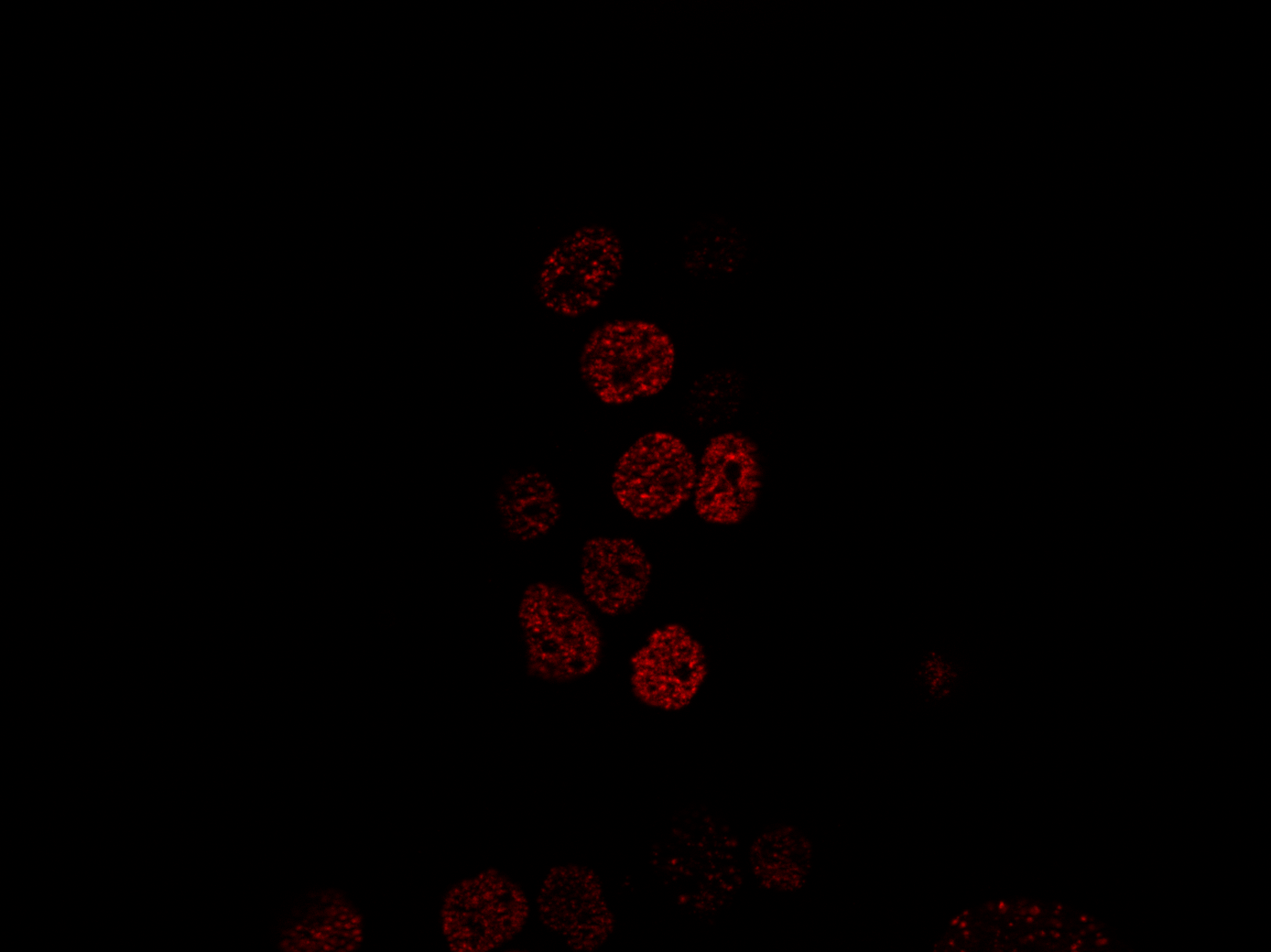

Supplement: Supplementary file 2 — Source data Fig. 1 [file 44321_2025_354_MOESM2_ESM.zip › Fig1/Fig 1B/24/IP-ApoTome-11_h2ax.tif]

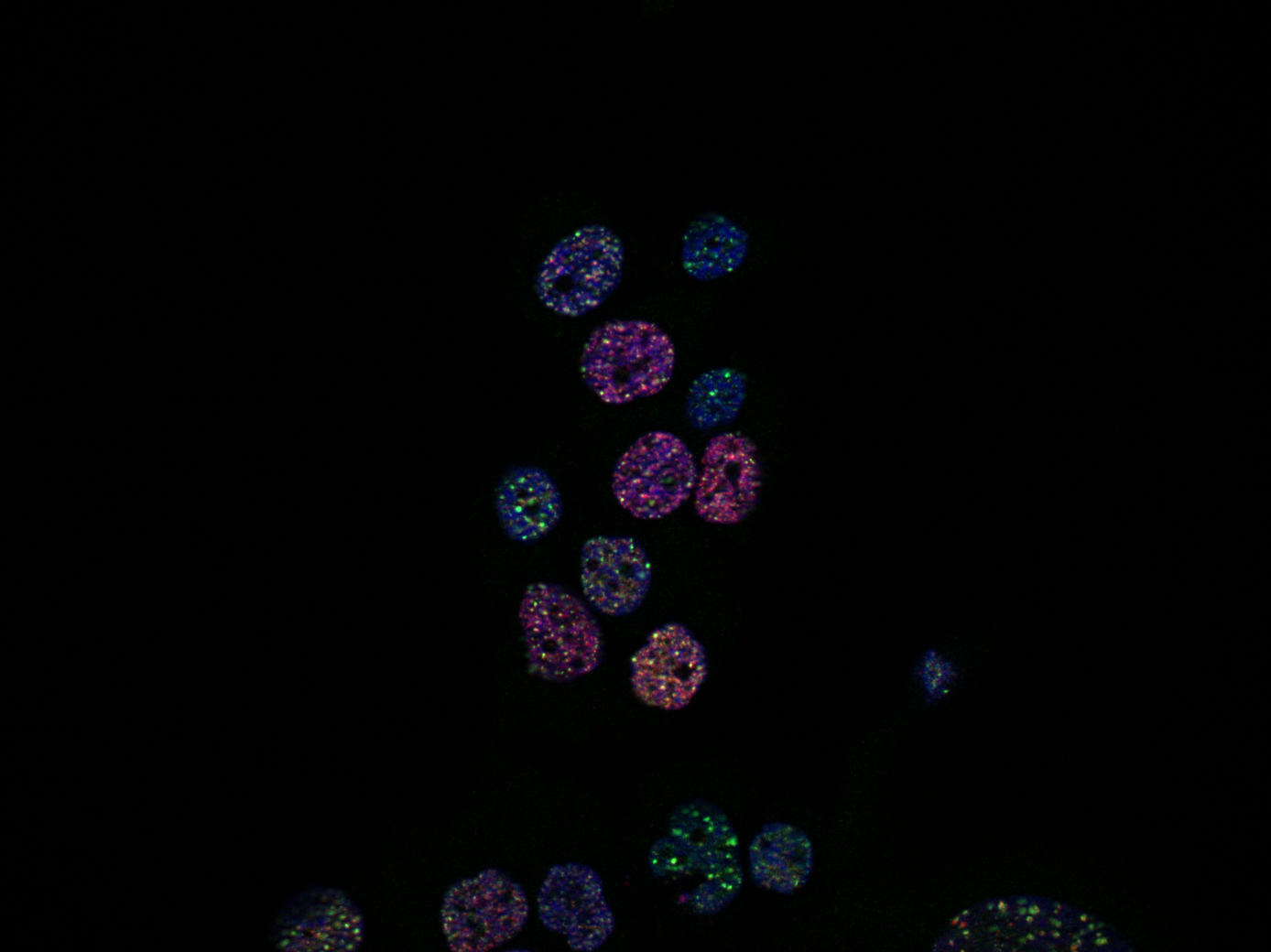

Supplement: Supplementary file 2 — Source data Fig. 1 [file 44321_2025_354_MOESM2_ESM.zip › Fig1/Fig 1B/24/IP-ApoTome-11_merge.tif]

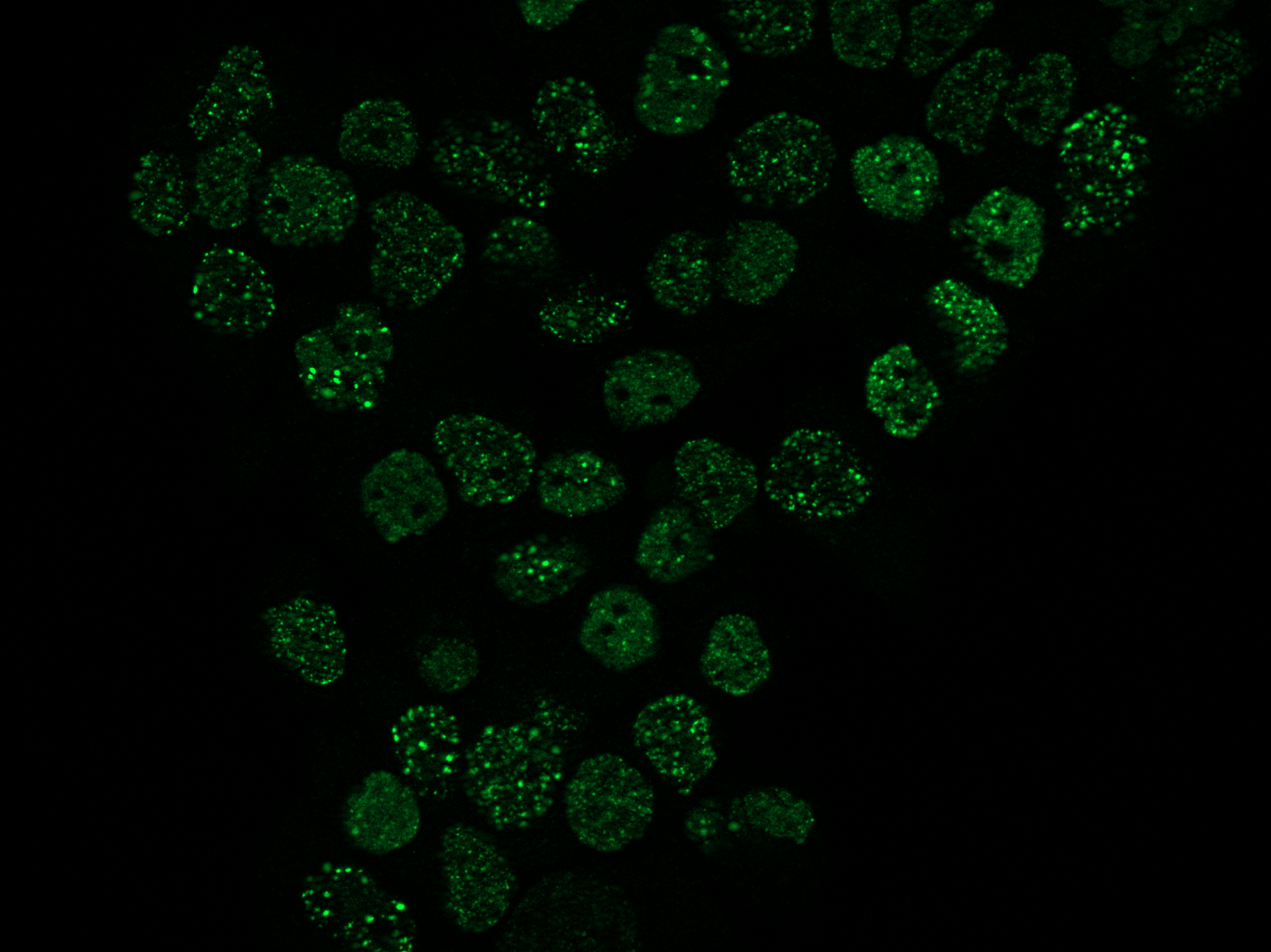

Supplement: Supplementary file 2 — Source data Fig. 1 [file 44321_2025_354_MOESM2_ESM.zip › Fig1/Fig 1B/48/IP-ApoTome-05_53bp1.tif]

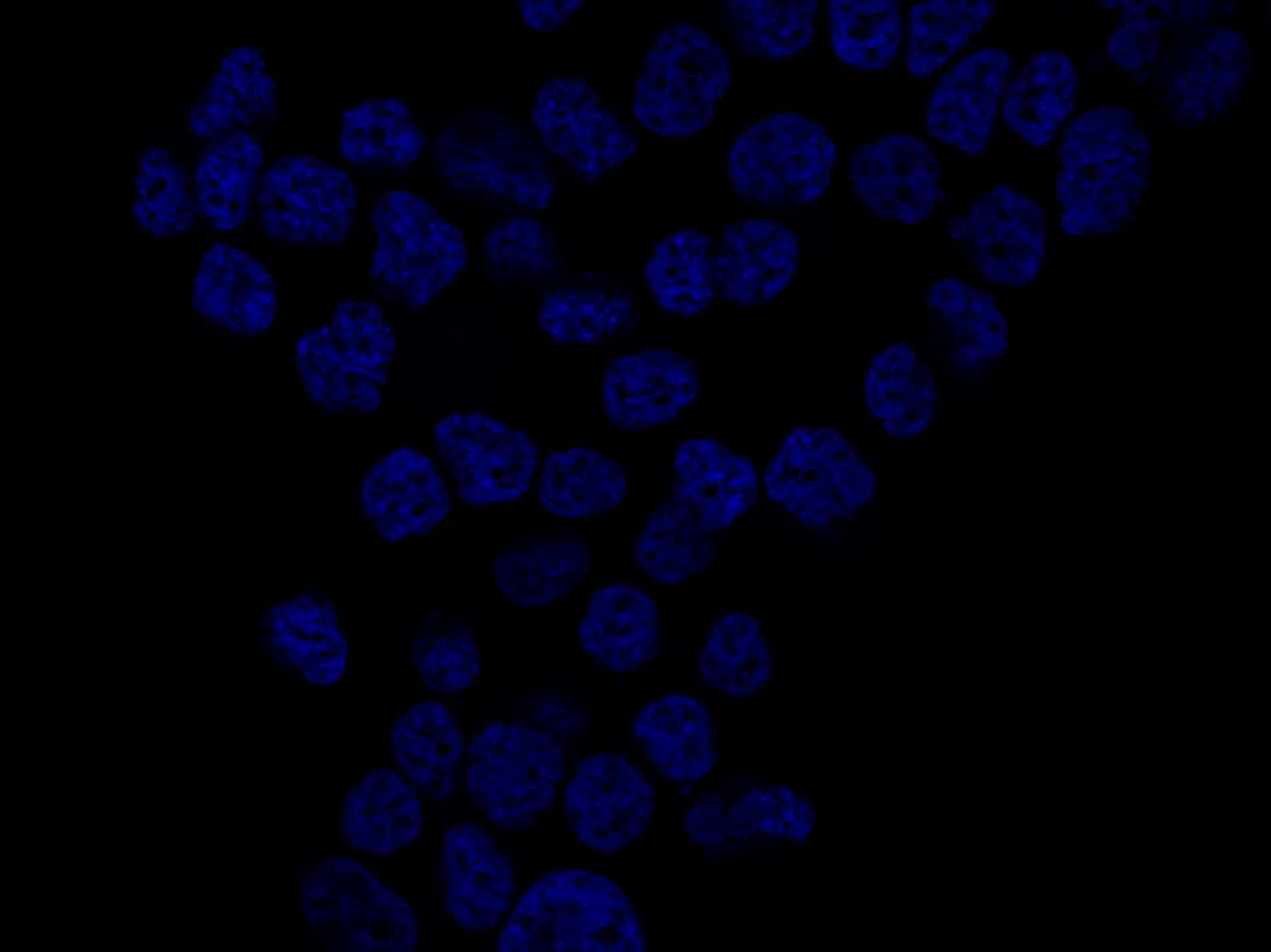

Supplement: Supplementary file 2 — Source data Fig. 1 [file 44321_2025_354_MOESM2_ESM.zip › Fig1/Fig 1B/48/IP-ApoTome-05_DAPI.tif]

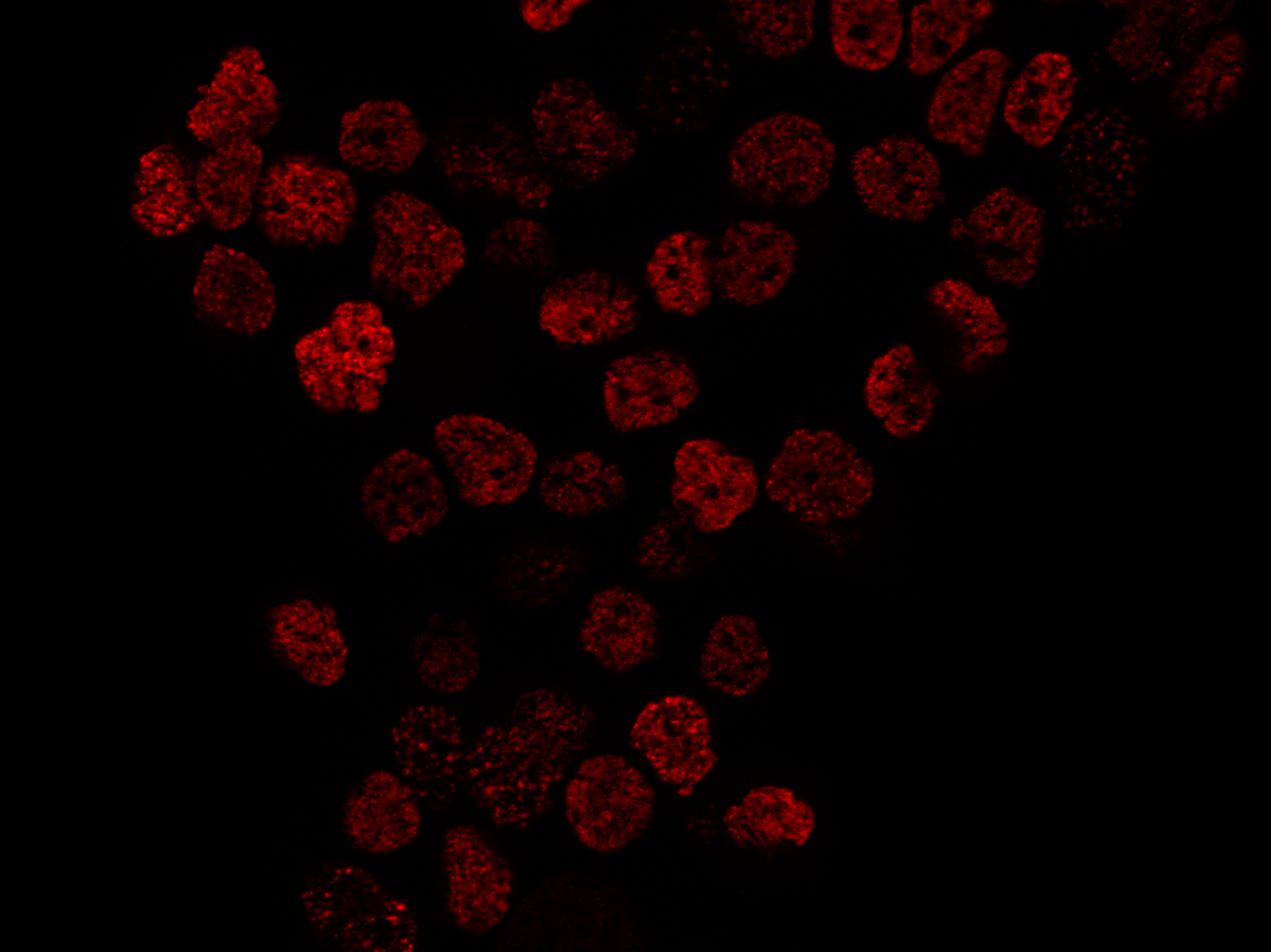

Supplement: Supplementary file 2 — Source data Fig. 1 [file 44321_2025_354_MOESM2_ESM.zip › Fig1/Fig 1B/48/IP-ApoTome-05_h2ax.tif]

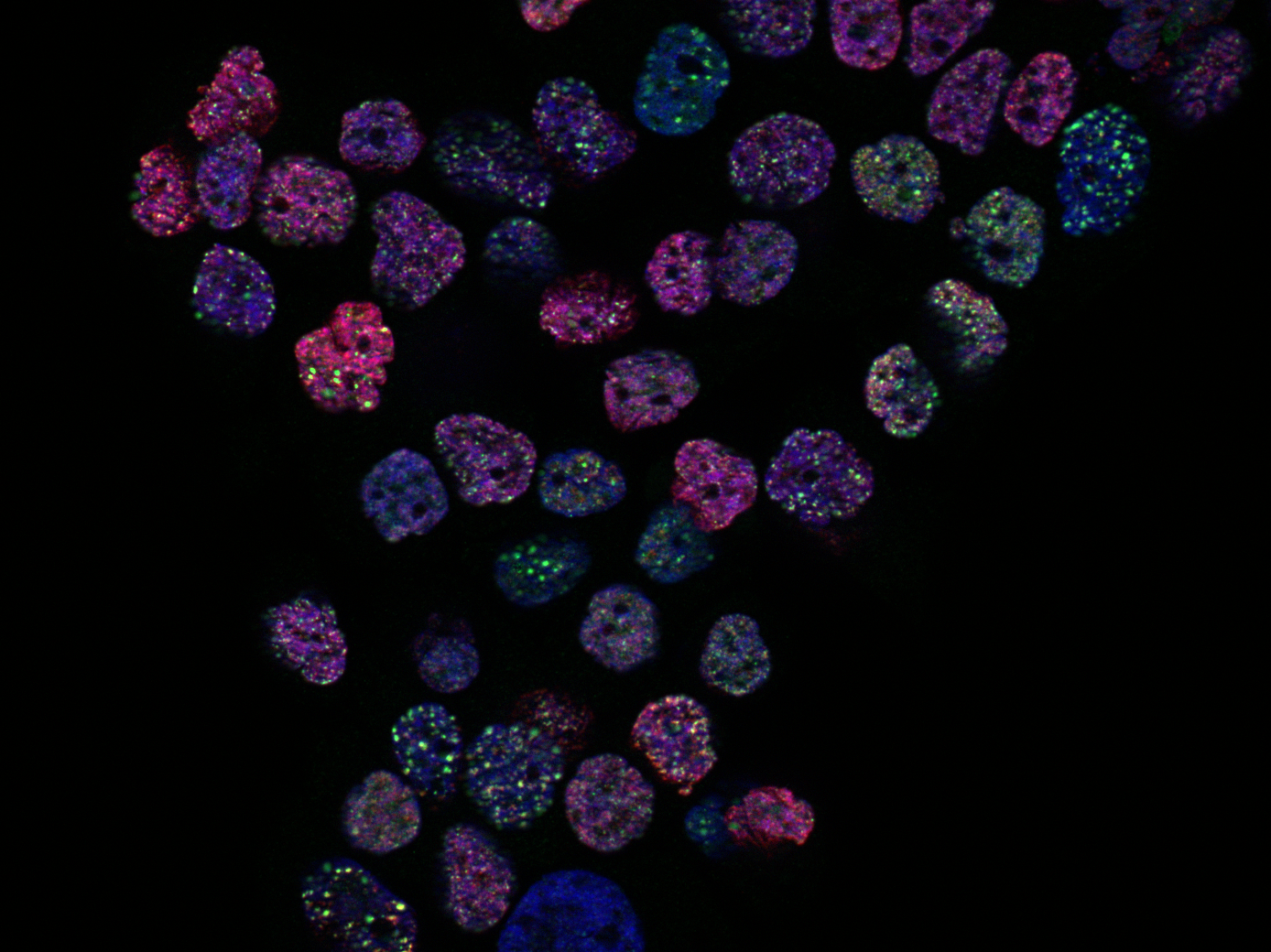

Supplement: Supplementary file 2 — Source data Fig. 1 [file 44321_2025_354_MOESM2_ESM.zip › Fig1/Fig 1B/48/IP-ApoTome-05_merge.tif]

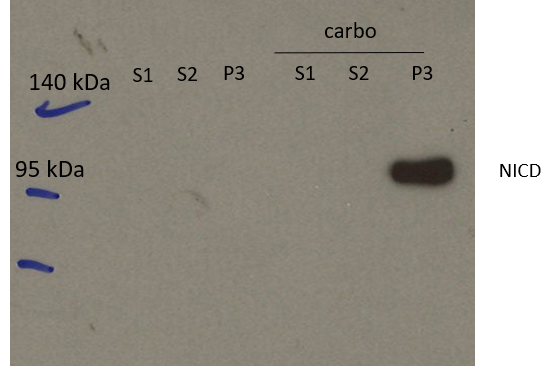

Supplement: Supplementary file 2 — Source data Fig. 1 [file 44321_2025_354_MOESM2_ESM.zip › Fig1/Fig 1C/Fig 1C Replicate/western blot NICD N2.png]

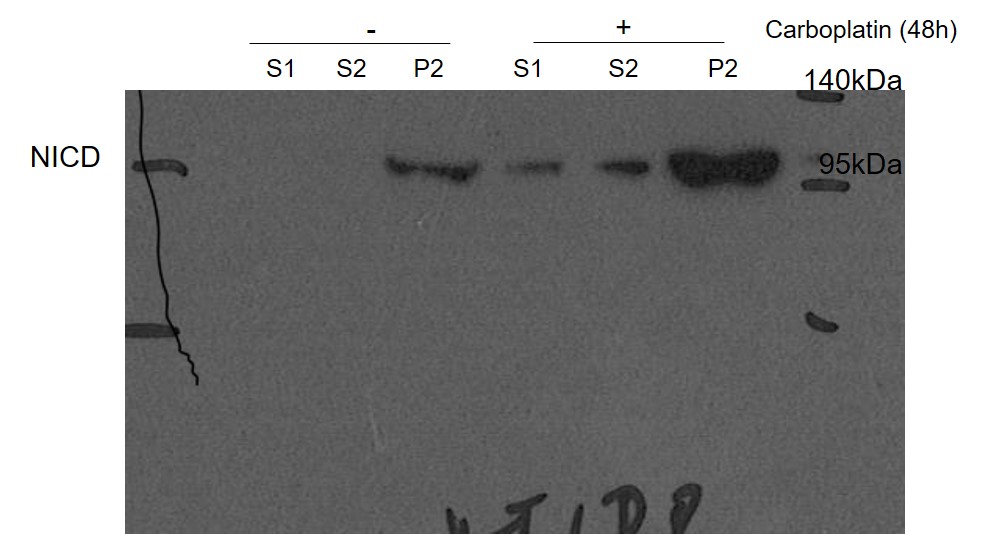

Supplement: Supplementary file 2 — Source data Fig. 1 [file 44321_2025_354_MOESM2_ESM.zip › Fig1/Fig 1C/Fig 1C Replicate/western blot NICD N3.jpg]

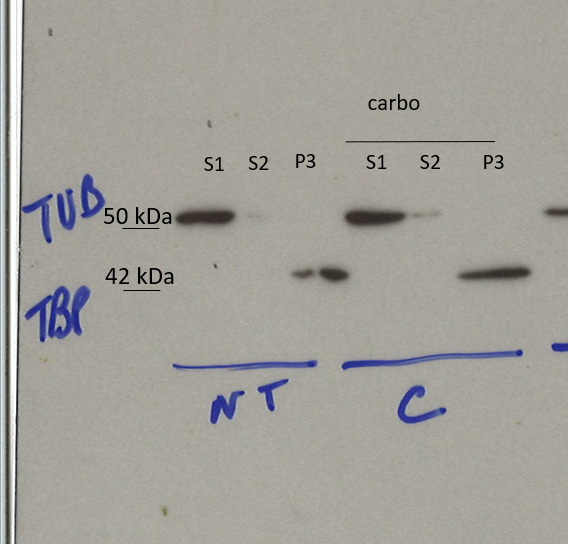

Supplement: Supplementary file 2 — Source data Fig. 1 [file 44321_2025_354_MOESM2_ESM.zip › Fig1/Fig 1C/Fig 1C Replicate/western blot tubulin, TBP N2.png]

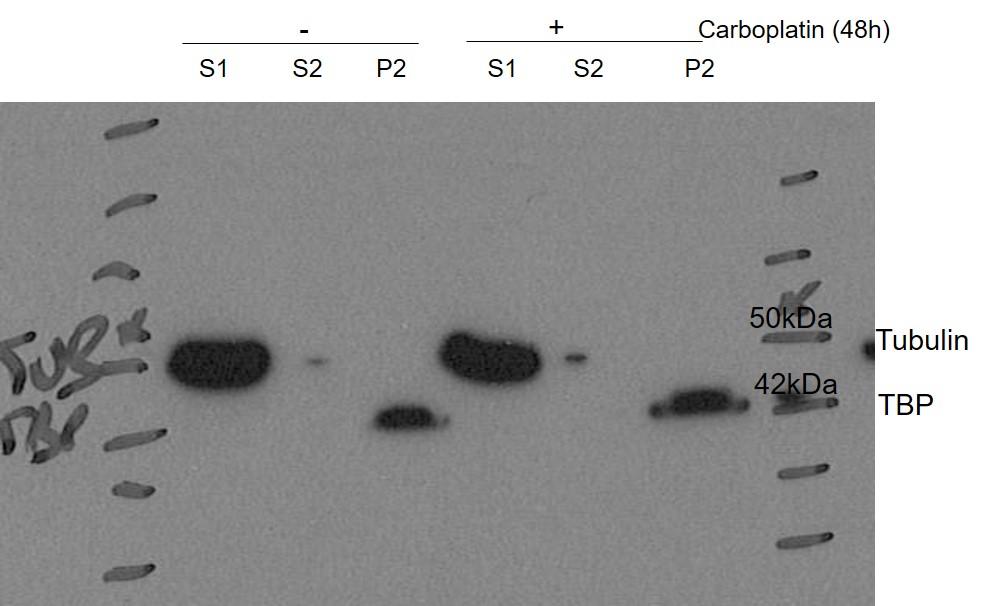

Supplement: Supplementary file 2 — Source data Fig. 1 [file 44321_2025_354_MOESM2_ESM.zip › Fig1/Fig 1C/Fig 1C Replicate/western blot tubulin, TBP N3.jpg]

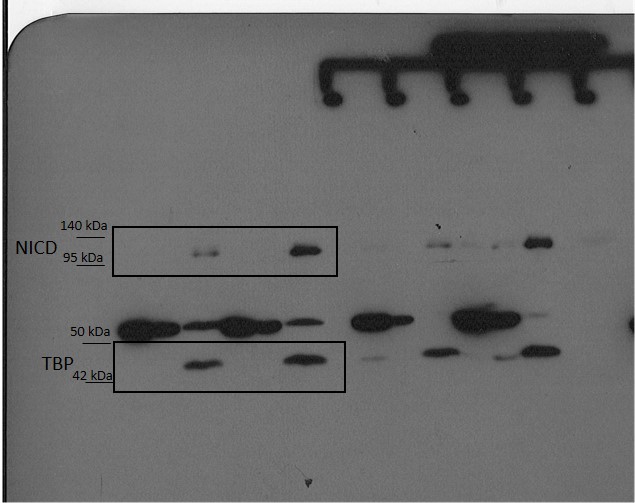

Supplement: Supplementary file 2 — Source data Fig. 1 [file 44321_2025_354_MOESM2_ESM.zip › Fig1/Fig 1C/westrn blot nicd, tbp.jpg]

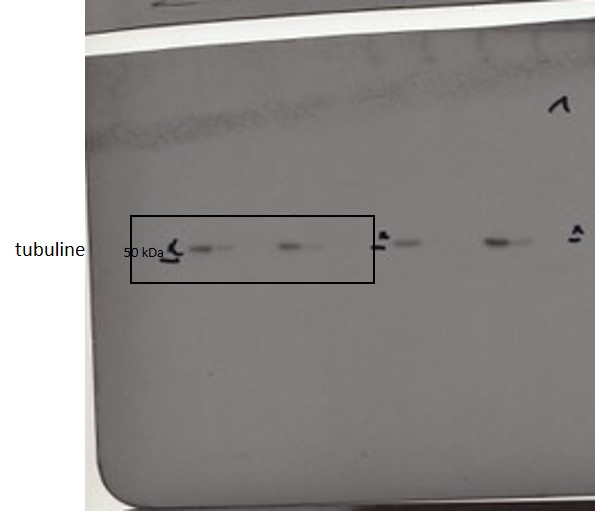

Supplement: Supplementary file 2 — Source data Fig. 1 [file 44321_2025_354_MOESM2_ESM.zip › Fig1/Fig 1C/westrn blot tubulin.jpg]

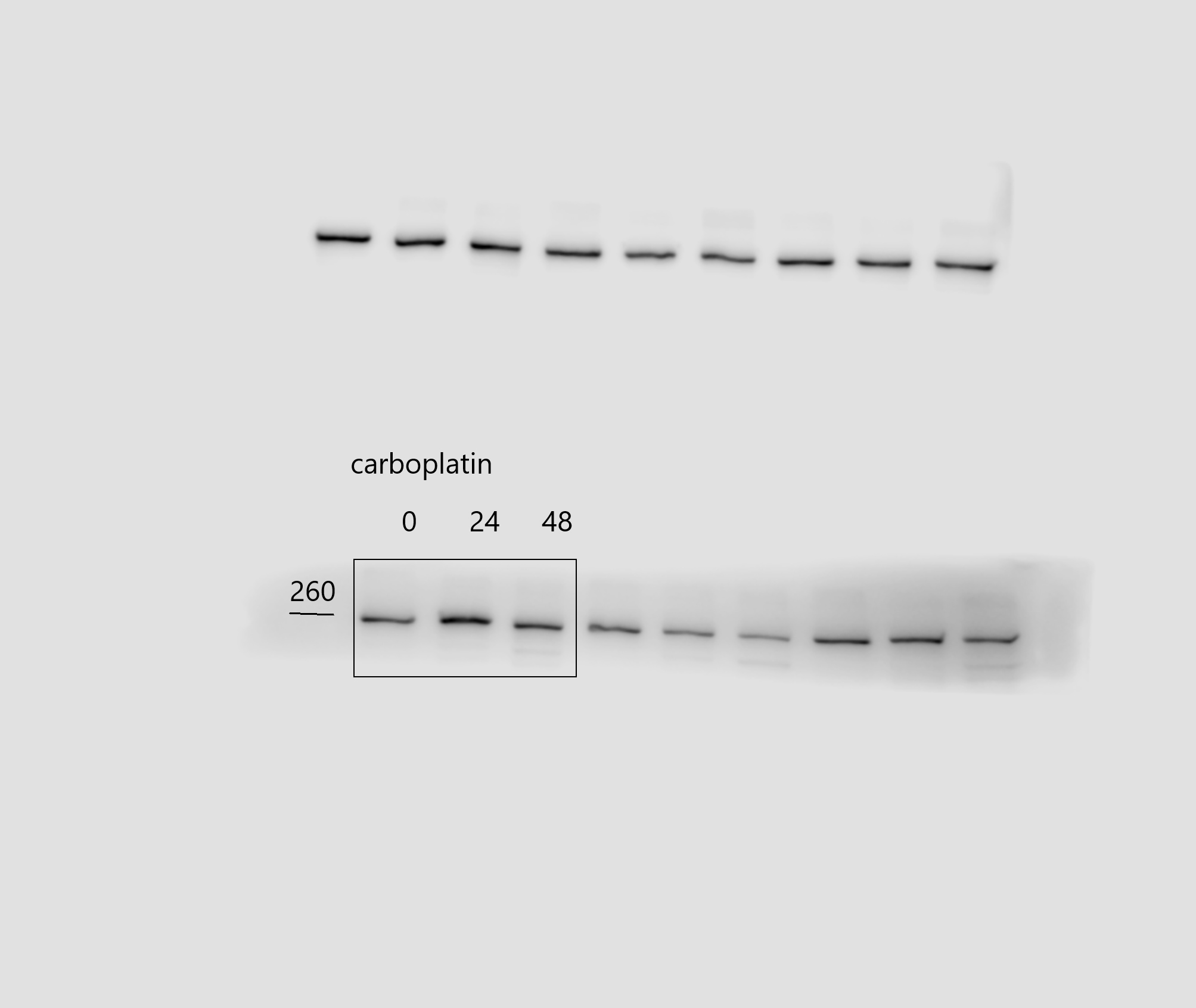

Supplement: Supplementary file 2 — Source data Fig. 1 [file 44321_2025_354_MOESM2_ESM.zip › Fig1/Fig 1D/ATM, wb4.jpg]

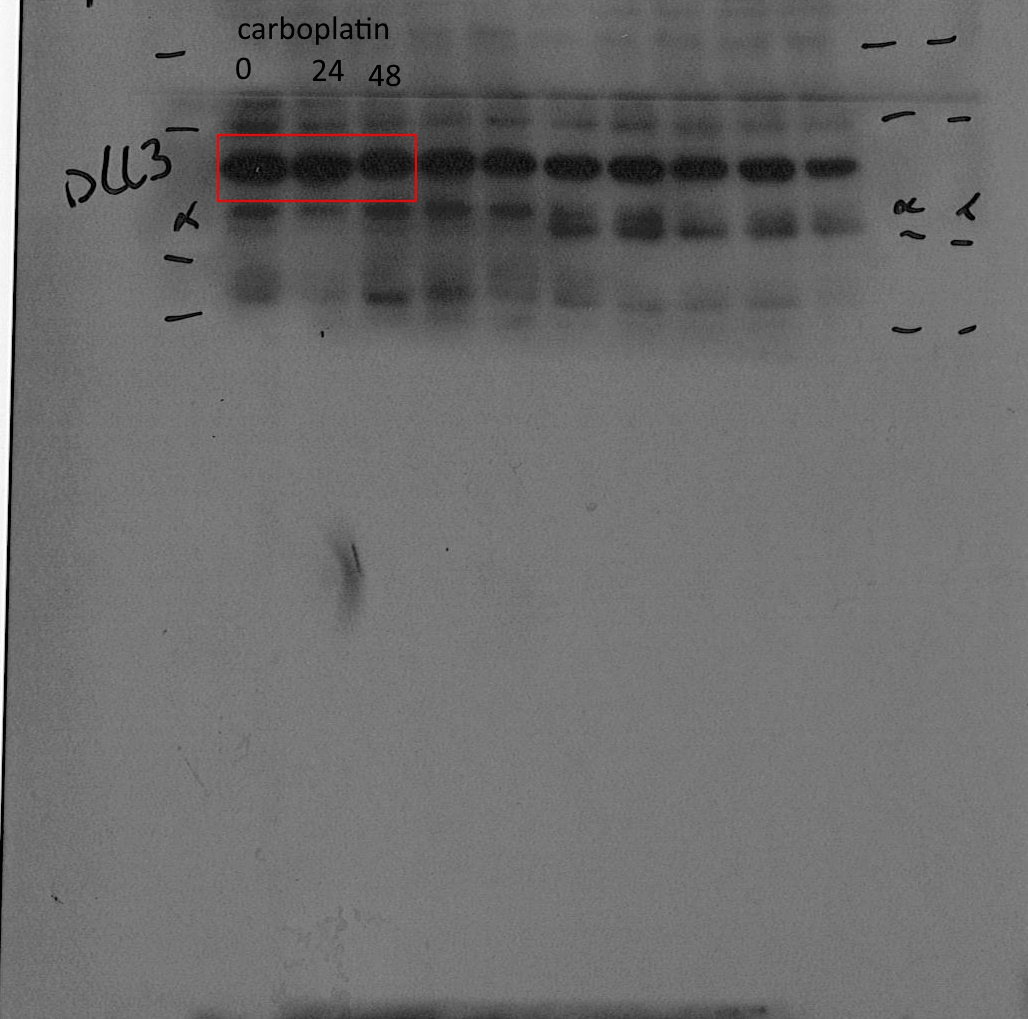

Supplement: Supplementary file 2 — Source data Fig. 1 [file 44321_2025_354_MOESM2_ESM.zip › Fig1/Fig 1D/dll3, wb3.jpg]

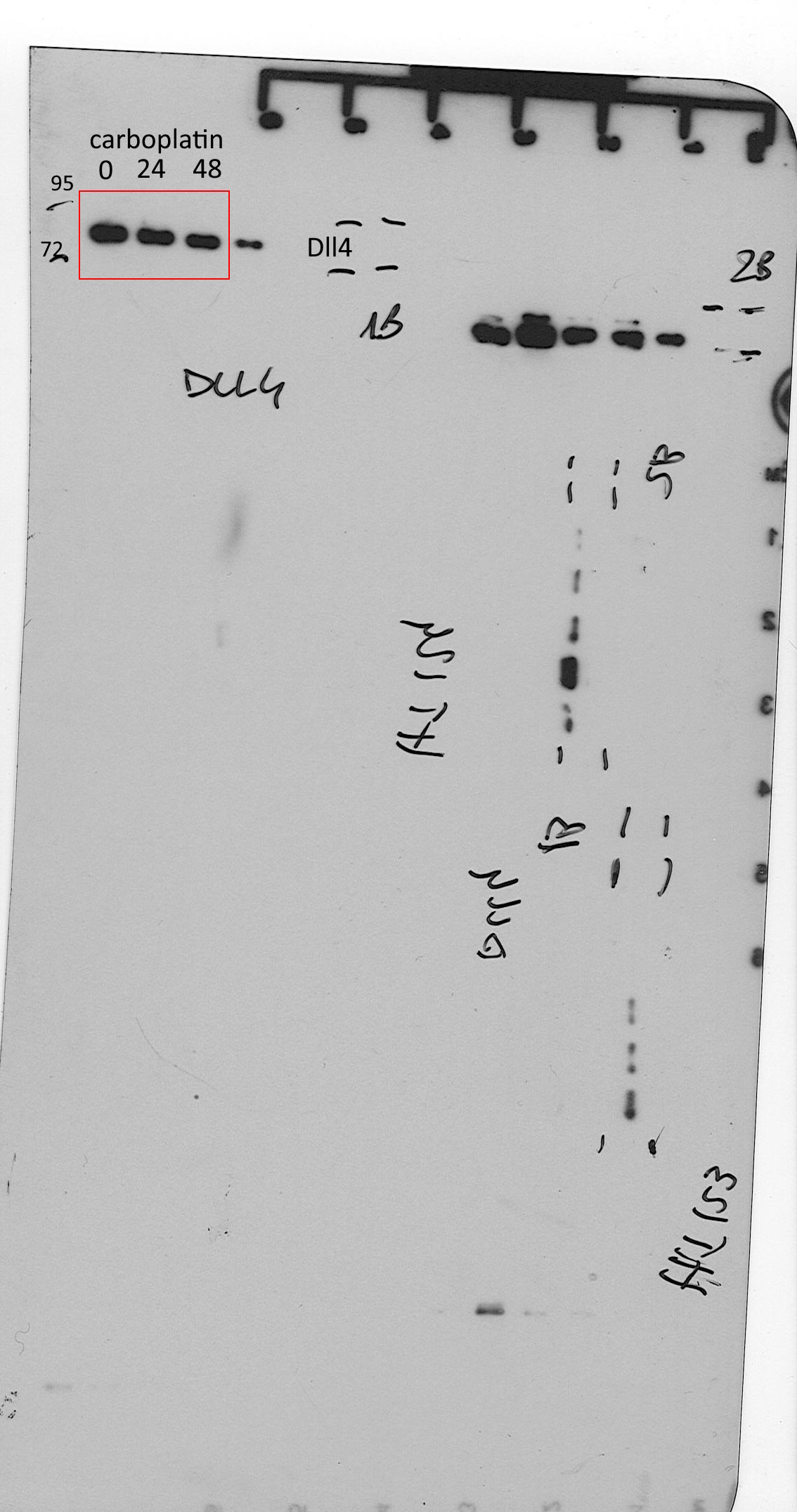

Supplement: Supplementary file 2 — Source data Fig. 1 [file 44321_2025_354_MOESM2_ESM.zip › Fig1/Fig 1D/dll4, wb1.jpg]

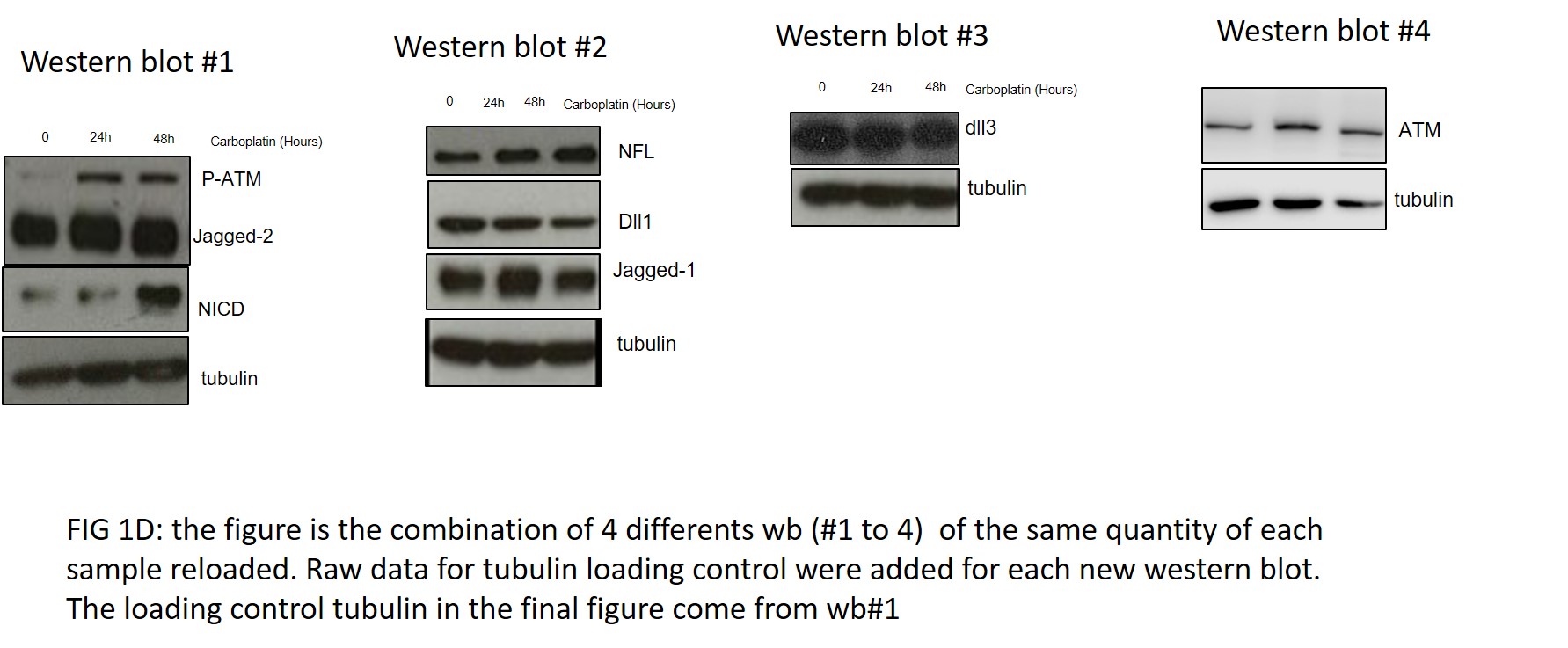

Supplement: Supplementary file 2 — Source data Fig. 1 [file 44321_2025_354_MOESM2_ESM.zip › Fig1/Fig 1D/FIG 1D.jpg]

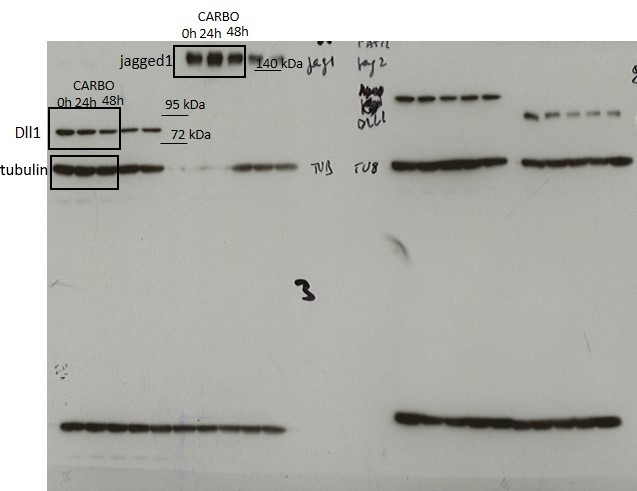

Supplement: Supplementary file 2 — Source data Fig. 1 [file 44321_2025_354_MOESM2_ESM.zip › Fig1/Fig 1D/jagged1, dll1, tubulin wb2.jpg]

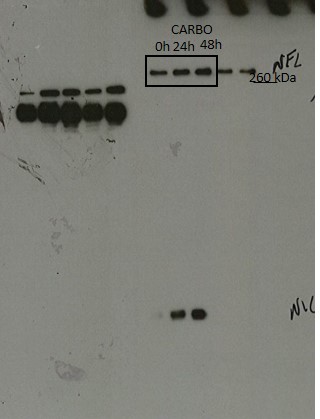

Supplement: Supplementary file 2 — Source data Fig. 1 [file 44321_2025_354_MOESM2_ESM.zip › Fig1/Fig 1D/NFL, wb2.jpg]

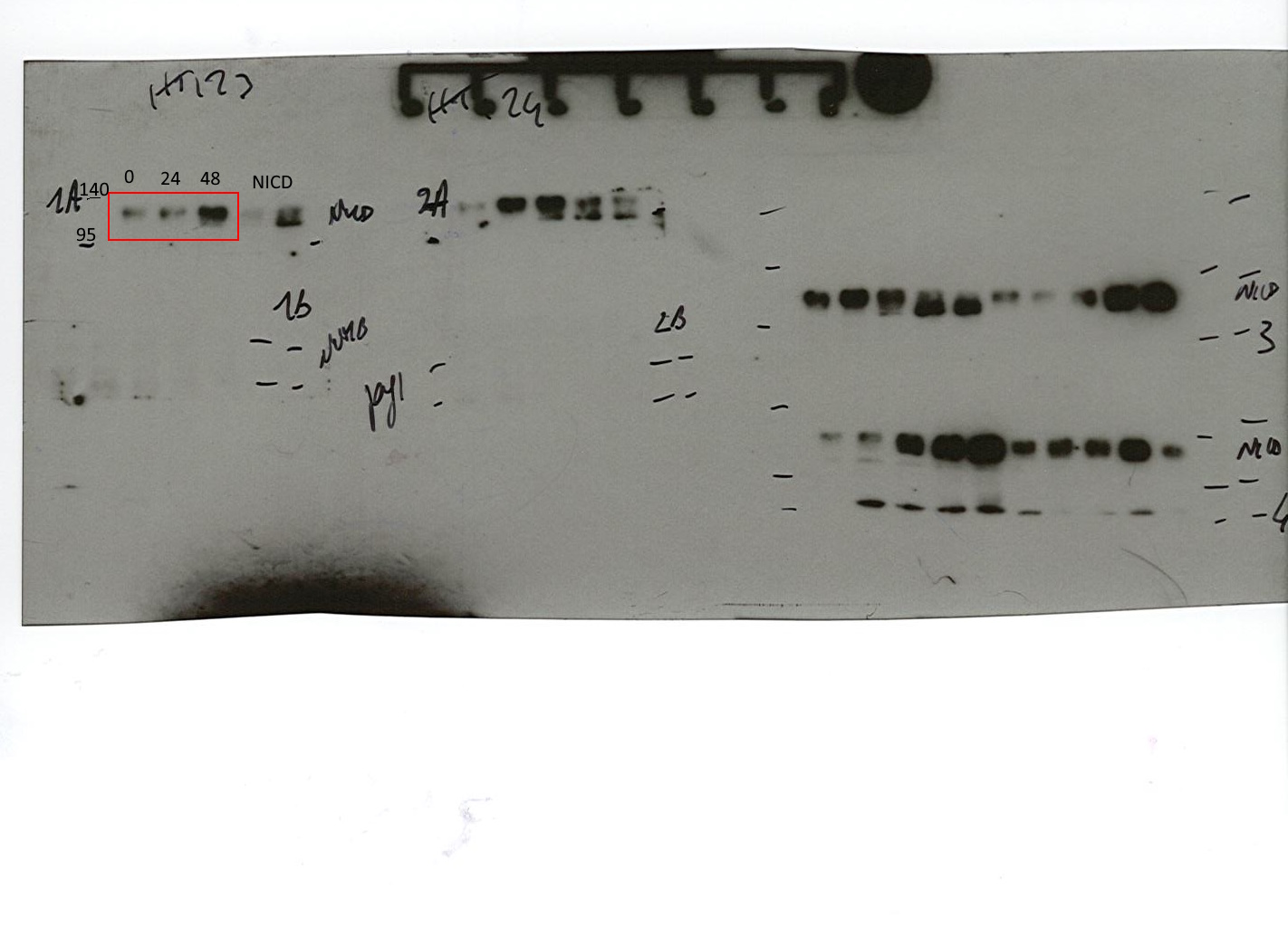

Supplement: Supplementary file 2 — Source data Fig. 1 [file 44321_2025_354_MOESM2_ESM.zip › Fig1/Fig 1D/nicd, wb1.jpg]

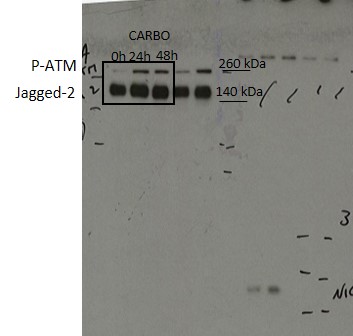

Supplement: Supplementary file 2 — Source data Fig. 1 [file 44321_2025_354_MOESM2_ESM.zip › Fig1/Fig 1D/pATM, jagged 2, wb 1.jpg]

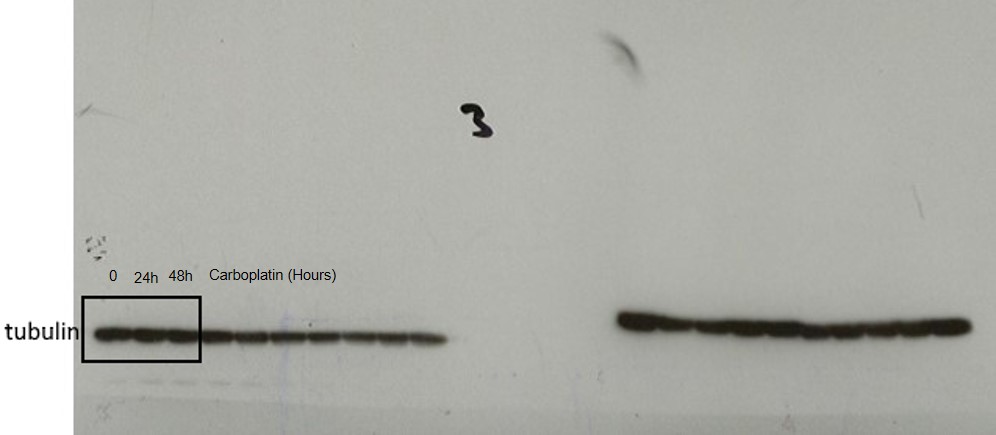

Supplement: Supplementary file 2 — Source data Fig. 1 [file 44321_2025_354_MOESM2_ESM.zip › Fig1/Fig 1D/tubulin wb3.jpg]

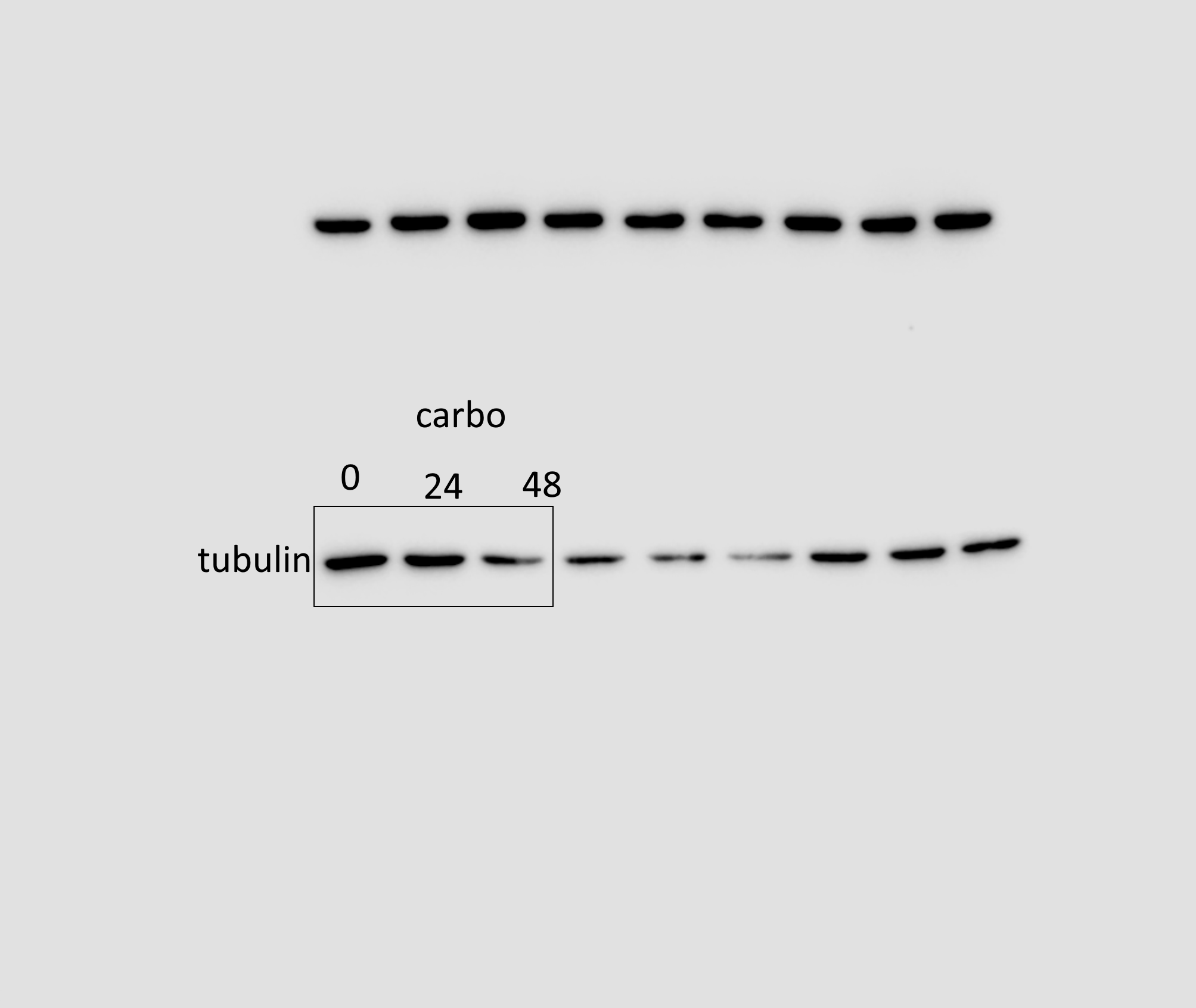

Supplement: Supplementary file 2 — Source data Fig. 1 [file 44321_2025_354_MOESM2_ESM.zip › Fig1/Fig 1D/Tubulin wb4.tif]

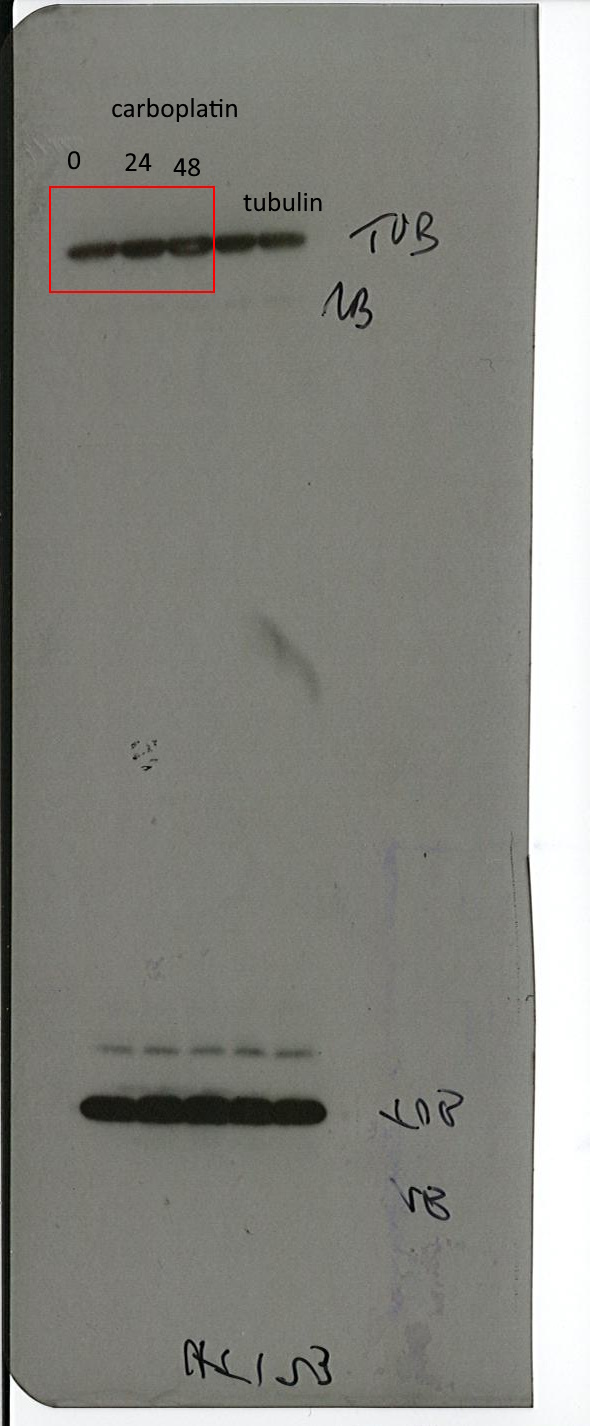

Supplement: Supplementary file 2 — Source data Fig. 1 [file 44321_2025_354_MOESM2_ESM.zip › Fig1/Fig 1D/tubulin, wb1.jpg]

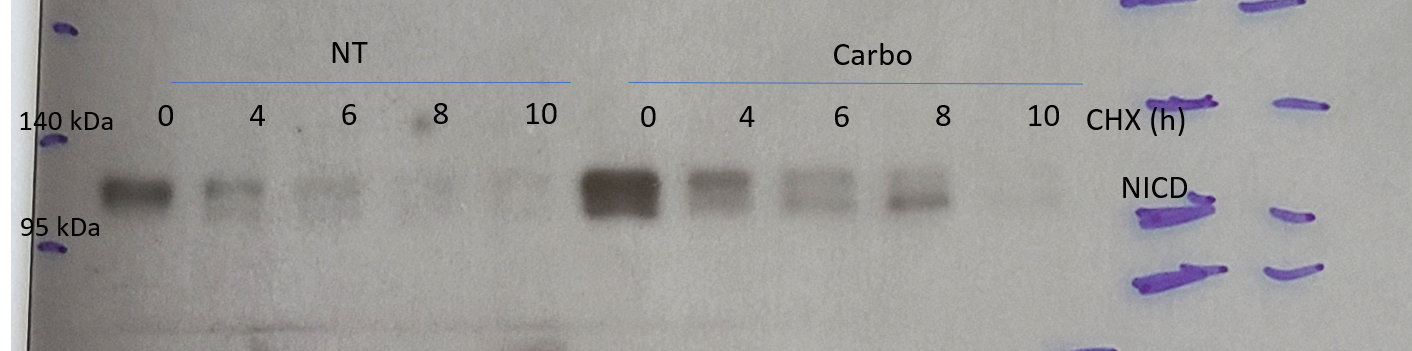

Supplement: Supplementary file 2 — Source data Fig. 1 [file 44321_2025_354_MOESM2_ESM.zip › Fig1/Fig 1E/Fig1E/Fig1E replicate/western blot nicd N2.png]

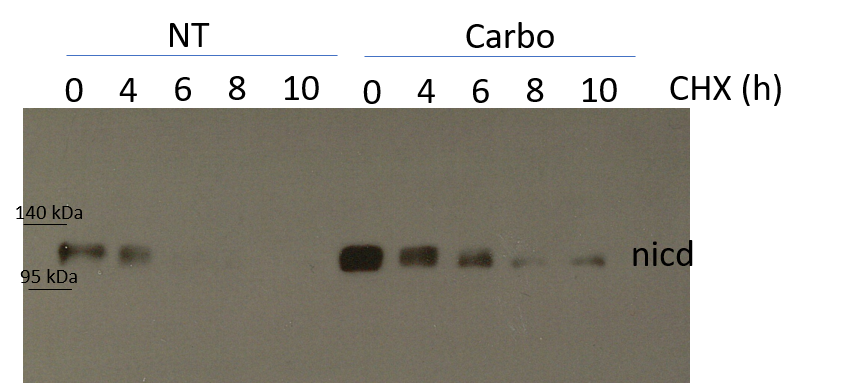

Supplement: Supplementary file 2 — Source data Fig. 1 [file 44321_2025_354_MOESM2_ESM.zip › Fig1/Fig 1E/Fig1E/Fig1E replicate/western blot nicd n3.png]

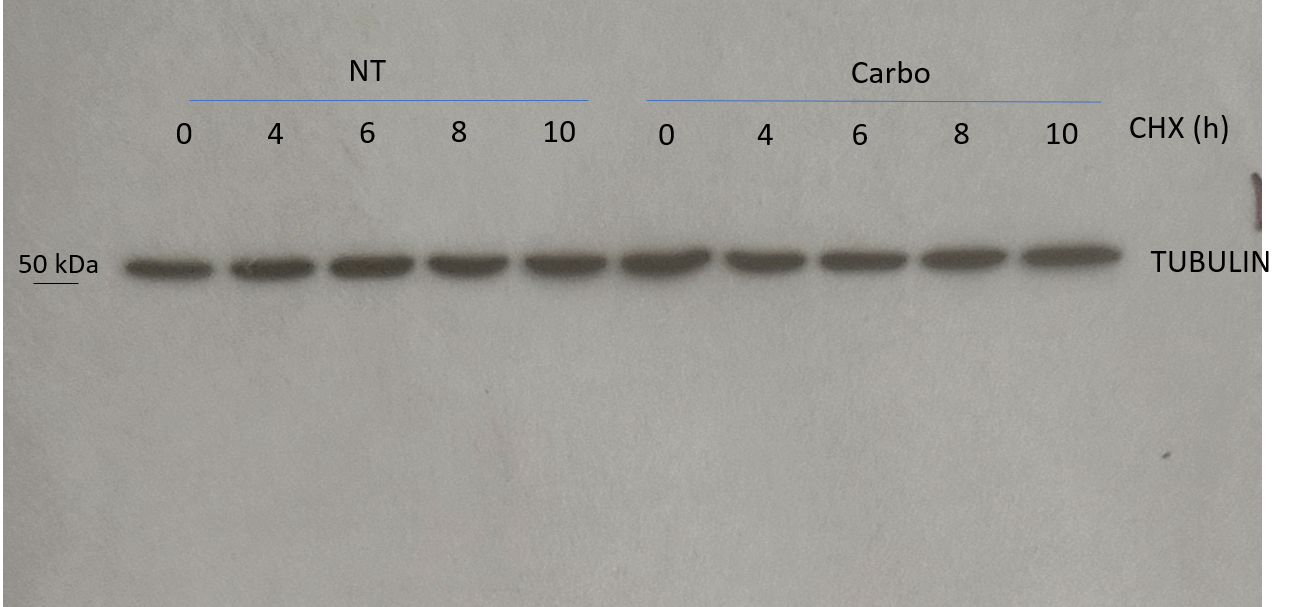

Supplement: Supplementary file 2 — Source data Fig. 1 [file 44321_2025_354_MOESM2_ESM.zip › Fig1/Fig 1E/Fig1E/Fig1E replicate/western blot tubulin N2.png]

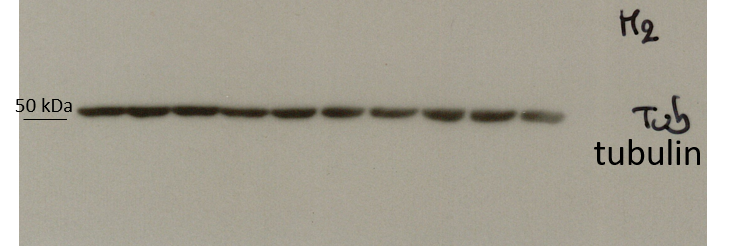

Supplement: Supplementary file 2 — Source data Fig. 1 [file 44321_2025_354_MOESM2_ESM.zip › Fig1/Fig 1E/Fig1E/Fig1E replicate/western blot tubulin N3.png]

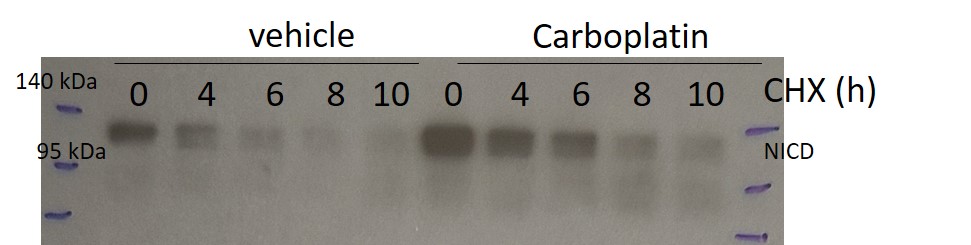

Supplement: Supplementary file 2 — Source data Fig. 1 [file 44321_2025_354_MOESM2_ESM.zip › Fig1/Fig 1E/Fig1E/western blot nicd.jpg]

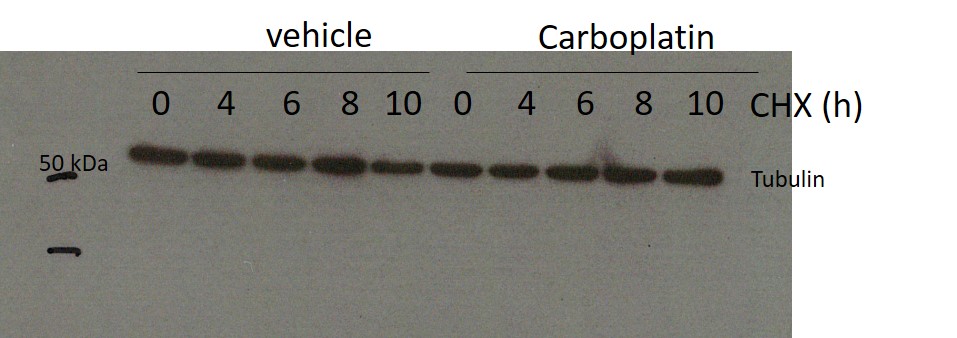

Supplement: Supplementary file 2 — Source data Fig. 1 [file 44321_2025_354_MOESM2_ESM.zip › Fig1/Fig 1E/Fig1E/western blot tubulin.jpg]

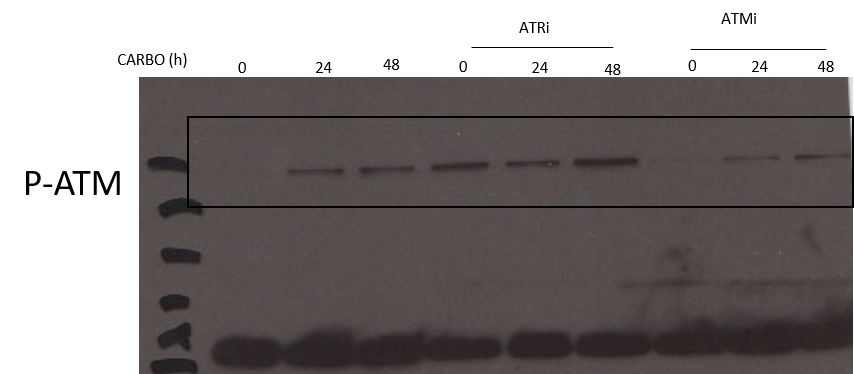

Supplement: Supplementary file 3 — Source data Fig. 2 [file 44321_2025_354_MOESM3_ESM.zip › Fig2/Fig2A/Fig 2A replicate/western blot P-ATM N2.png]

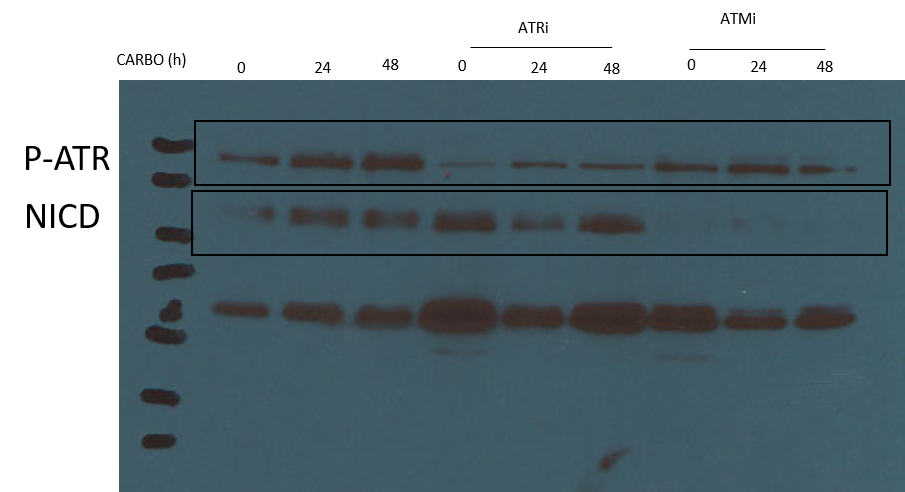

Supplement: Supplementary file 3 — Source data Fig. 2 [file 44321_2025_354_MOESM3_ESM.zip › Fig2/Fig2A/Fig 2A replicate/western blot P-ATR, NICD N2.png]

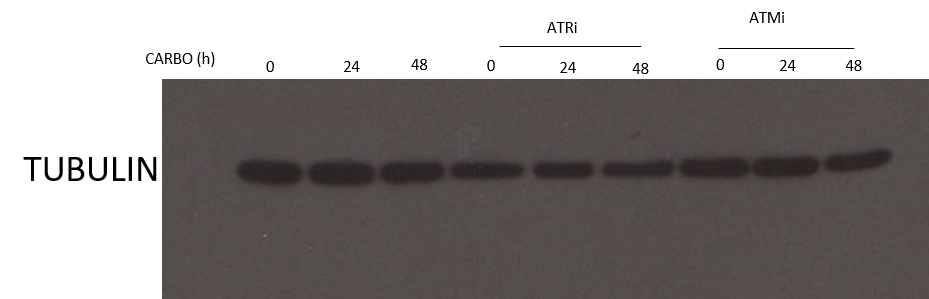

Supplement: Supplementary file 3 — Source data Fig. 2 [file 44321_2025_354_MOESM3_ESM.zip › Fig2/Fig2A/Fig 2A replicate/western blot tubulin N2.png]

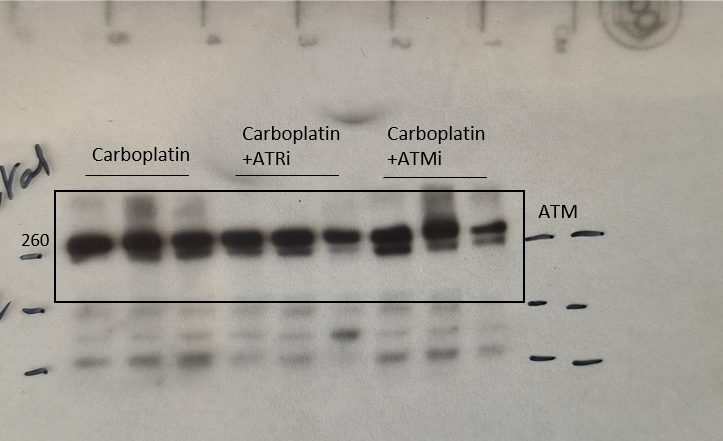

Supplement: Supplementary file 3 — Source data Fig. 2 [file 44321_2025_354_MOESM3_ESM.zip › Fig2/Fig2A/western blot-ATM.png]

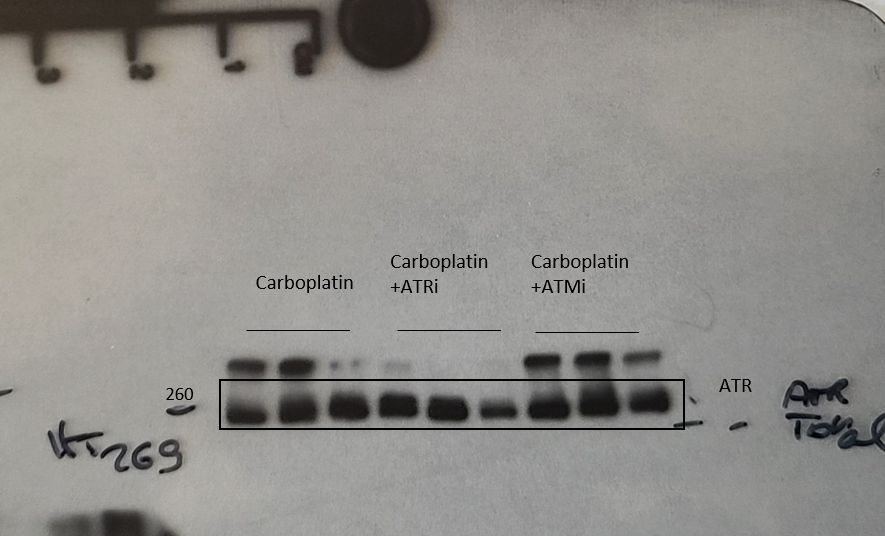

Supplement: Supplementary file 3 — Source data Fig. 2 [file 44321_2025_354_MOESM3_ESM.zip › Fig2/Fig2A/western blot-ATR.png]

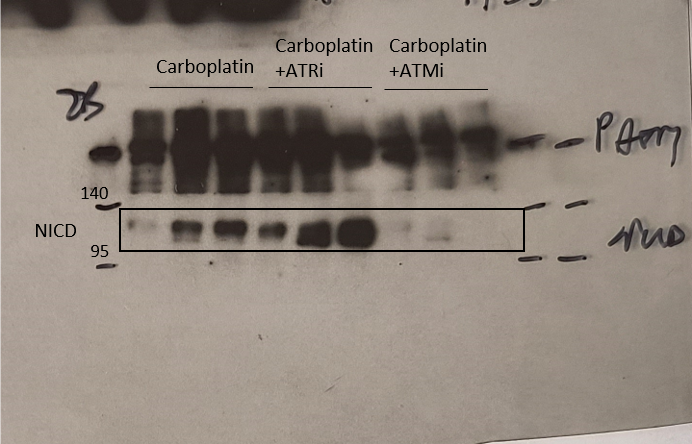

Supplement: Supplementary file 3 — Source data Fig. 2 [file 44321_2025_354_MOESM3_ESM.zip › Fig2/Fig2A/western blot-NICD.png]

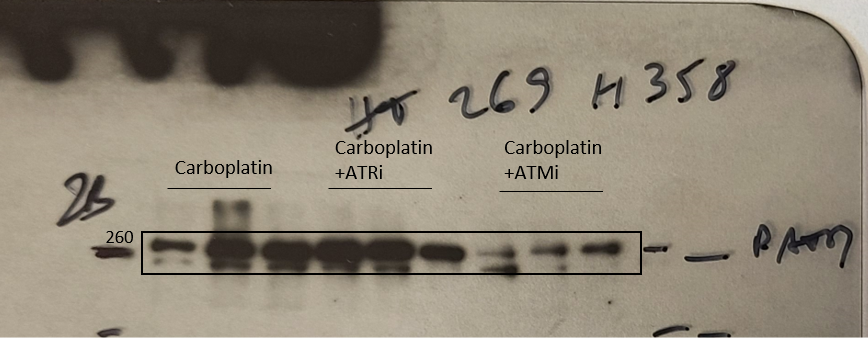

Supplement: Supplementary file 3 — Source data Fig. 2 [file 44321_2025_354_MOESM3_ESM.zip › Fig2/Fig2A/western blot-P-ATM.png]

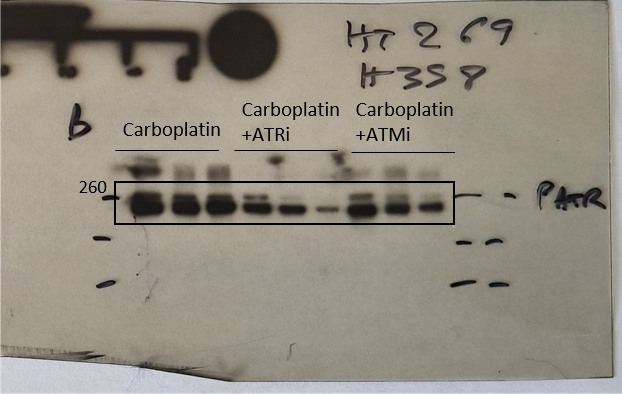

Supplement: Supplementary file 3 — Source data Fig. 2 [file 44321_2025_354_MOESM3_ESM.zip › Fig2/Fig2A/western blot-P-ATR.png]

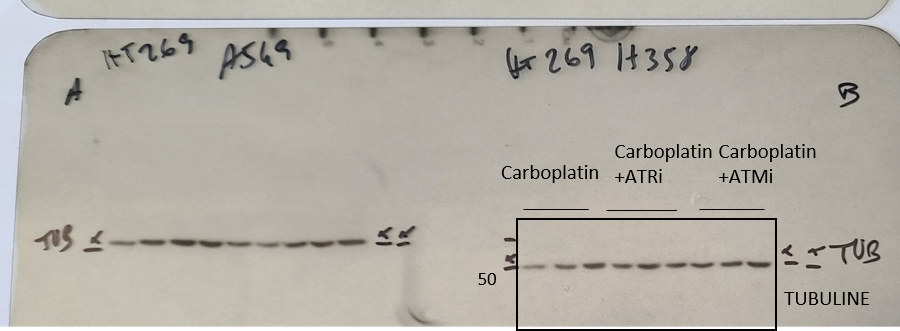

Supplement: Supplementary file 3 — Source data Fig. 2 [file 44321_2025_354_MOESM3_ESM.zip › Fig2/Fig2A/western blot-tubulin.png]

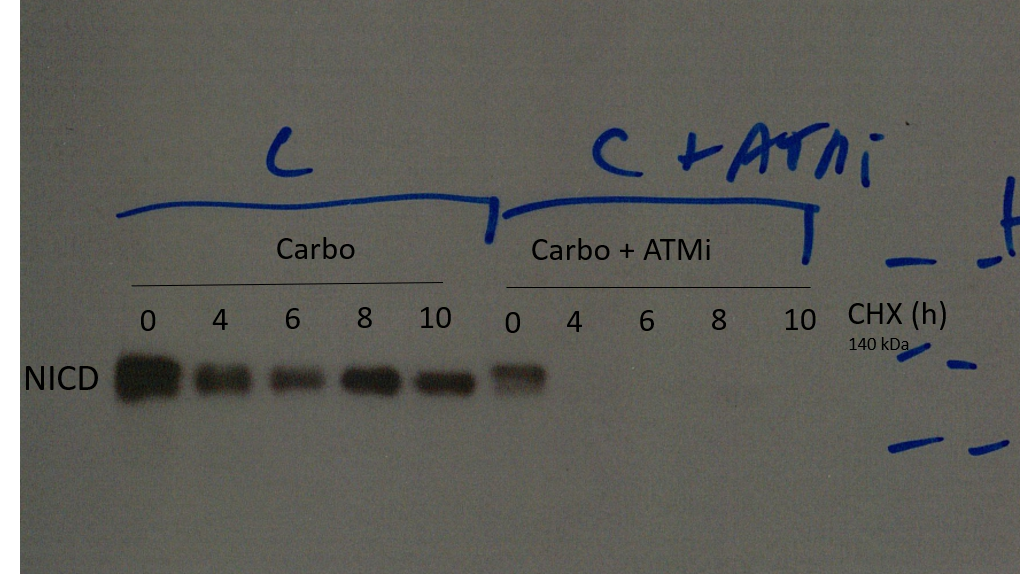

Supplement: Supplementary file 3 — Source data Fig. 2 [file 44321_2025_354_MOESM3_ESM.zip › Fig2/Fig2B/Fig2B replicat/WESTERN BLOT NICD N2.png]

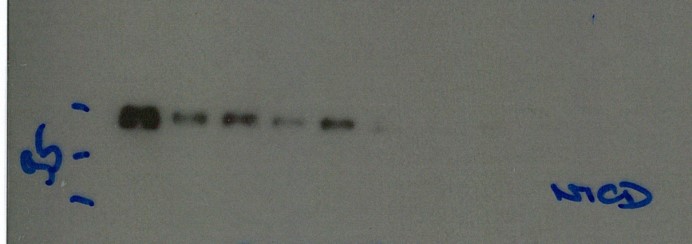

Supplement: Supplementary file 3 — Source data Fig. 2 [file 44321_2025_354_MOESM3_ESM.zip › Fig2/Fig2B/Fig2B replicat/western blot nicd N3.jpg]

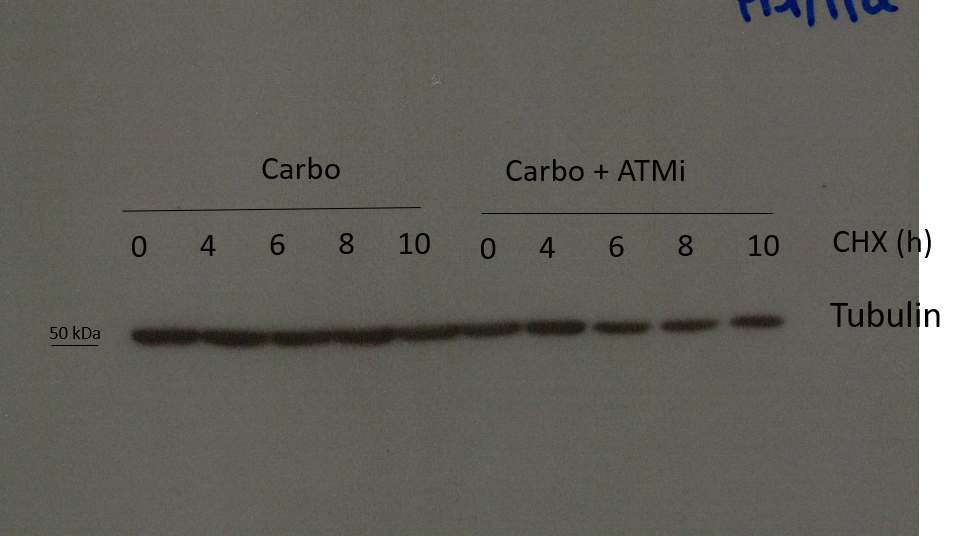

Supplement: Supplementary file 3 — Source data Fig. 2 [file 44321_2025_354_MOESM3_ESM.zip › Fig2/Fig2B/Fig2B replicat/WESTERN BLOT TUBULIN N2.png]

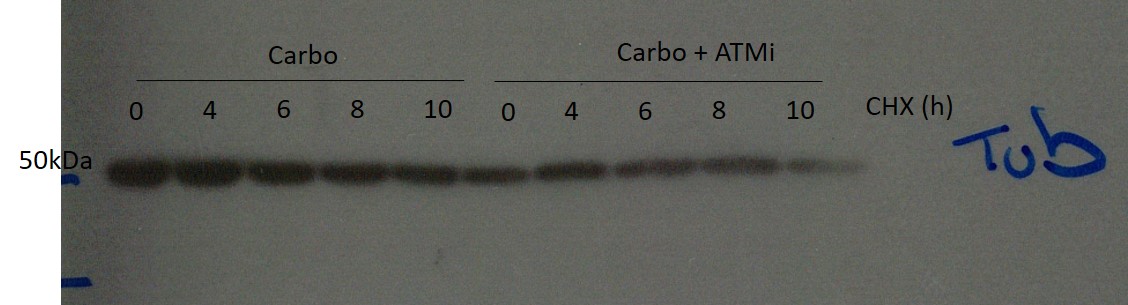

Supplement: Supplementary file 3 — Source data Fig. 2 [file 44321_2025_354_MOESM3_ESM.zip › Fig2/Fig2B/Fig2B replicat/western blot tubulin N3.jpg]

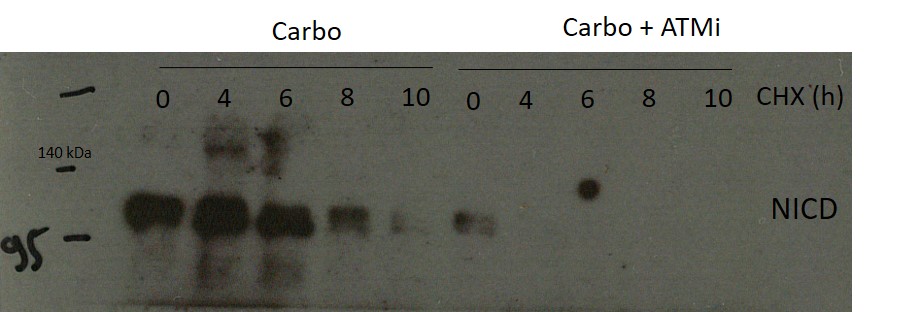

Supplement: Supplementary file 3 — Source data Fig. 2 [file 44321_2025_354_MOESM3_ESM.zip › Fig2/Fig2B/western blot nicd.jpg]

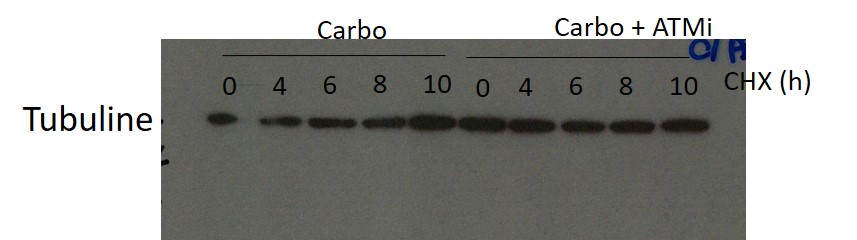

Supplement: Supplementary file 3 — Source data Fig. 2 [file 44321_2025_354_MOESM3_ESM.zip › Fig2/Fig2B/western blot tubulin.jpg]

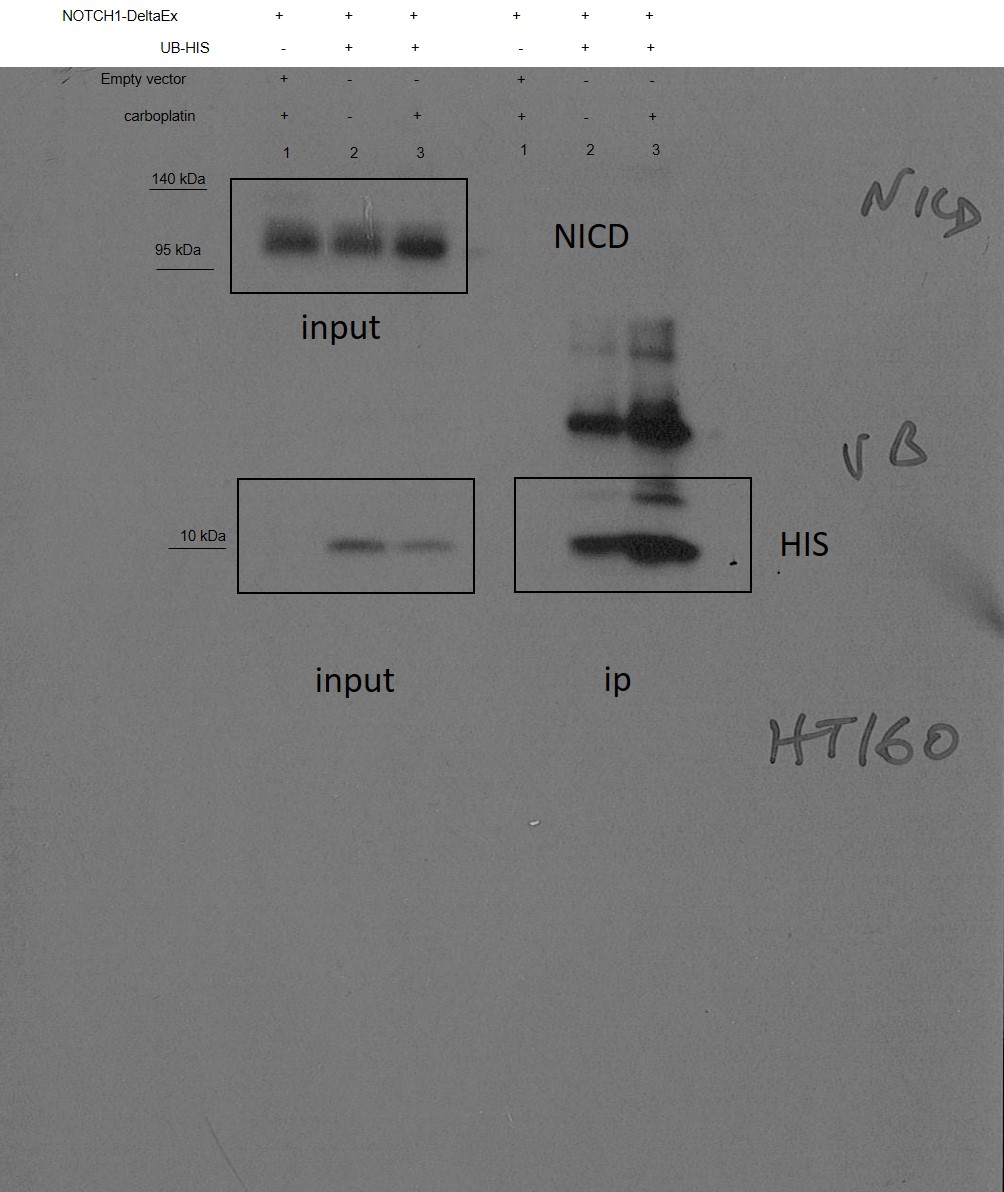

Supplement: Supplementary file 4 — Source data Fig. 3 [file 44321_2025_354_MOESM4_ESM.zip › Fig 3/Fig 3A/Fig 3A replicat/western blot NICD, HIS input, HIS pulldown N3.jpg]

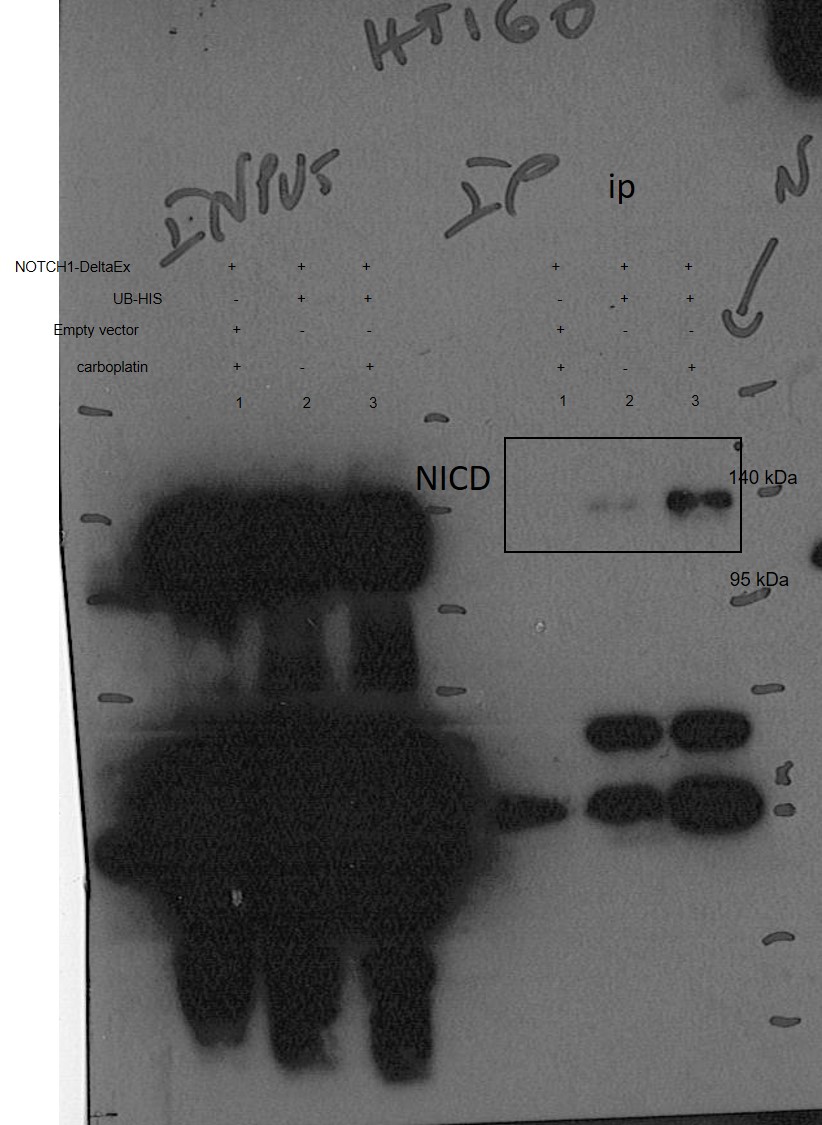

Supplement: Supplementary file 4 — Source data Fig. 3 [file 44321_2025_354_MOESM4_ESM.zip › Fig 3/Fig 3A/Fig 3A replicat/wetsern blot nicd pulldown N3.jpg]

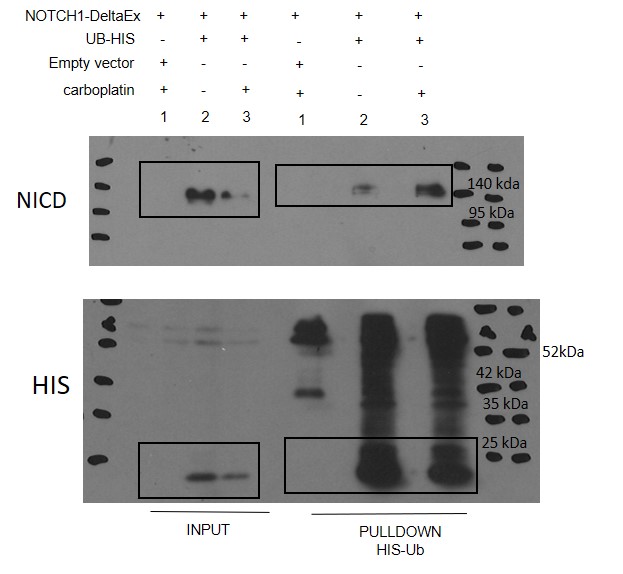

Supplement: Supplementary file 4 — Source data Fig. 3 [file 44321_2025_354_MOESM4_ESM.zip › Fig 3/Fig 3A/Fig 3A replicat/wetsern blot nicd, HIS N2.jpg]

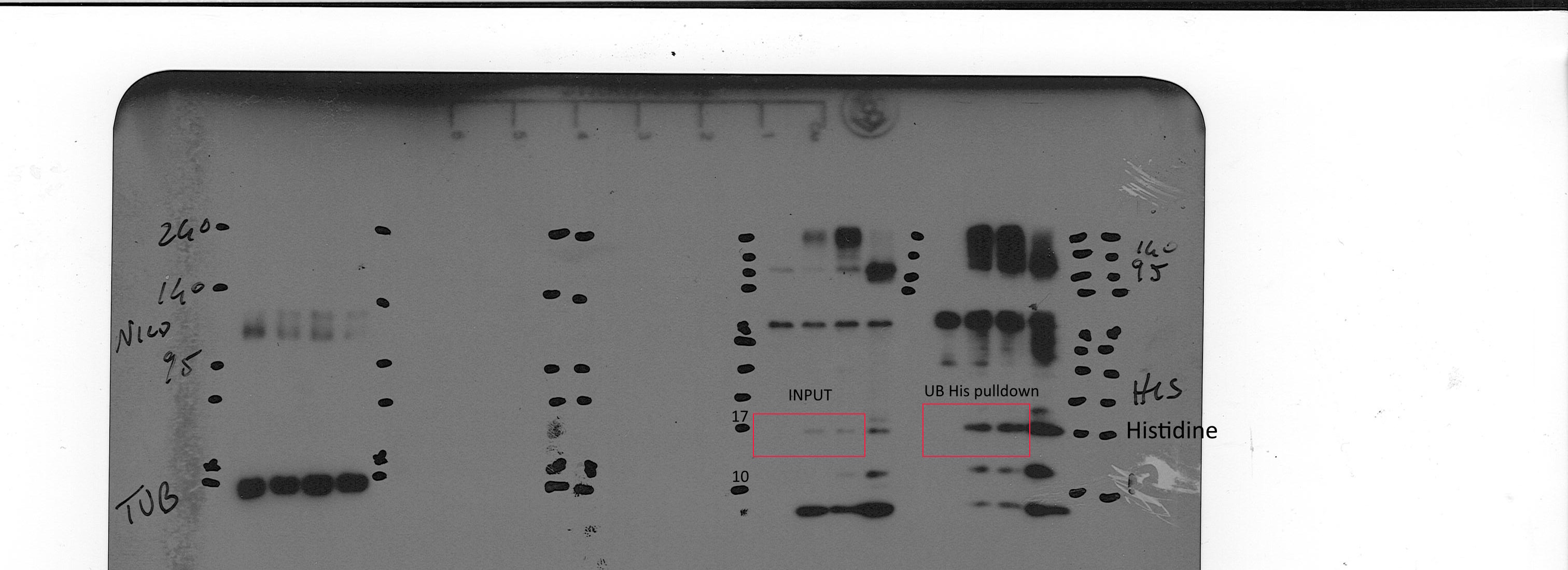

Supplement: Supplementary file 4 — Source data Fig. 3 [file 44321_2025_354_MOESM4_ESM.zip › Fig 3/Fig 3A/western histidine.jpg]

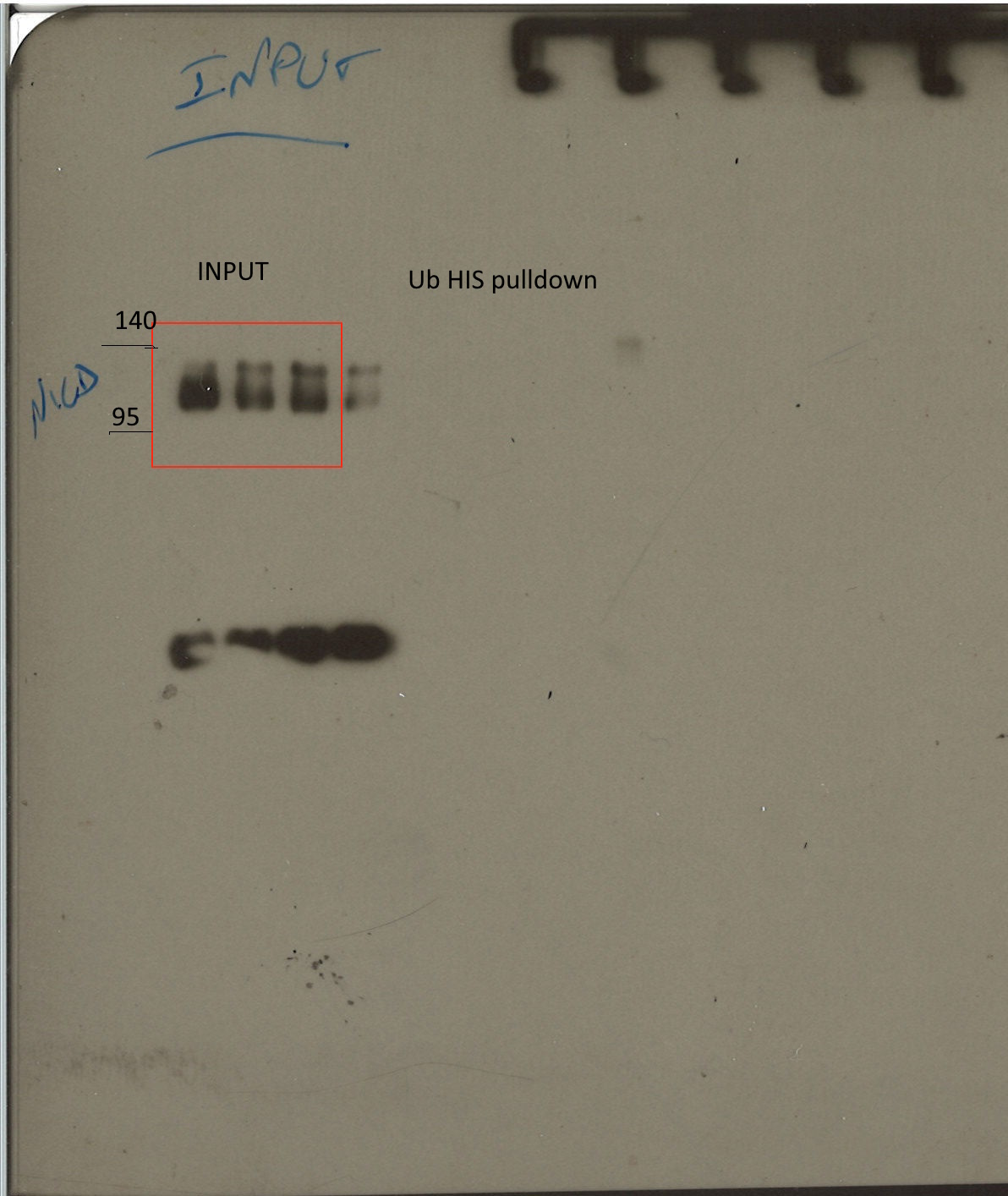

Supplement: Supplementary file 4 — Source data Fig. 3 [file 44321_2025_354_MOESM4_ESM.zip › Fig 3/Fig 3A/western NICD input.jpg]

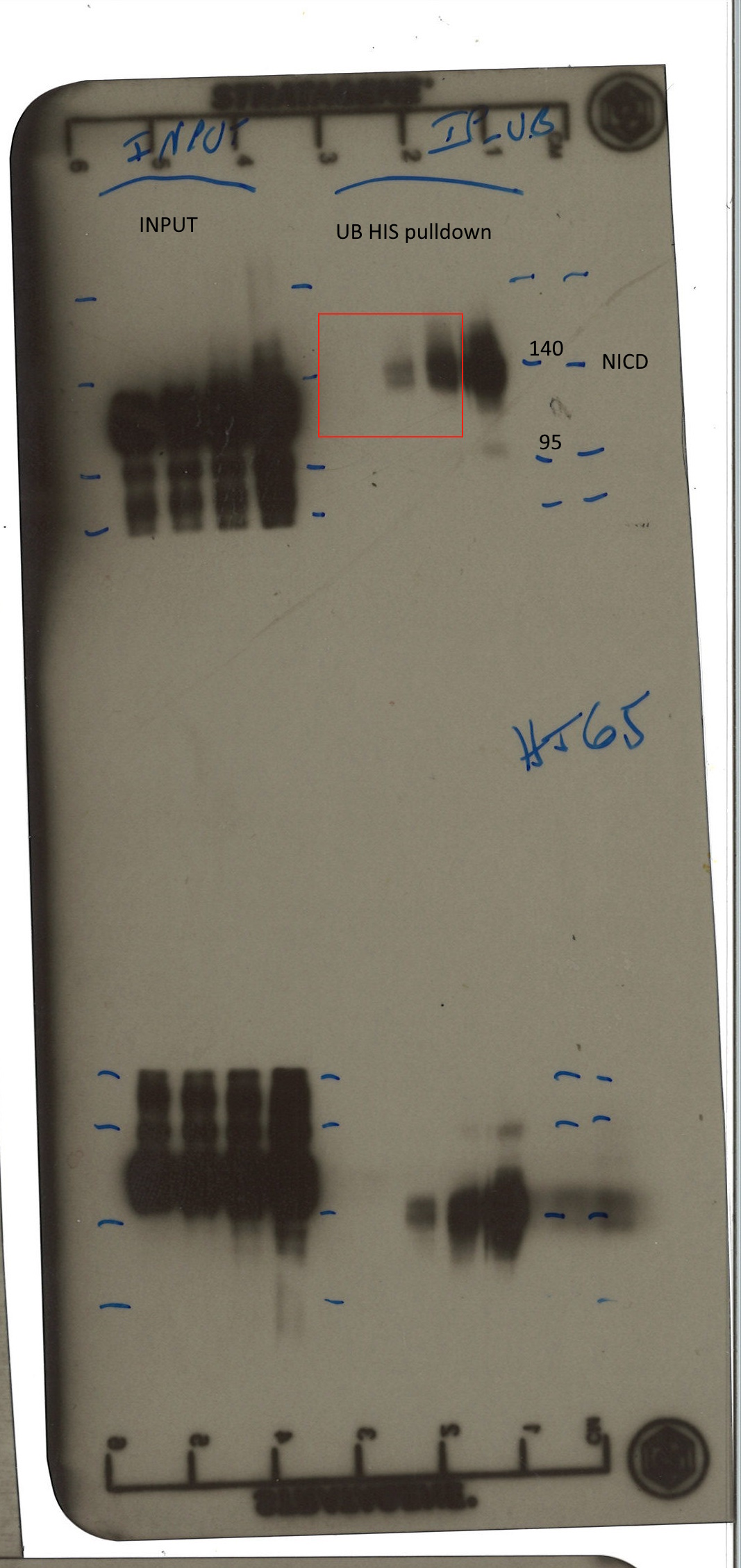

Supplement: Supplementary file 4 — Source data Fig. 3 [file 44321_2025_354_MOESM4_ESM.zip › Fig 3/Fig 3A/western NICD pulldown.jpg]

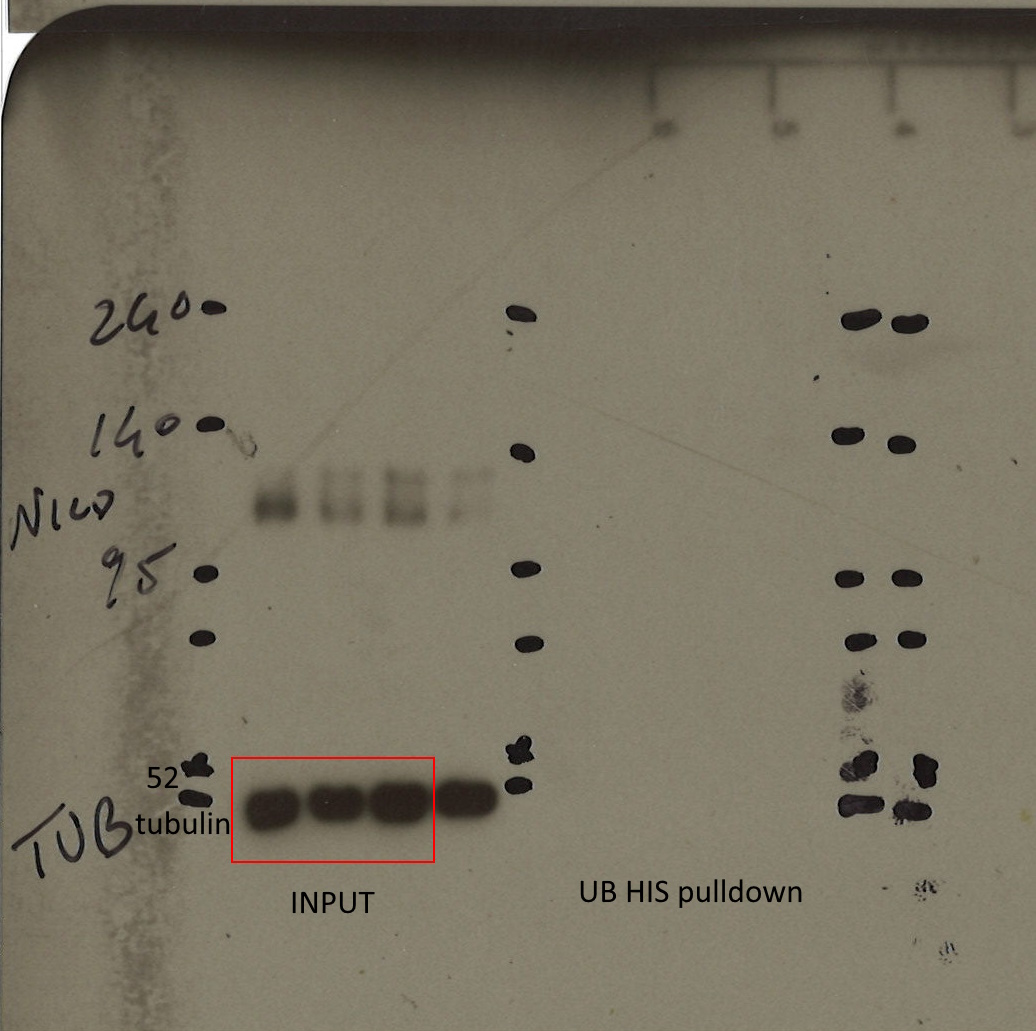

Supplement: Supplementary file 4 — Source data Fig. 3 [file 44321_2025_354_MOESM4_ESM.zip › Fig 3/Fig 3A/western tubulin.jpg]

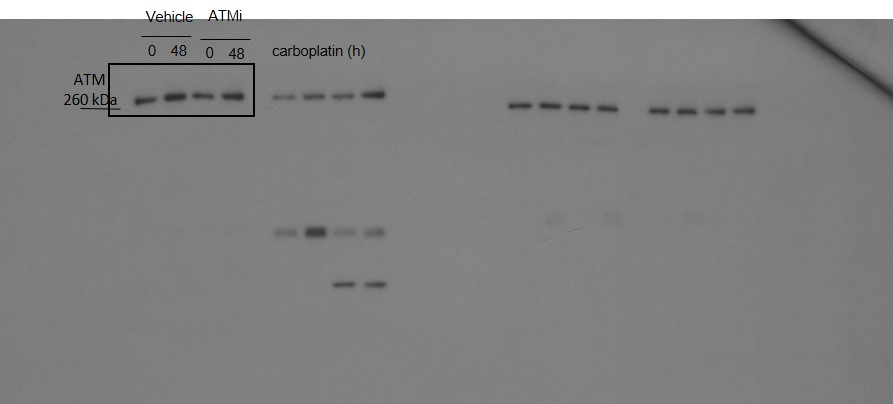

Supplement: Supplementary file 4 — Source data Fig. 3 [file 44321_2025_354_MOESM4_ESM.zip › Fig 3/Fig 3B/Fig 3B replicat/western blot ATM N2.jpg]

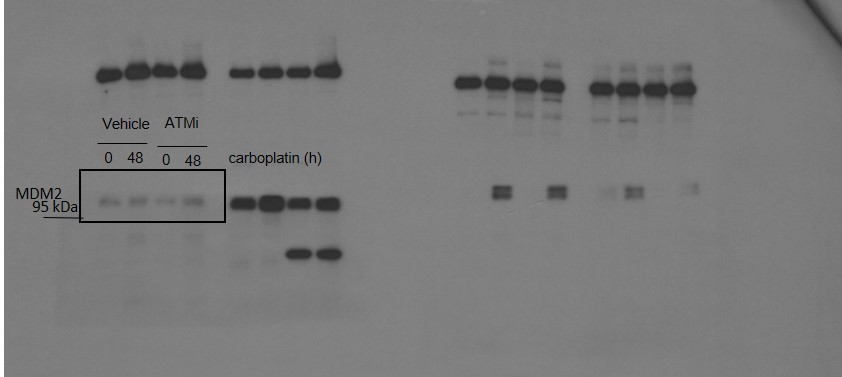

Supplement: Supplementary file 4 — Source data Fig. 3 [file 44321_2025_354_MOESM4_ESM.zip › Fig 3/Fig 3B/Fig 3B replicat/western blot MDM2 N2.jpg]

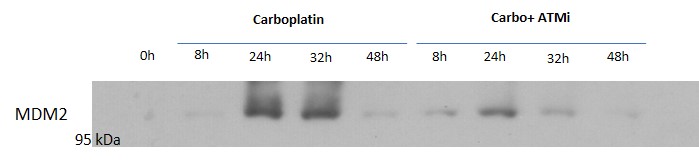

Supplement: Supplementary file 4 — Source data Fig. 3 [file 44321_2025_354_MOESM4_ESM.zip › Fig 3/Fig 3B/Fig 3B replicat/western blot MDM2 N3.jpg]

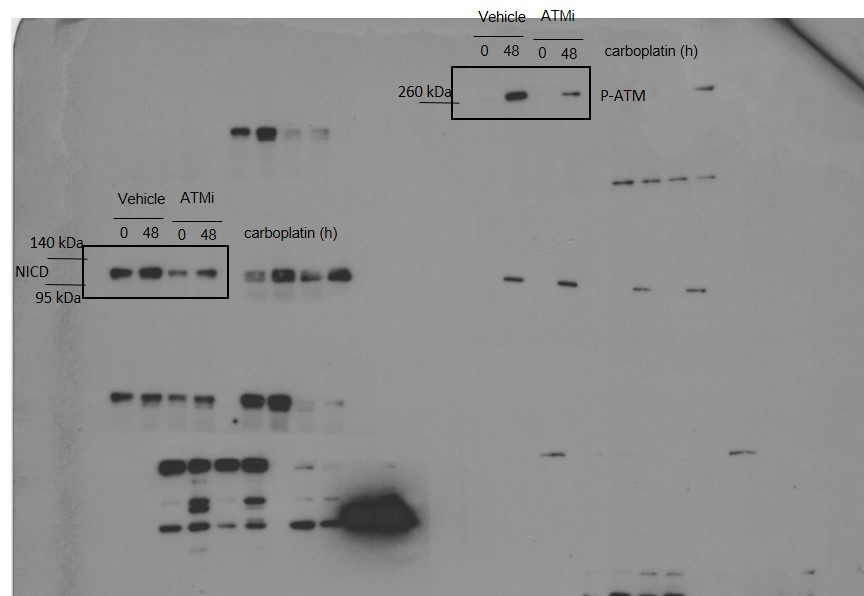

Supplement: Supplementary file 4 — Source data Fig. 3 [file 44321_2025_354_MOESM4_ESM.zip › Fig 3/Fig 3B/Fig 3B replicat/western blot nicd, P-ATM N2.jpg]

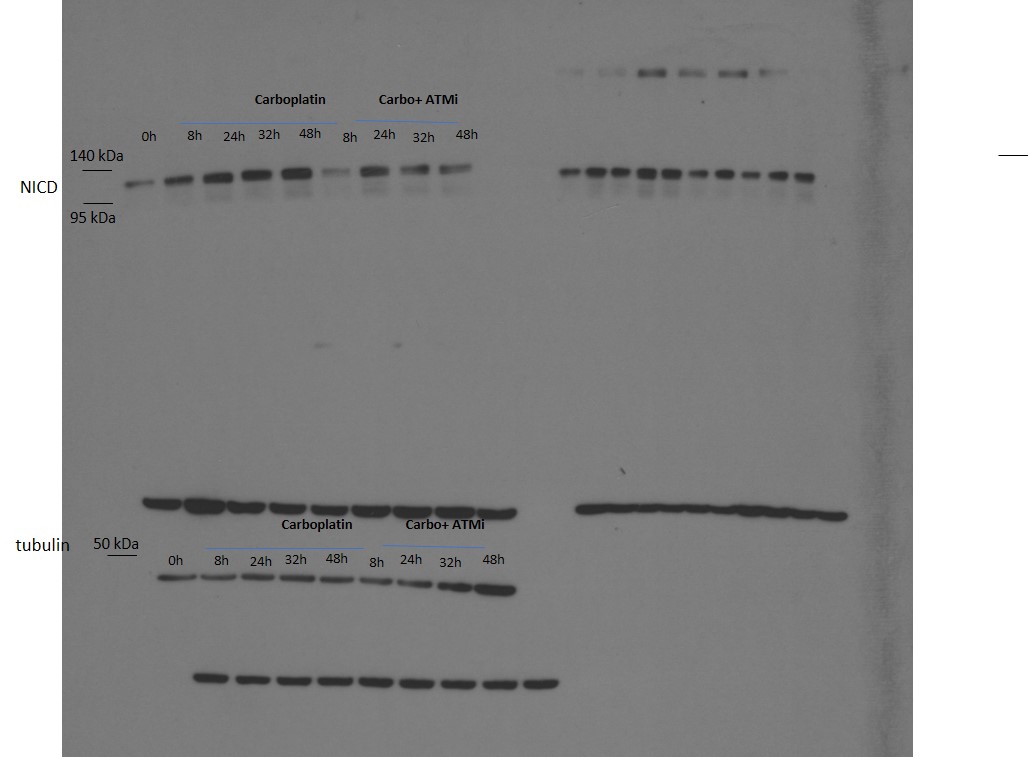

Supplement: Supplementary file 4 — Source data Fig. 3 [file 44321_2025_354_MOESM4_ESM.zip › Fig 3/Fig 3B/Fig 3B replicat/western blot nicd,tubulin N3.jpg]

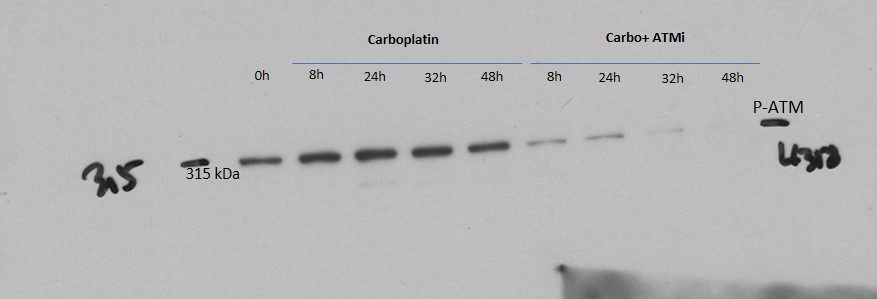

Supplement: Supplementary file 4 — Source data Fig. 3 [file 44321_2025_354_MOESM4_ESM.zip › Fig 3/Fig 3B/Fig 3B replicat/western blot P-ATM N3.jpg]

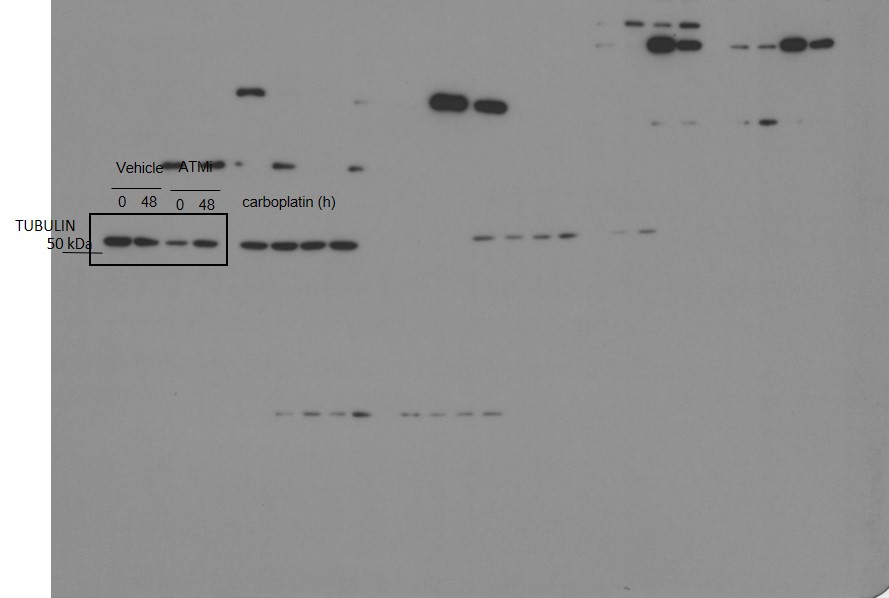

Supplement: Supplementary file 4 — Source data Fig. 3 [file 44321_2025_354_MOESM4_ESM.zip › Fig 3/Fig 3B/Fig 3B replicat/western blot TUBULIN N2.jpg]

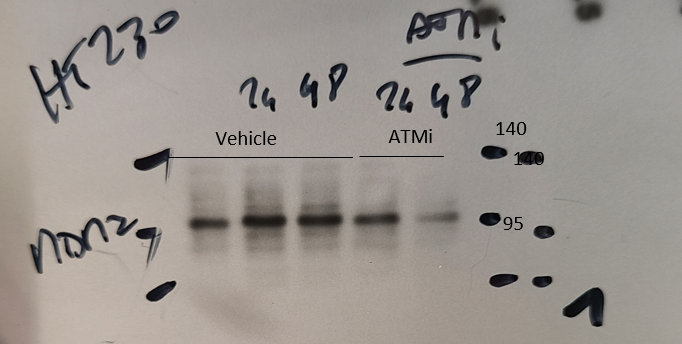

Supplement: Supplementary file 4 — Source data Fig. 3 [file 44321_2025_354_MOESM4_ESM.zip › Fig 3/Fig 3B/western blot MDM2.png]

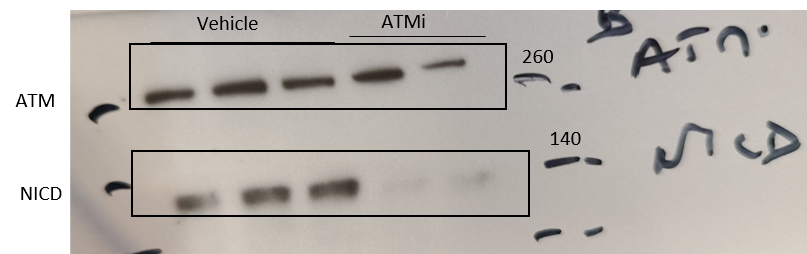

Supplement: Supplementary file 4 — Source data Fig. 3 [file 44321_2025_354_MOESM4_ESM.zip › Fig 3/Fig 3B/western blot NICD, ATM.png]

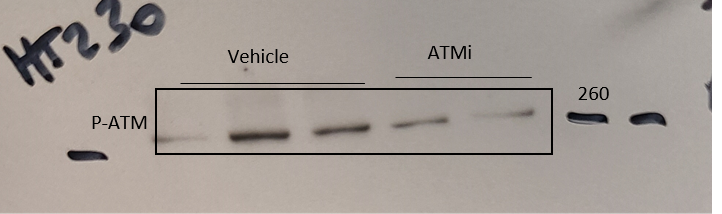

Supplement: Supplementary file 4 — Source data Fig. 3 [file 44321_2025_354_MOESM4_ESM.zip › Fig 3/Fig 3B/western blot P-ATM.png]

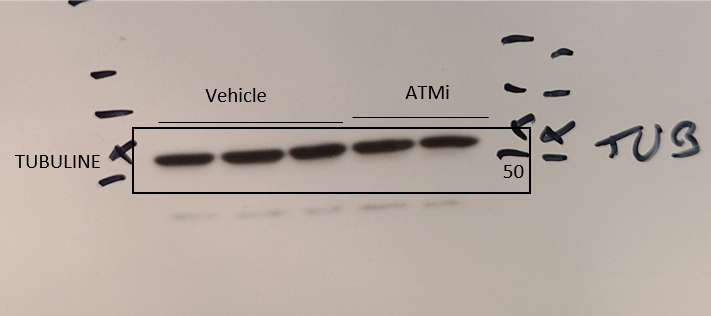

Supplement: Supplementary file 4 — Source data Fig. 3 [file 44321_2025_354_MOESM4_ESM.zip › Fig 3/Fig 3B/western blot TUBULIN.png]

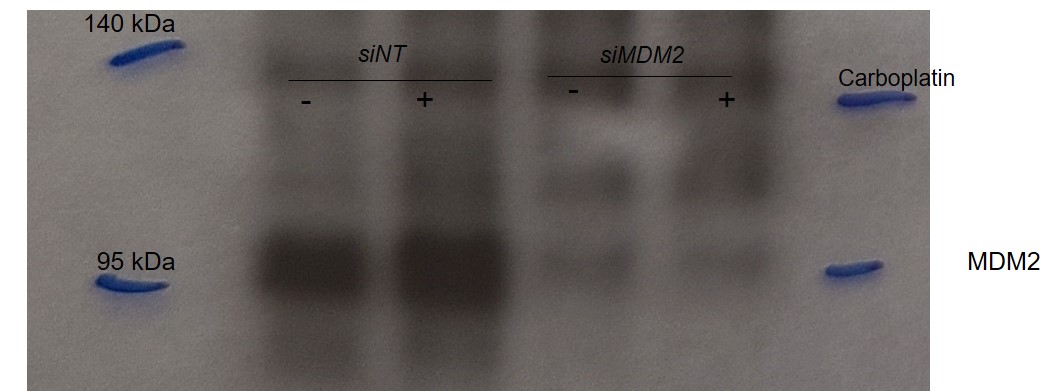

Supplement: Supplementary file 4 — Source data Fig. 3 [file 44321_2025_354_MOESM4_ESM.zip › Fig 3/Fig 3C/Fig 3c replicat/western blot mdm2 N2.jpg]

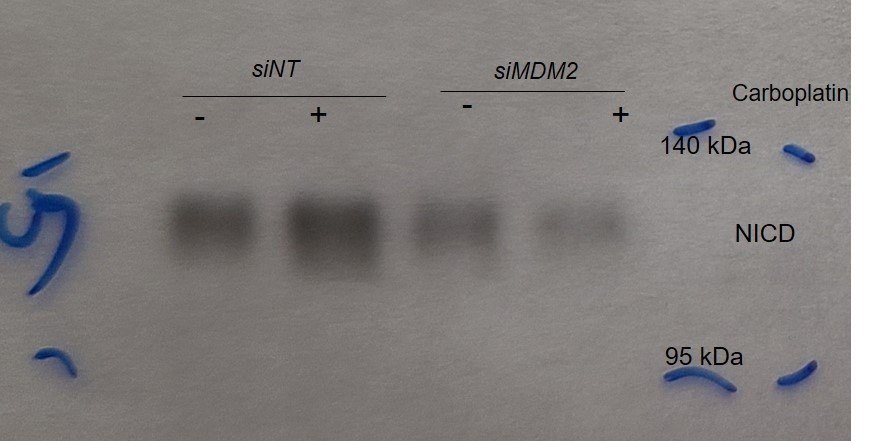

Supplement: Supplementary file 4 — Source data Fig. 3 [file 44321_2025_354_MOESM4_ESM.zip › Fig 3/Fig 3C/Fig 3c replicat/western blot nicd N2.jpg]

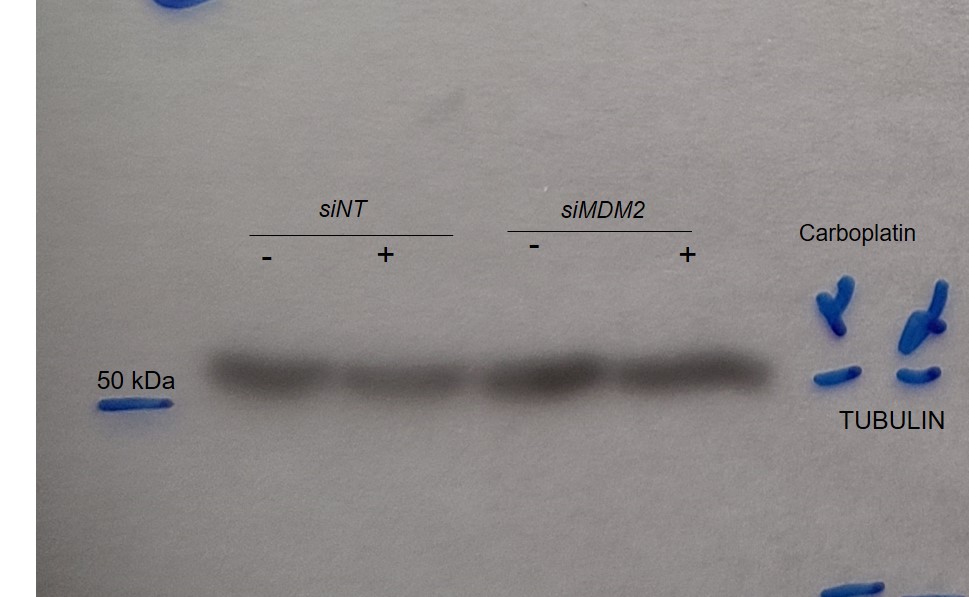

Supplement: Supplementary file 4 — Source data Fig. 3 [file 44321_2025_354_MOESM4_ESM.zip › Fig 3/Fig 3C/Fig 3c replicat/western blot tubulin N2.jpg]

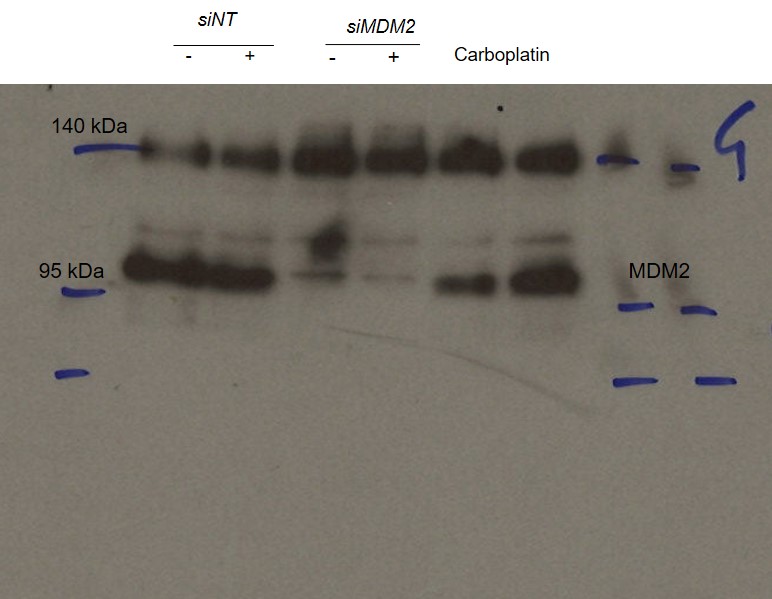

Supplement: Supplementary file 4 — Source data Fig. 3 [file 44321_2025_354_MOESM4_ESM.zip › Fig 3/Fig 3C/western blot MDM2.jpg]

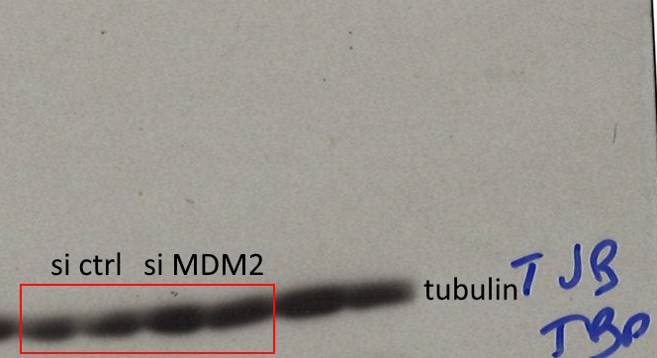

Supplement: Supplementary file 4 — Source data Fig. 3 [file 44321_2025_354_MOESM4_ESM.zip › Fig 3/Fig 3C/western blot tubulin.png]

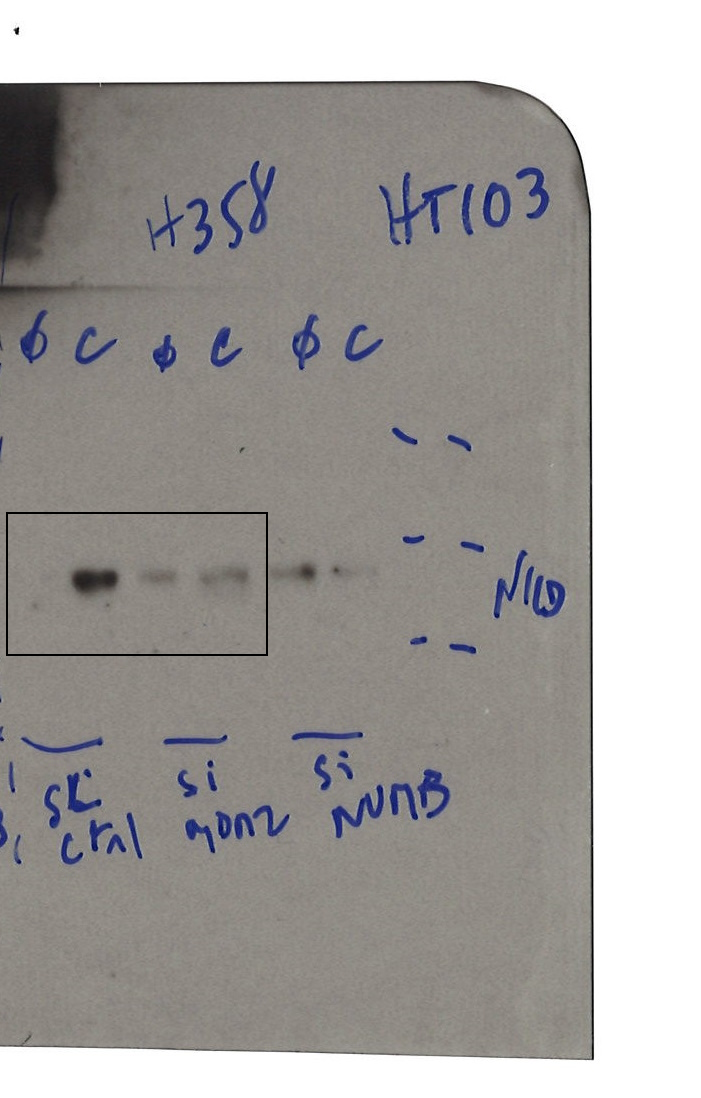

Supplement: Supplementary file 4 — Source data Fig. 3 [file 44321_2025_354_MOESM4_ESM.zip › Fig 3/Fig 3C/western NICD.jpg]

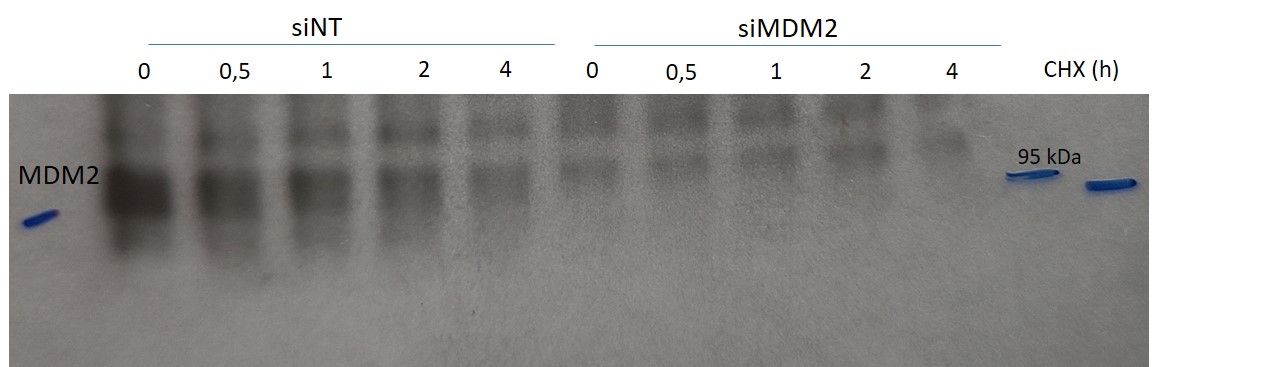

Supplement: Supplementary file 4 — Source data Fig. 3 [file 44321_2025_354_MOESM4_ESM.zip › Fig 3/Fig 3D/Fig 3D replicate/western blot MDM2 N2.jpg]

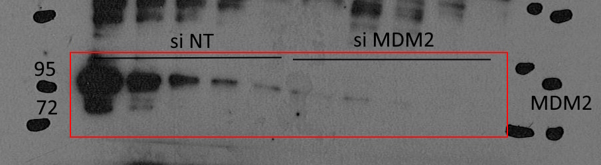

Supplement: Supplementary file 4 — Source data Fig. 3 [file 44321_2025_354_MOESM4_ESM.zip › Fig 3/Fig 3D/Fig 3D replicate/western blot MDM2 N3.png]

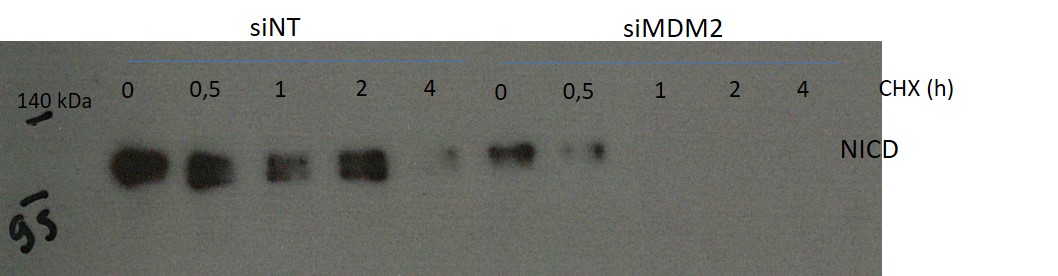

Supplement: Supplementary file 4 — Source data Fig. 3 [file 44321_2025_354_MOESM4_ESM.zip › Fig 3/Fig 3D/Fig 3D replicate/WESTERN BLOT NICD N2.jpg]

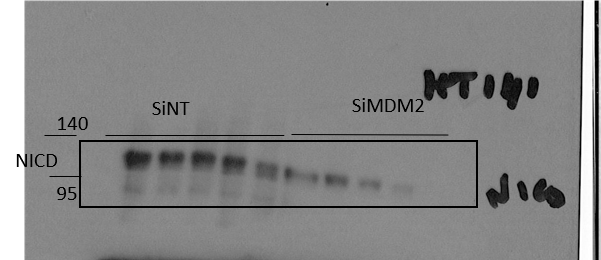

Supplement: Supplementary file 4 — Source data Fig. 3 [file 44321_2025_354_MOESM4_ESM.zip › Fig 3/Fig 3D/Fig 3D replicate/western blot nicd N3.png]

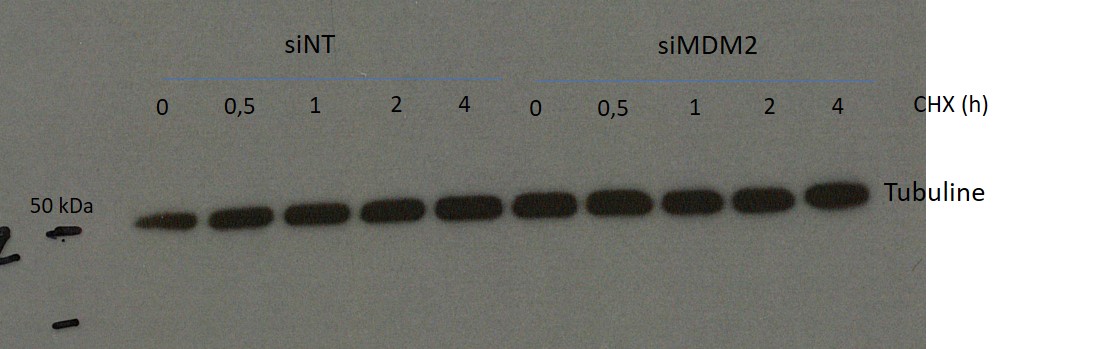

Supplement: Supplementary file 4 — Source data Fig. 3 [file 44321_2025_354_MOESM4_ESM.zip › Fig 3/Fig 3D/Fig 3D replicate/WESTERN BLOT TUBULIN N2.jpg]

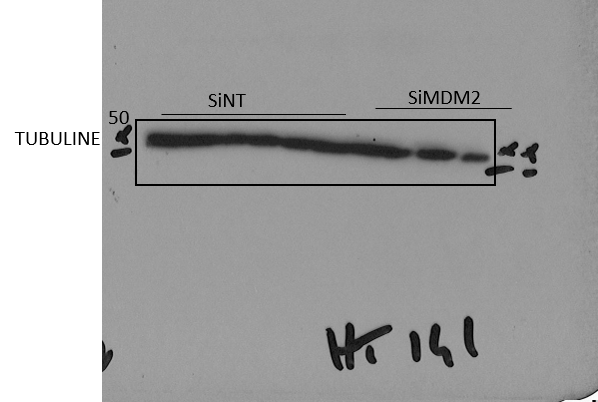

Supplement: Supplementary file 4 — Source data Fig. 3 [file 44321_2025_354_MOESM4_ESM.zip › Fig 3/Fig 3D/Fig 3D replicate/western blot tubulin N3.png]

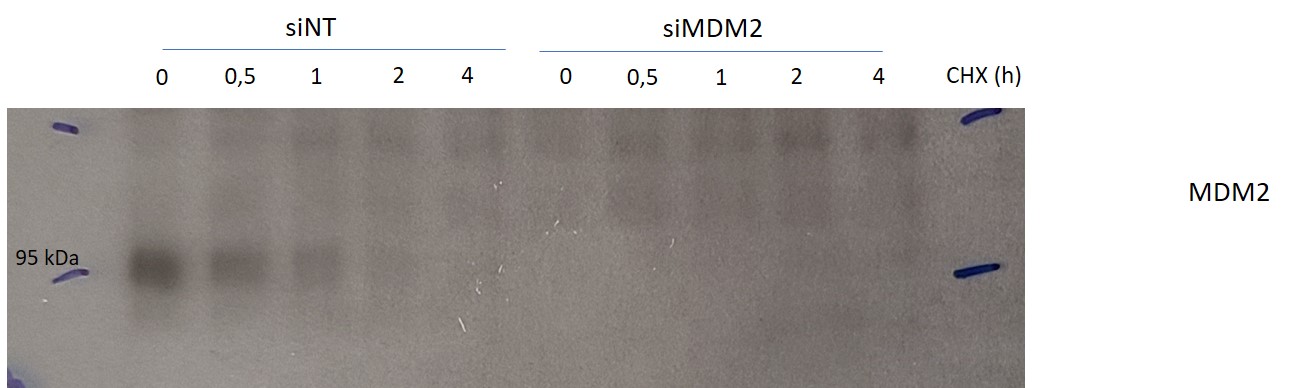

Supplement: Supplementary file 4 — Source data Fig. 3 [file 44321_2025_354_MOESM4_ESM.zip › Fig 3/Fig 3D/western blot MDM2.jpg]

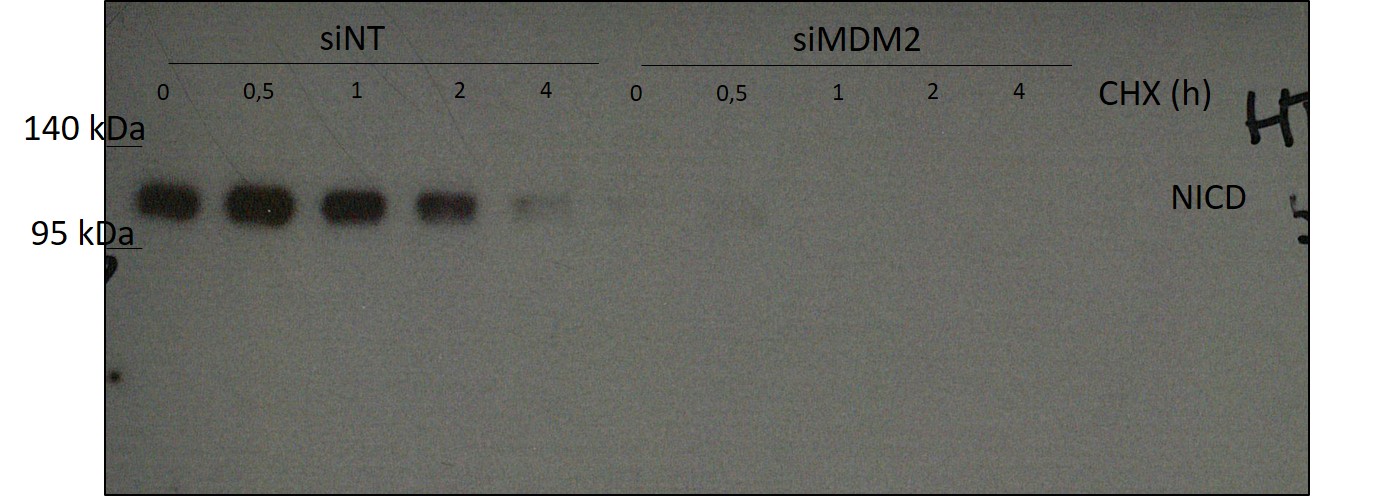

Supplement: Supplementary file 4 — Source data Fig. 3 [file 44321_2025_354_MOESM4_ESM.zip › Fig 3/Fig 3D/western blot nicd.jpg]

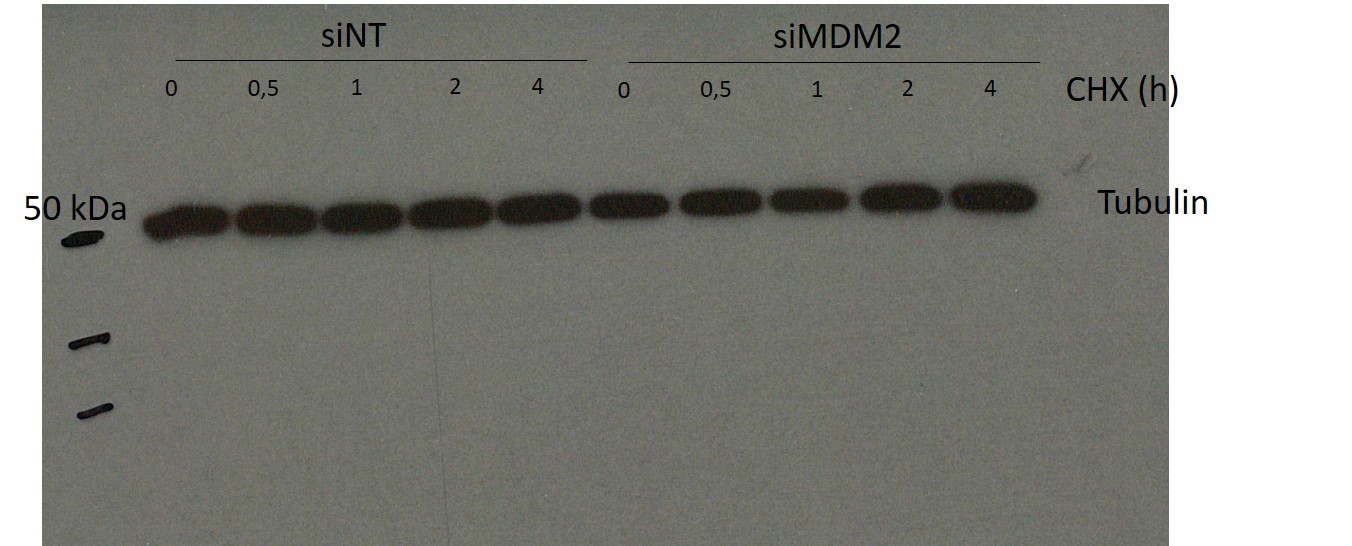

Supplement: Supplementary file 4 — Source data Fig. 3 [file 44321_2025_354_MOESM4_ESM.zip › Fig 3/Fig 3D/western blot tubulin.jpg]

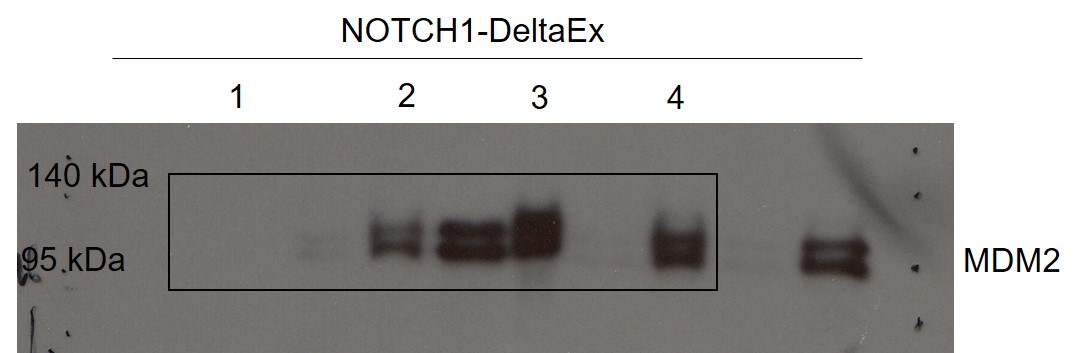

Supplement: Supplementary file 5 — Source data Fig. 4 [file 44321_2025_354_MOESM5_ESM.zip › Fig 4/Fig 4A/Fig4A replicat/WESTERN BLOT MDM2 N2.jpg]

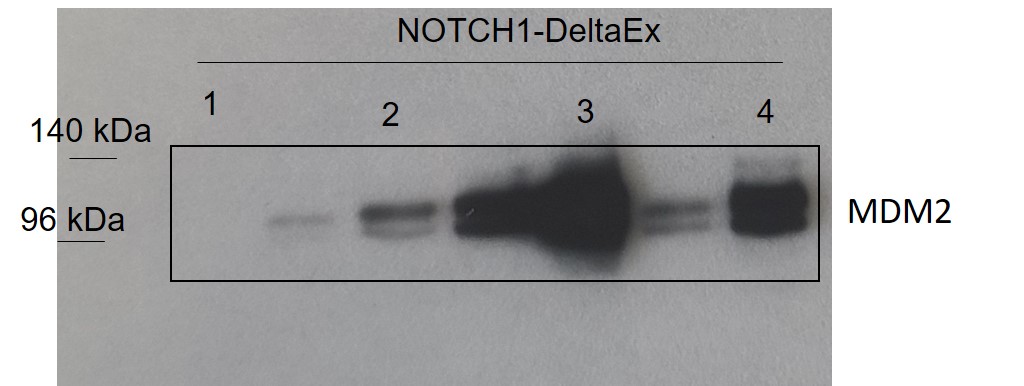

Supplement: Supplementary file 5 — Source data Fig. 4 [file 44321_2025_354_MOESM5_ESM.zip › Fig 4/Fig 4A/Fig4A replicat/WESTERN BLOT MDM2 N3.jpg]

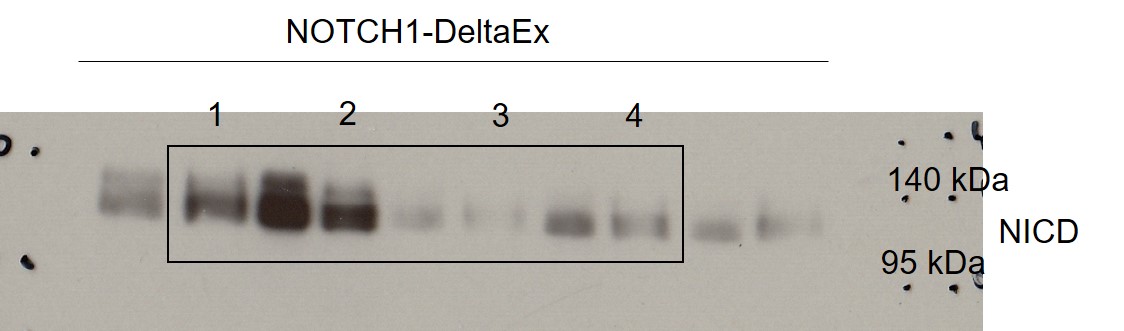

Supplement: Supplementary file 5 — Source data Fig. 4 [file 44321_2025_354_MOESM5_ESM.zip › Fig 4/Fig 4A/Fig4A replicat/WESTERN BLOT NICD N2.jpg]

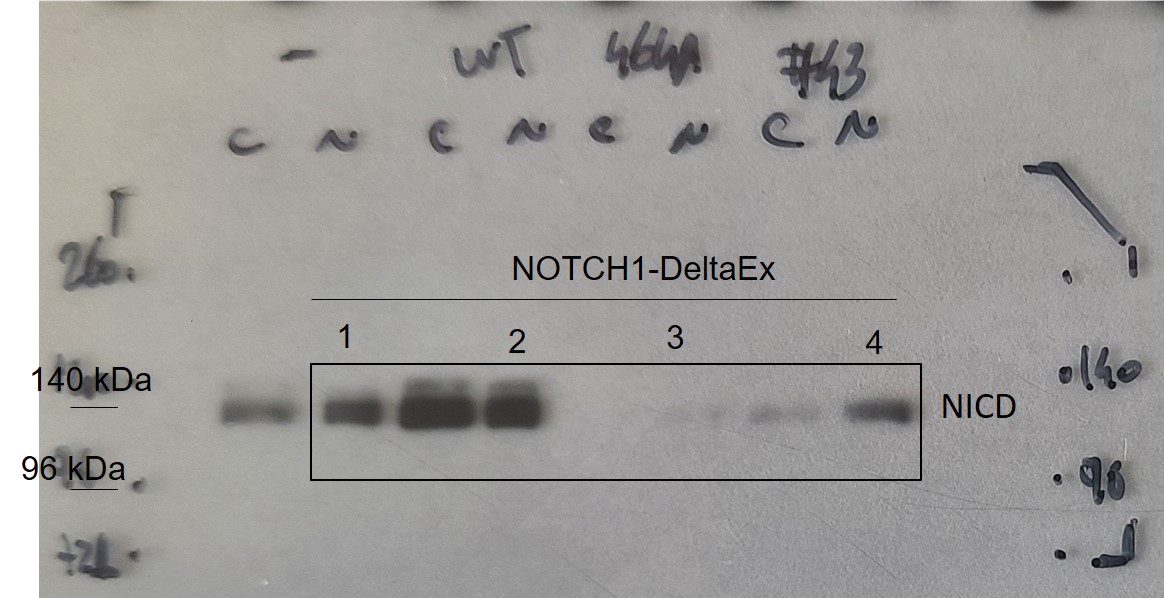

Supplement: Supplementary file 5 — Source data Fig. 4 [file 44321_2025_354_MOESM5_ESM.zip › Fig 4/Fig 4A/Fig4A replicat/WESTERN BLOT NICD N3.jpg]

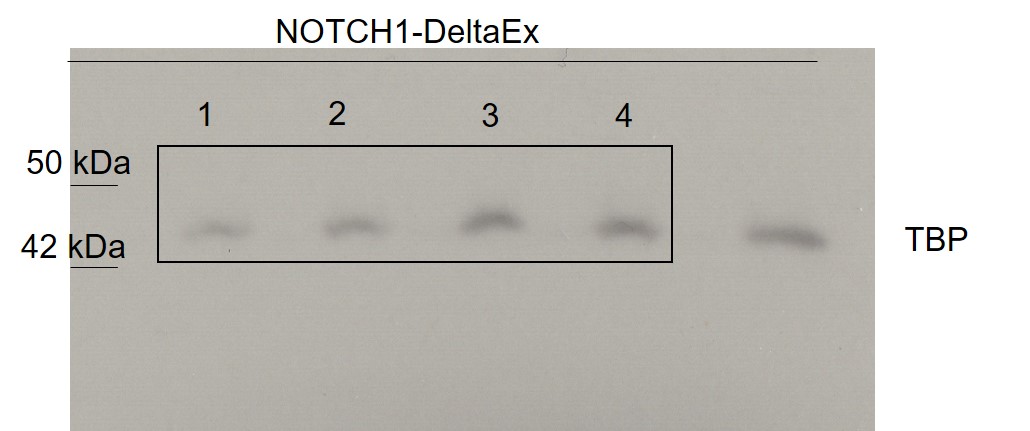

Supplement: Supplementary file 5 — Source data Fig. 4 [file 44321_2025_354_MOESM5_ESM.zip › Fig 4/Fig 4A/Fig4A replicat/WESTERN BLOT TBP N2.jpg]

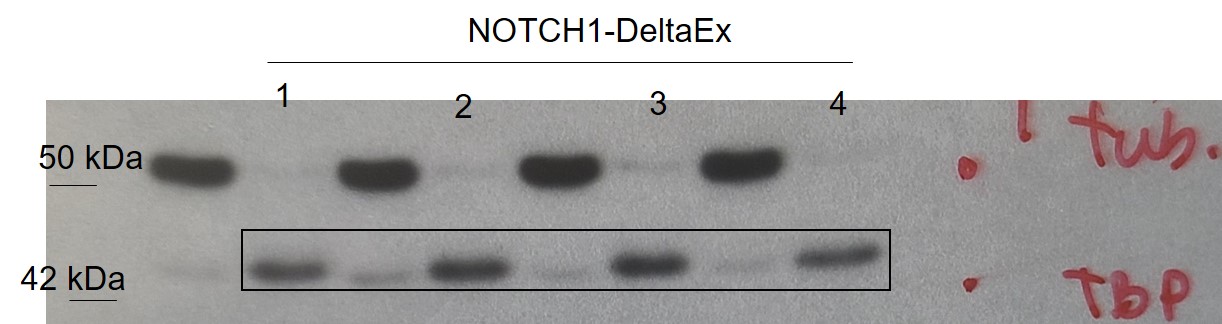

Supplement: Supplementary file 5 — Source data Fig. 4 [file 44321_2025_354_MOESM5_ESM.zip › Fig 4/Fig 4A/Fig4A replicat/WESTERN BLOT tbp N3.jpg]

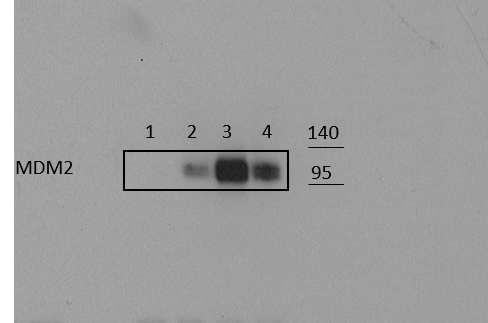

Supplement: Supplementary file 5 — Source data Fig. 4 [file 44321_2025_354_MOESM5_ESM.zip › Fig 4/Fig 4A/western blot MDM2.png]

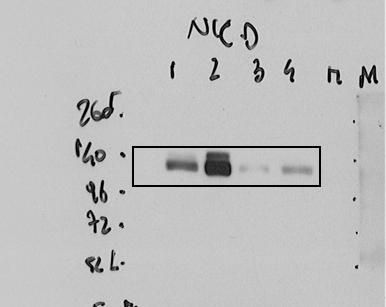

Supplement: Supplementary file 5 — Source data Fig. 4 [file 44321_2025_354_MOESM5_ESM.zip › Fig 4/Fig 4A/western blot NICD.png]

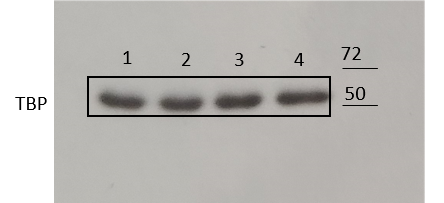

Supplement: Supplementary file 5 — Source data Fig. 4 [file 44321_2025_354_MOESM5_ESM.zip › Fig 4/Fig 4A/western blot TBP.png]

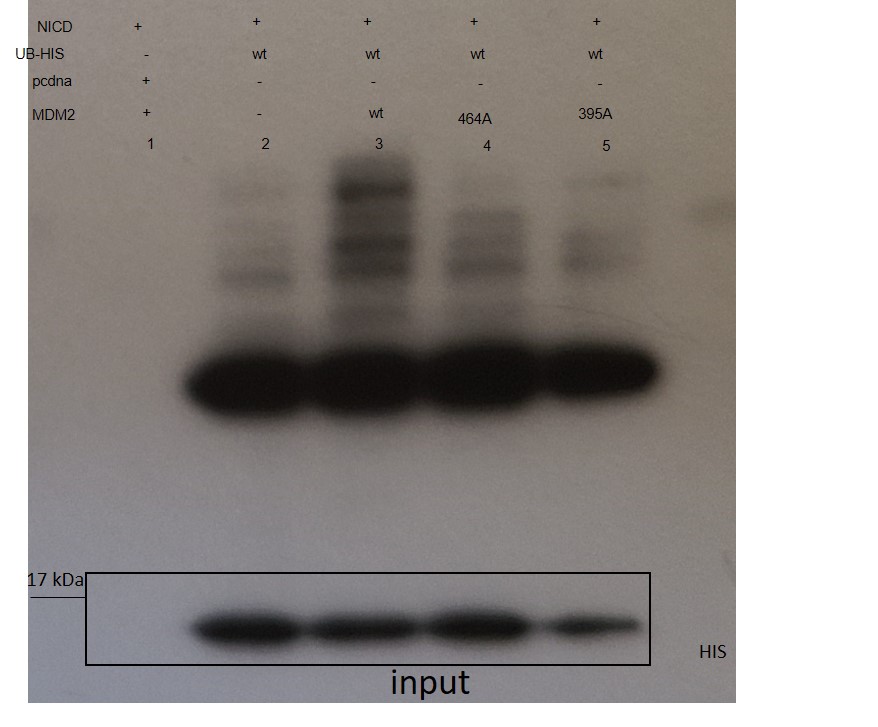

Supplement: Supplementary file 5 — Source data Fig. 4 [file 44321_2025_354_MOESM5_ESM.zip › Fig 4/Fig 4B/western blot HIS input.png.jpg]

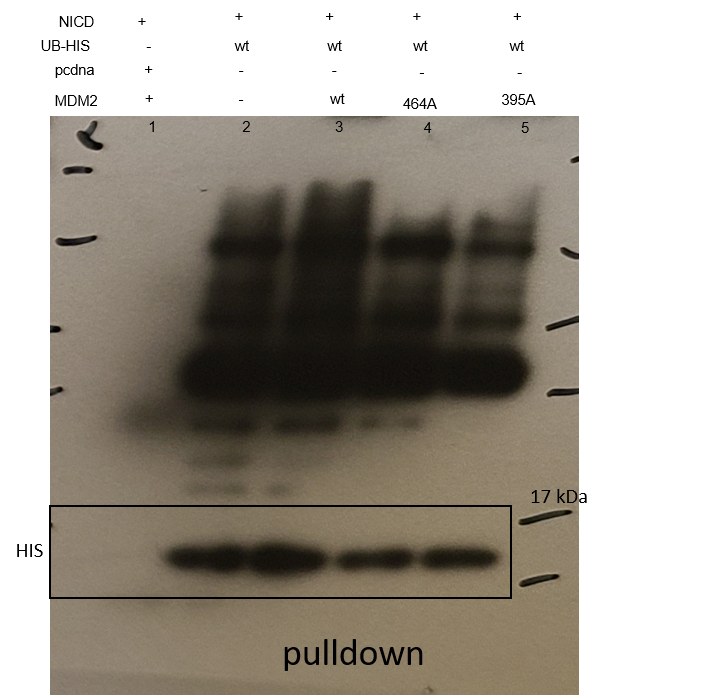

Supplement: Supplementary file 5 — Source data Fig. 4 [file 44321_2025_354_MOESM5_ESM.zip › Fig 4/Fig 4B/western blot HIS pulldown.png]
