# Supplementary figures and images for: NOTCH1 intracellular domain stabilization by MDM2 plays a major role in NSCLC response to platinum (part 2 of 3)
Source: EMBO Mol Med. 2026 Jan 16;18(2):514–41. doi: 10.1038/s44321-025-00354-9 (PMC12905330; doi:10.1038/s44321-025-00354-9)

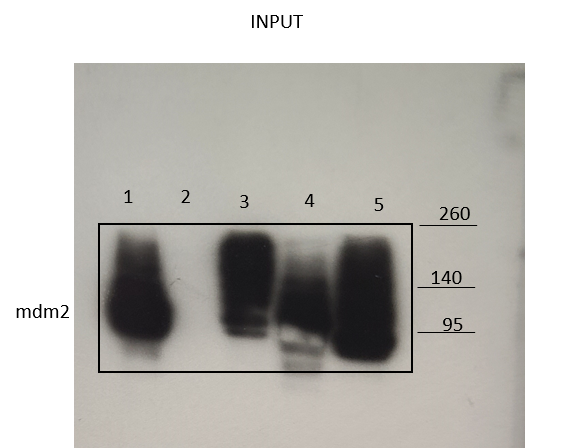

Supplement: Supplementary file 5 — Source data Fig. 4 [file 44321_2025_354_MOESM5_ESM.zip › Fig 4/Fig 4B/western blot MDM2 input.png]

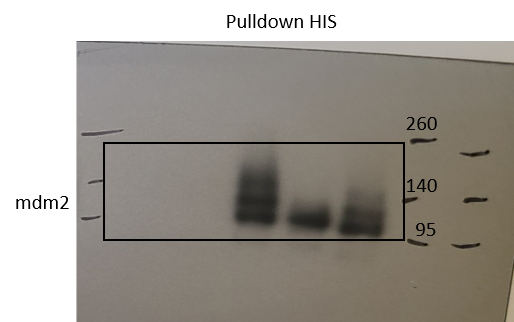

Supplement: Supplementary file 5 — Source data Fig. 4 [file 44321_2025_354_MOESM5_ESM.zip › Fig 4/Fig 4B/western blot MDM2 PULLDOWN.png]

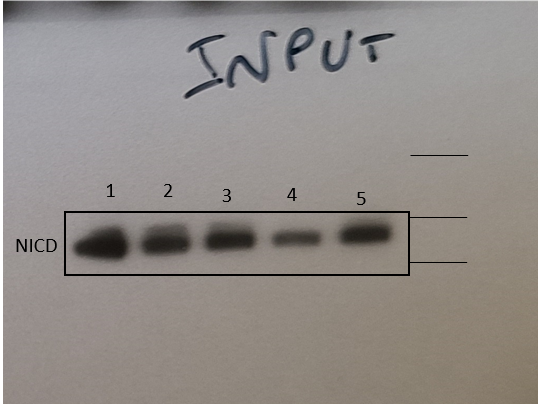

Supplement: Supplementary file 5 — Source data Fig. 4 [file 44321_2025_354_MOESM5_ESM.zip › Fig 4/Fig 4B/western blot NICD input.png]

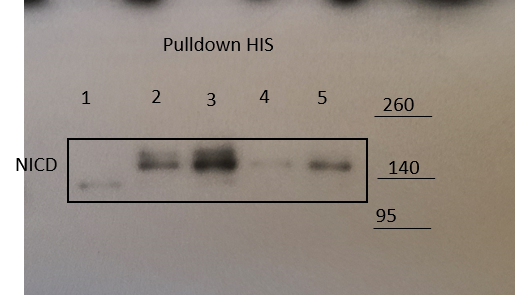

Supplement: Supplementary file 5 — Source data Fig. 4 [file 44321_2025_354_MOESM5_ESM.zip › Fig 4/Fig 4B/western blot NICD PULLDOWN.png]

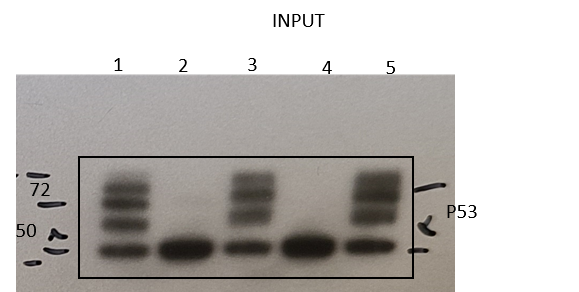

Supplement: Supplementary file 5 — Source data Fig. 4 [file 44321_2025_354_MOESM5_ESM.zip › Fig 4/Fig 4B/western blot P53 input.png]

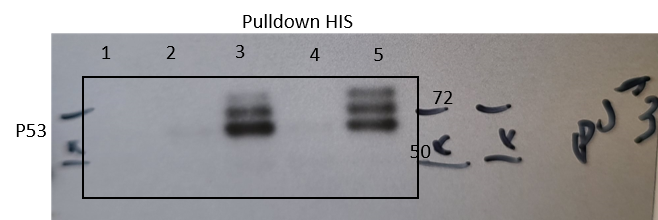

Supplement: Supplementary file 5 — Source data Fig. 4 [file 44321_2025_354_MOESM5_ESM.zip › Fig 4/Fig 4B/western blot P53 PULLDOWN.png]

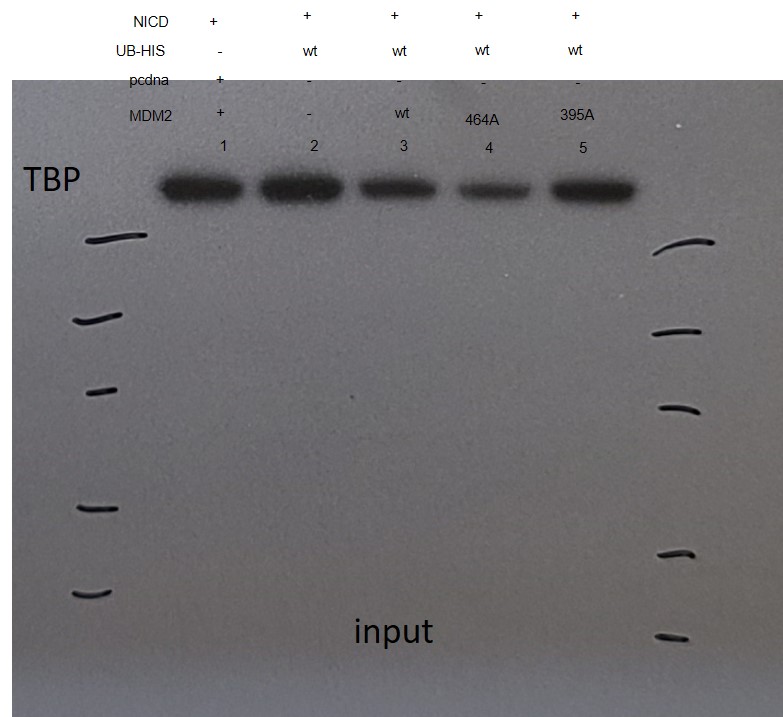

Supplement: Supplementary file 5 — Source data Fig. 4 [file 44321_2025_354_MOESM5_ESM.zip › Fig 4/Fig 4B/western blot TBP input.jpg]

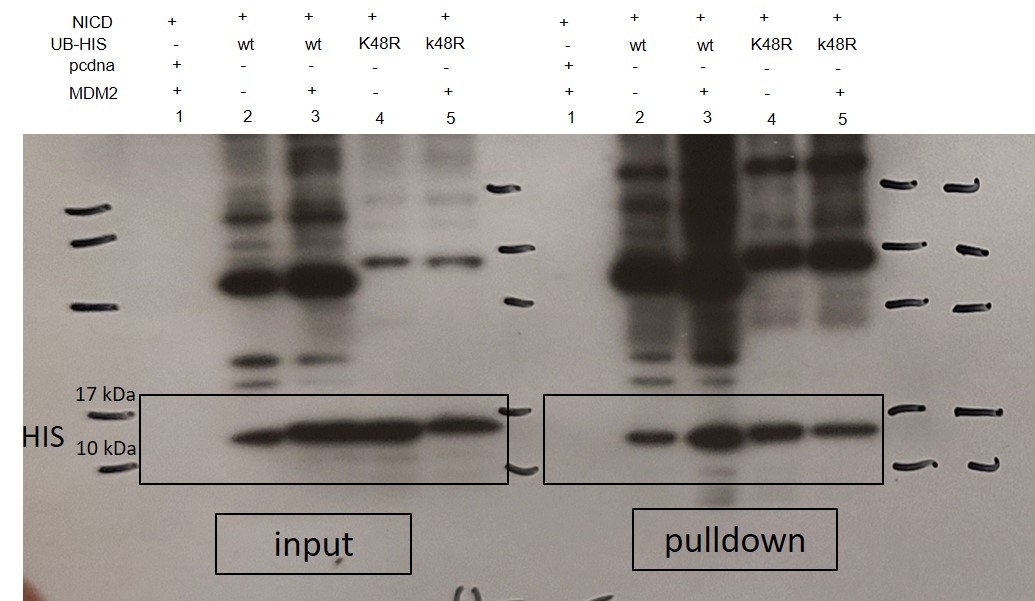

Supplement: Supplementary file 5 — Source data Fig. 4 [file 44321_2025_354_MOESM5_ESM.zip › Fig 4/Fig 4C/Fig 4C REPLICAT/western blot HIS Input, pulldown N2.jpg]

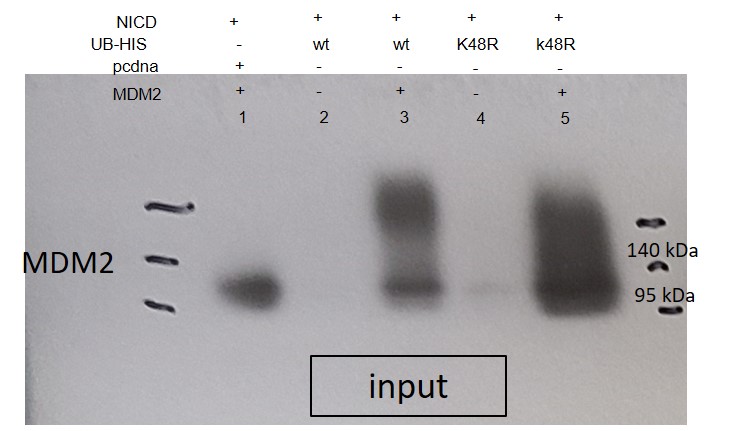

Supplement: Supplementary file 5 — Source data Fig. 4 [file 44321_2025_354_MOESM5_ESM.zip › Fig 4/Fig 4C/Fig 4C REPLICAT/western blot MDM2 Input N2.jpg]

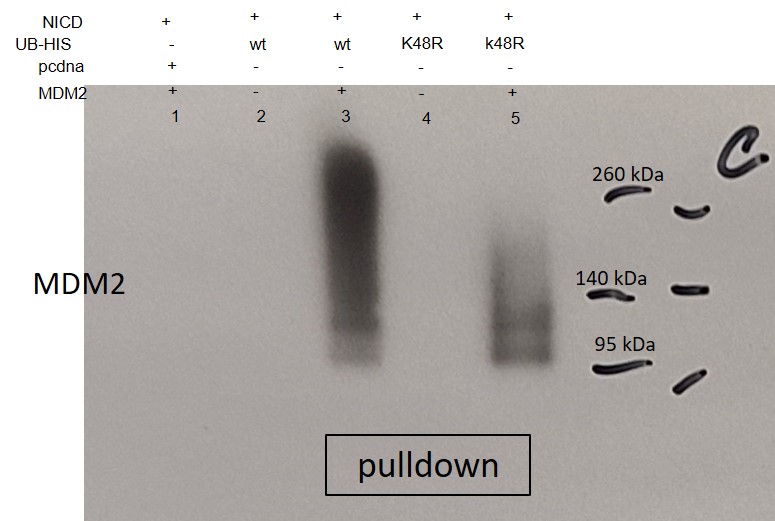

Supplement: Supplementary file 5 — Source data Fig. 4 [file 44321_2025_354_MOESM5_ESM.zip › Fig 4/Fig 4C/Fig 4C REPLICAT/western blot MDM2 pulldown N2.jpg]

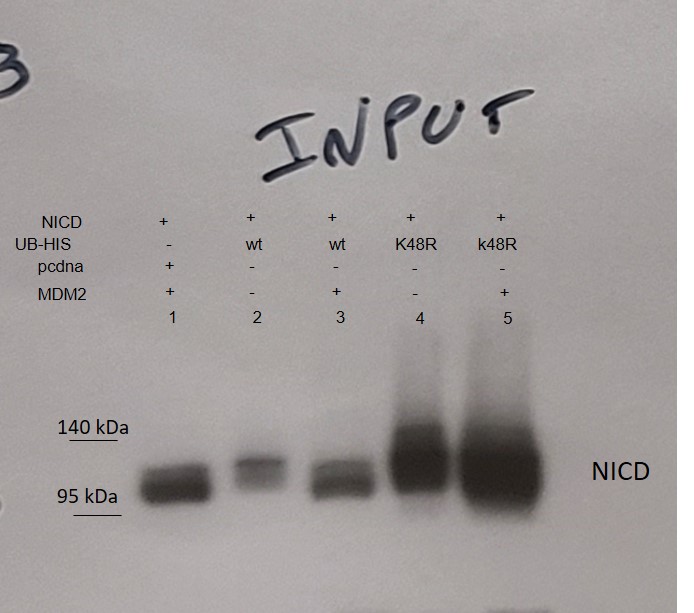

Supplement: Supplementary file 5 — Source data Fig. 4 [file 44321_2025_354_MOESM5_ESM.zip › Fig 4/Fig 4C/Fig 4C REPLICAT/western blot NICD Input N2.jpg]

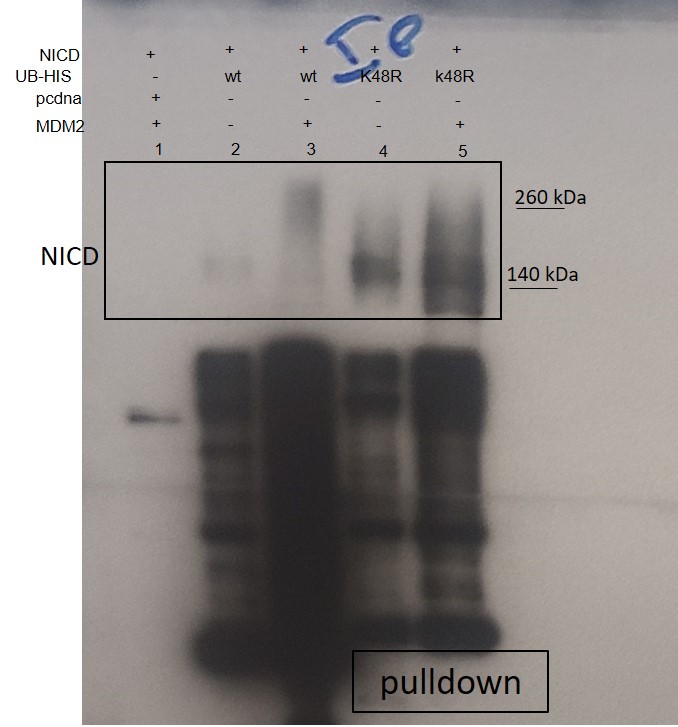

Supplement: Supplementary file 5 — Source data Fig. 4 [file 44321_2025_354_MOESM5_ESM.zip › Fig 4/Fig 4C/Fig 4C REPLICAT/western blot NICD pulldown N2.jpg]

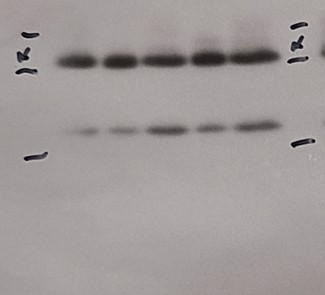

Supplement: Supplementary file 5 — Source data Fig. 4 [file 44321_2025_354_MOESM5_ESM.zip › Fig 4/Fig 4C/Fig 4C REPLICAT/western blot TBP Input N2.jpg]

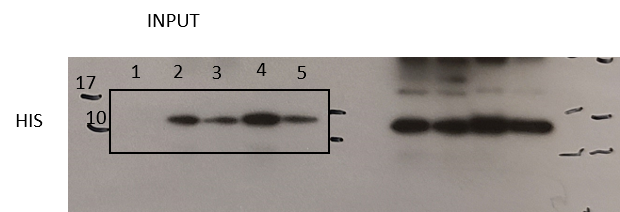

Supplement: Supplementary file 5 — Source data Fig. 4 [file 44321_2025_354_MOESM5_ESM.zip › Fig 4/Fig 4C/western blot HIS input.png]

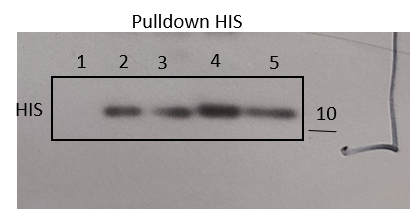

Supplement: Supplementary file 5 — Source data Fig. 4 [file 44321_2025_354_MOESM5_ESM.zip › Fig 4/Fig 4C/western blot HIS pulldown.png]

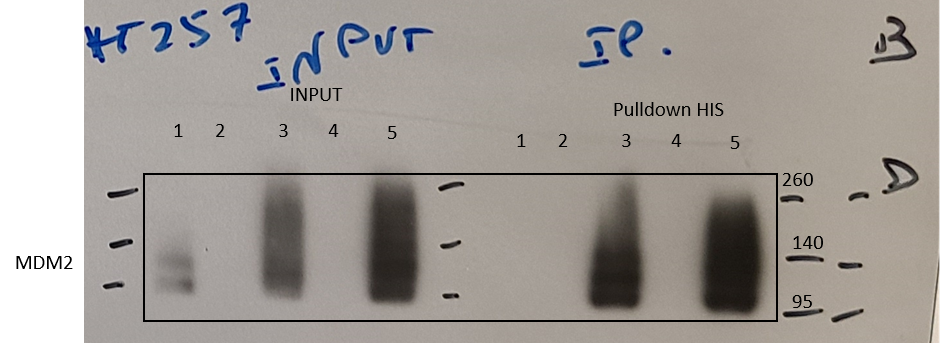

Supplement: Supplementary file 5 — Source data Fig. 4 [file 44321_2025_354_MOESM5_ESM.zip › Fig 4/Fig 4C/western blot MDM2 input and pulldown.png]

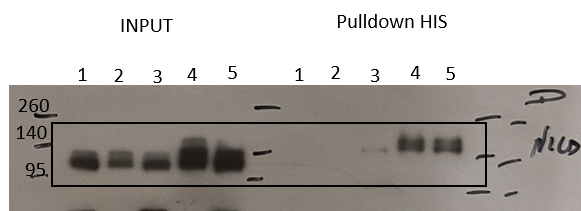

Supplement: Supplementary file 5 — Source data Fig. 4 [file 44321_2025_354_MOESM5_ESM.zip › Fig 4/Fig 4C/western blot NICD input and pulldown.png]

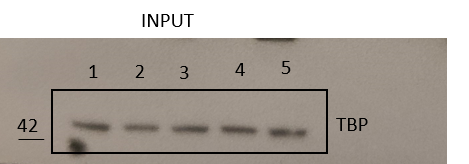

Supplement: Supplementary file 5 — Source data Fig. 4 [file 44321_2025_354_MOESM5_ESM.zip › Fig 4/Fig 4C/western blot TBP input.png]

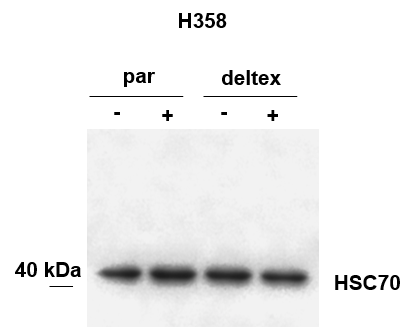

Supplement: Supplementary file 6 — Source data Fig. 5 [file 44321_2025_354_MOESM6_ESM.zip › Fig 5/Fig 5A/western blot HSC70.png]

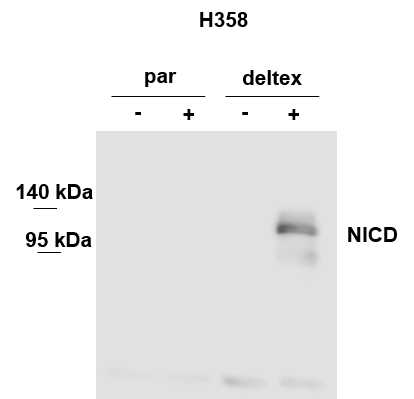

Supplement: Supplementary file 6 — Source data Fig. 5 [file 44321_2025_354_MOESM6_ESM.zip › Fig 5/Fig 5A/western blot NICD.png]

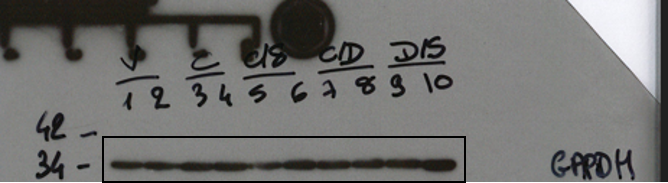

Supplement: Supplementary file 6 — Source data Fig. 5 [file 44321_2025_354_MOESM6_ESM.zip › Fig 5/Fig 5E/western blot GAPDH.png]

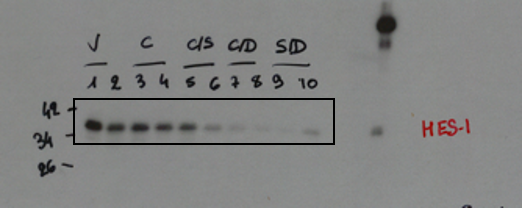

Supplement: Supplementary file 6 — Source data Fig. 5 [file 44321_2025_354_MOESM6_ESM.zip › Fig 5/Fig 5E/western blot HES1.png]

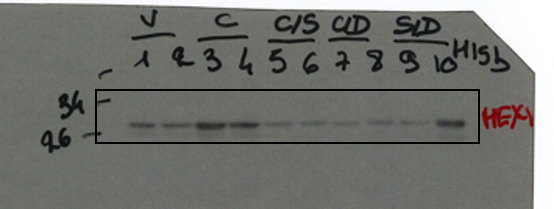

Supplement: Supplementary file 6 — Source data Fig. 5 [file 44321_2025_354_MOESM6_ESM.zip › Fig 5/Fig 5E/western blot HEY1.png]

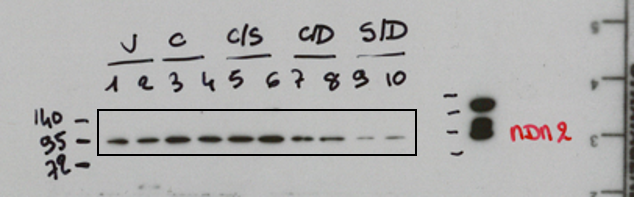

Supplement: Supplementary file 6 — Source data Fig. 5 [file 44321_2025_354_MOESM6_ESM.zip › Fig 5/Fig 5E/western blot MDM2.png]

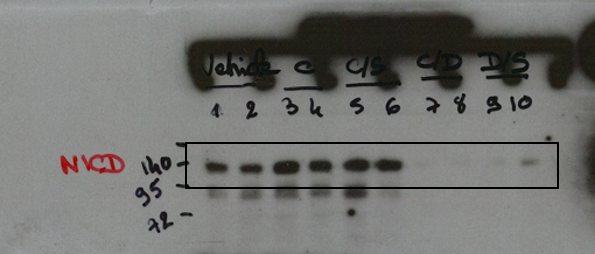

Supplement: Supplementary file 6 — Source data Fig. 5 [file 44321_2025_354_MOESM6_ESM.zip › Fig 5/Fig 5E/western blot NICD.png]

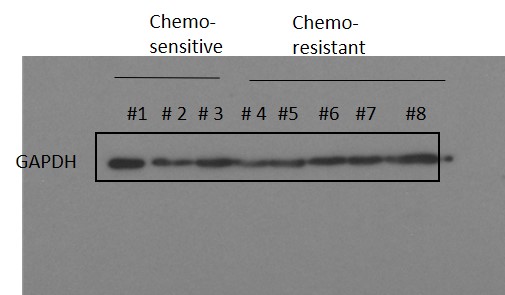

Supplement: Supplementary file 7 — Source data Fig. 6 [file 44321_2025_354_MOESM7_ESM.zip › Fig 6/Fig 6B/western blot gapdh.jpg]

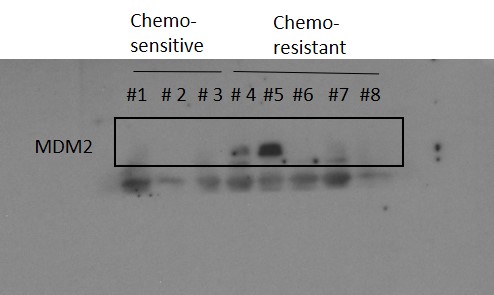

Supplement: Supplementary file 7 — Source data Fig. 6 [file 44321_2025_354_MOESM7_ESM.zip › Fig 6/Fig 6B/western blot mdm2.jpg]

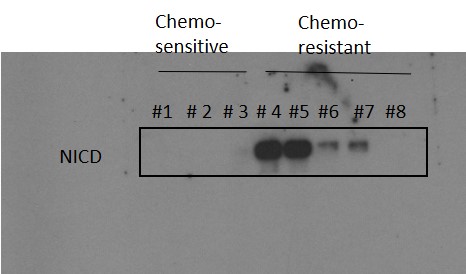

Supplement: Supplementary file 7 — Source data Fig. 6 [file 44321_2025_354_MOESM7_ESM.zip › Fig 6/Fig 6B/western blot nicd.jpg]

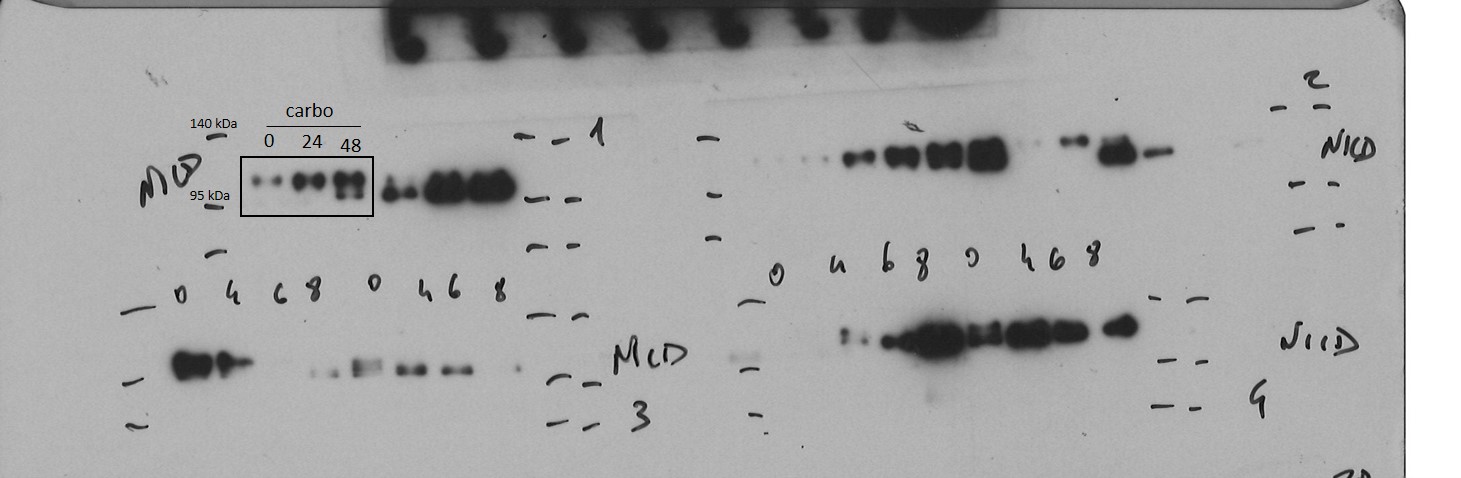

Supplement: Supplementary file 8 — Figure EV1 Source Data [file 44321_2025_354_MOESM8_ESM.zip › Fig EV1/Fig EV1/Fig EV1A/replicat/western blot NICD carbo N2.jpg]

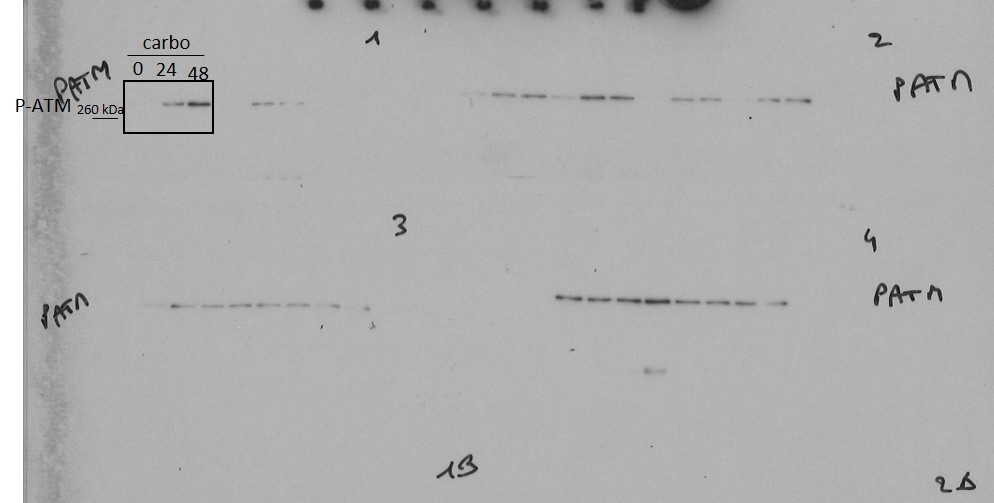

Supplement: Supplementary file 8 — Figure EV1 Source Data [file 44321_2025_354_MOESM8_ESM.zip › Fig EV1/Fig EV1/Fig EV1A/replicat/western blot P-ATM carbo N2.jpg]

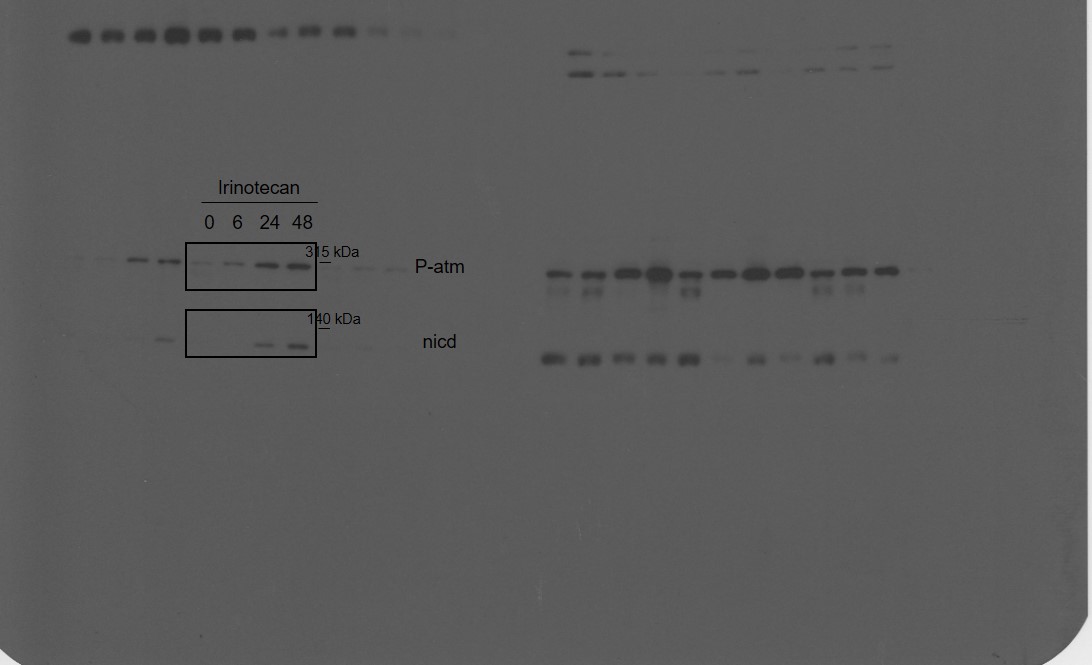

Supplement: Supplementary file 8 — Figure EV1 Source Data [file 44321_2025_354_MOESM8_ESM.zip › Fig EV1/Fig EV1/Fig EV1A/replicat/western blot P-ATM, NICD irinotecan N2.jpg]

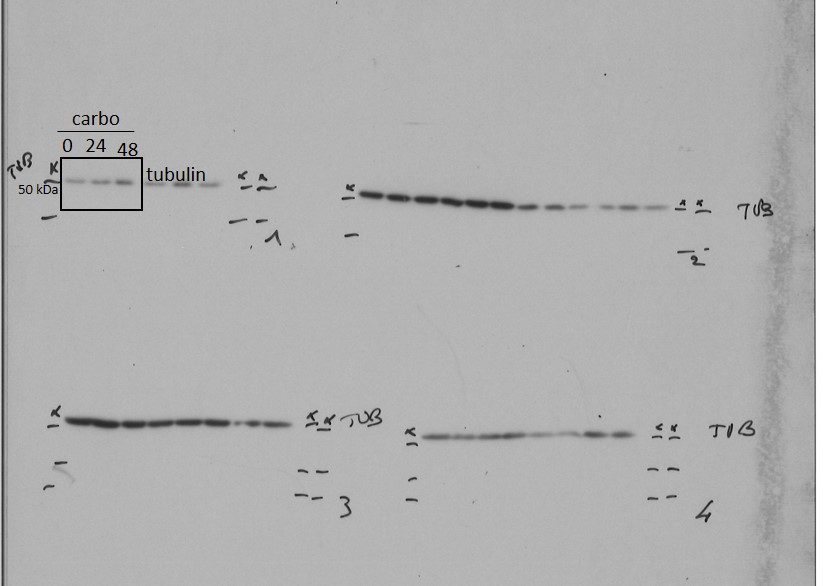

Supplement: Supplementary file 8 — Figure EV1 Source Data [file 44321_2025_354_MOESM8_ESM.zip › Fig EV1/Fig EV1/Fig EV1A/replicat/western blot tubulin carbo N2.jpg]

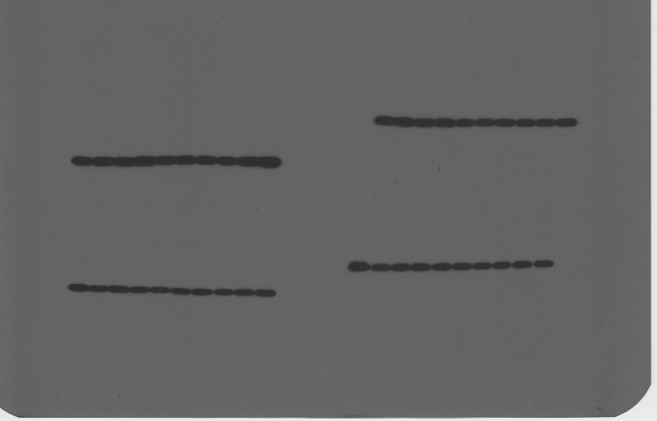

Supplement: Supplementary file 8 — Figure EV1 Source Data [file 44321_2025_354_MOESM8_ESM.zip › Fig EV1/Fig EV1/Fig EV1A/replicat/western blot tubulin irinotecan N2.jpg]

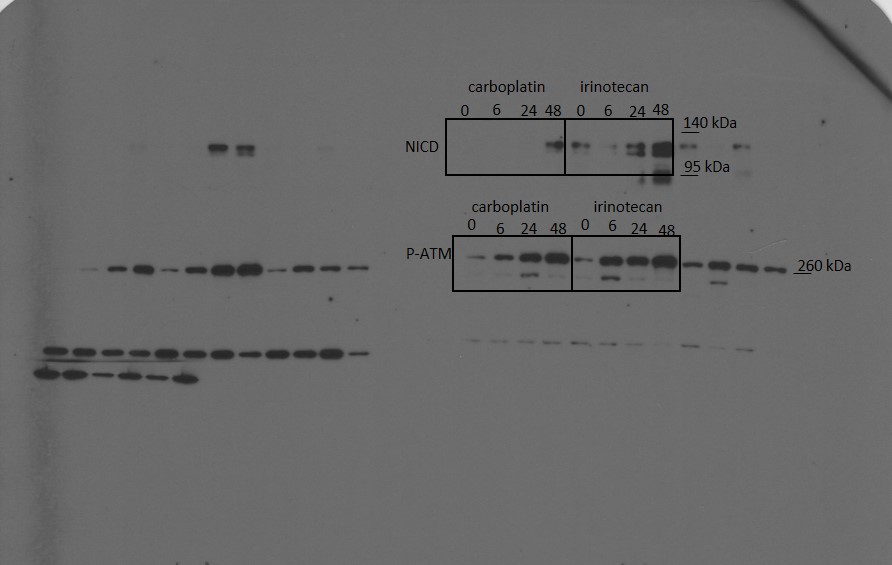

Supplement: Supplementary file 8 — Figure EV1 Source Data [file 44321_2025_354_MOESM8_ESM.zip › Fig EV1/Fig EV1/Fig EV1A/western blot NICD, P-ATM carbo, irinotecan.jpg]

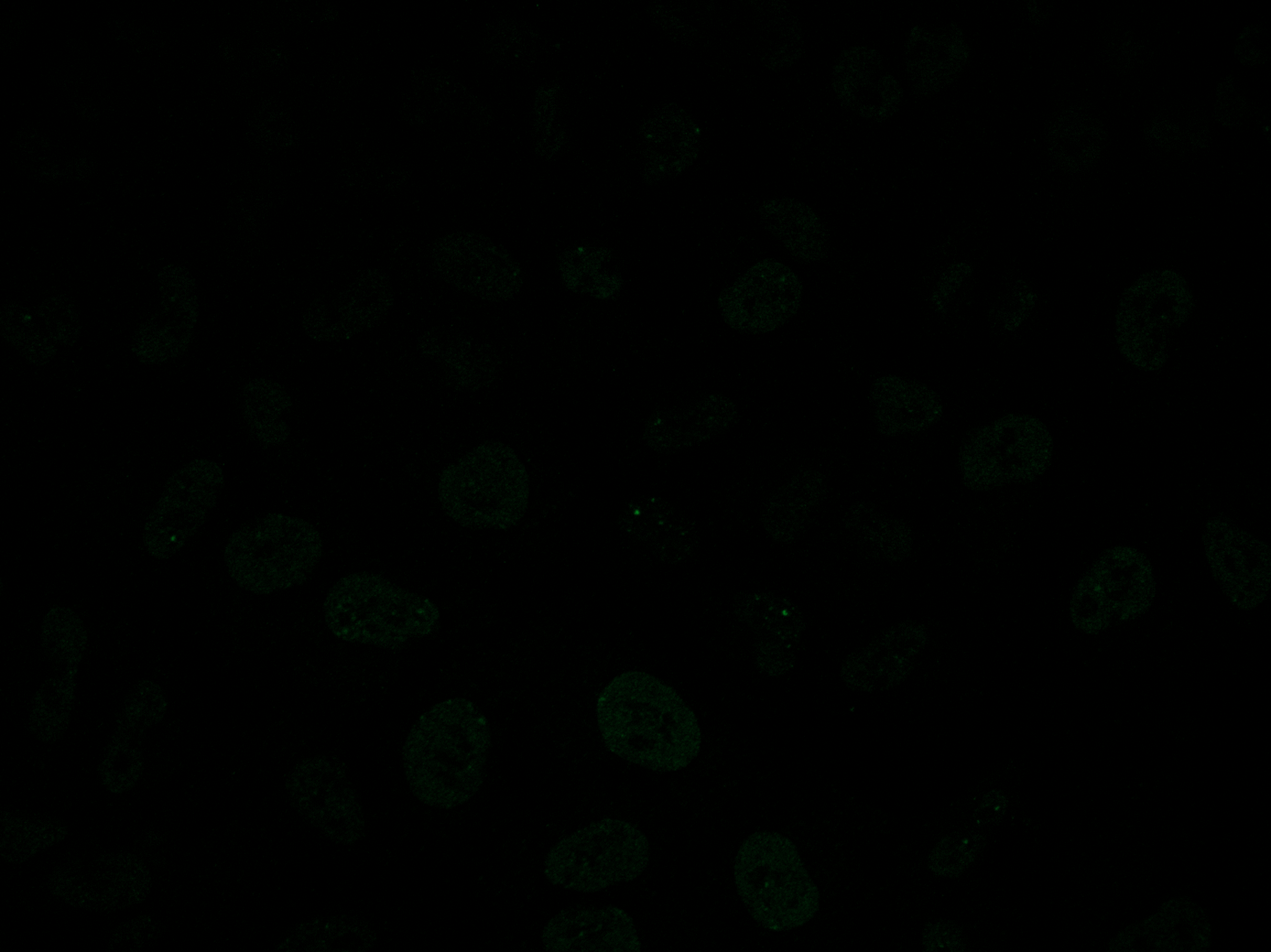

Supplement: Supplementary file 8 — Figure EV1 Source Data [file 44321_2025_354_MOESM8_ESM.zip › Fig EV1/Fig EV1/Fig EV1B/0/IP-ApoTome-44_53bp1.tif]

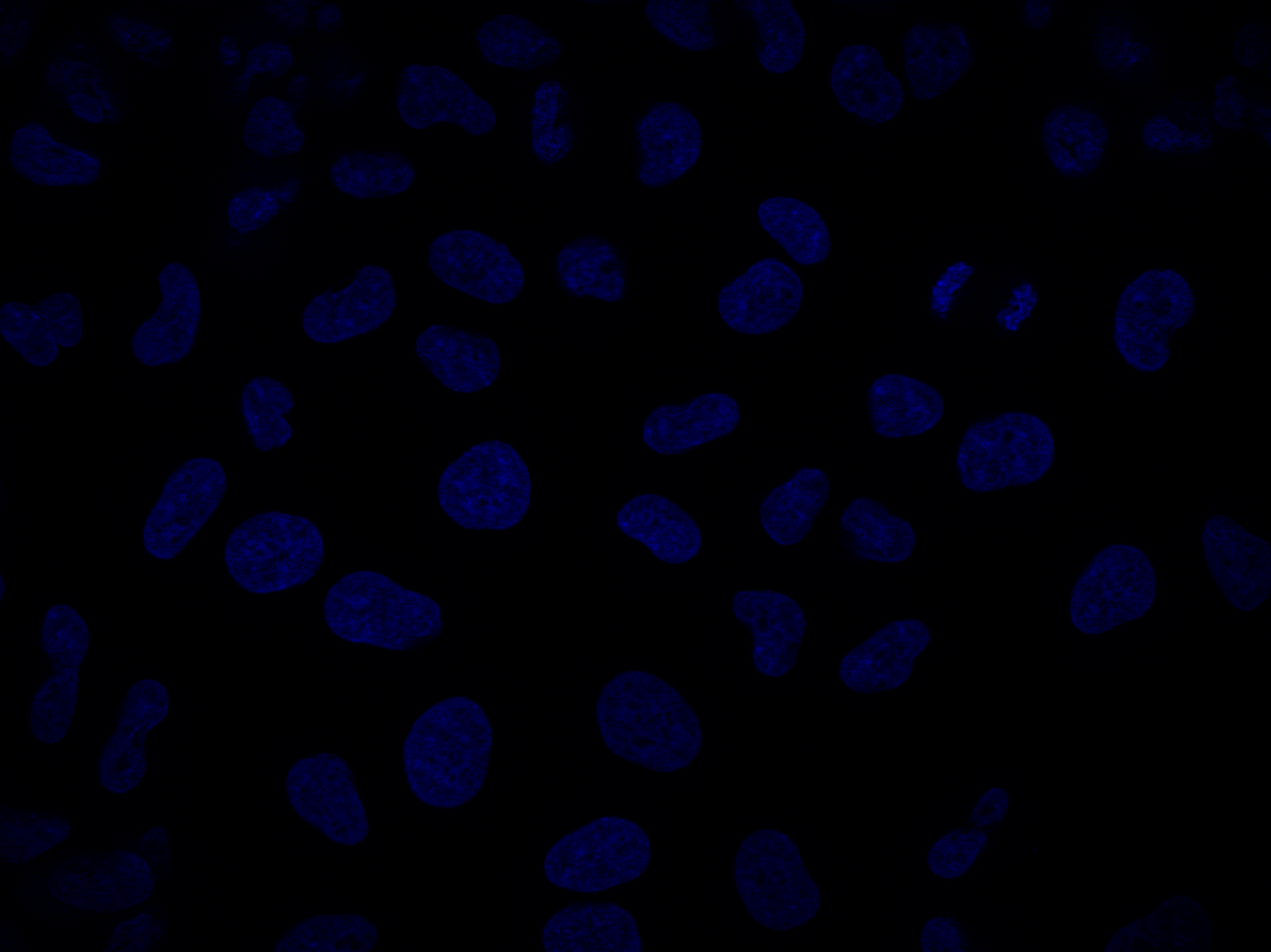

Supplement: Supplementary file 8 — Figure EV1 Source Data [file 44321_2025_354_MOESM8_ESM.zip › Fig EV1/Fig EV1/Fig EV1B/0/IP-ApoTome-44_dap1.tif]

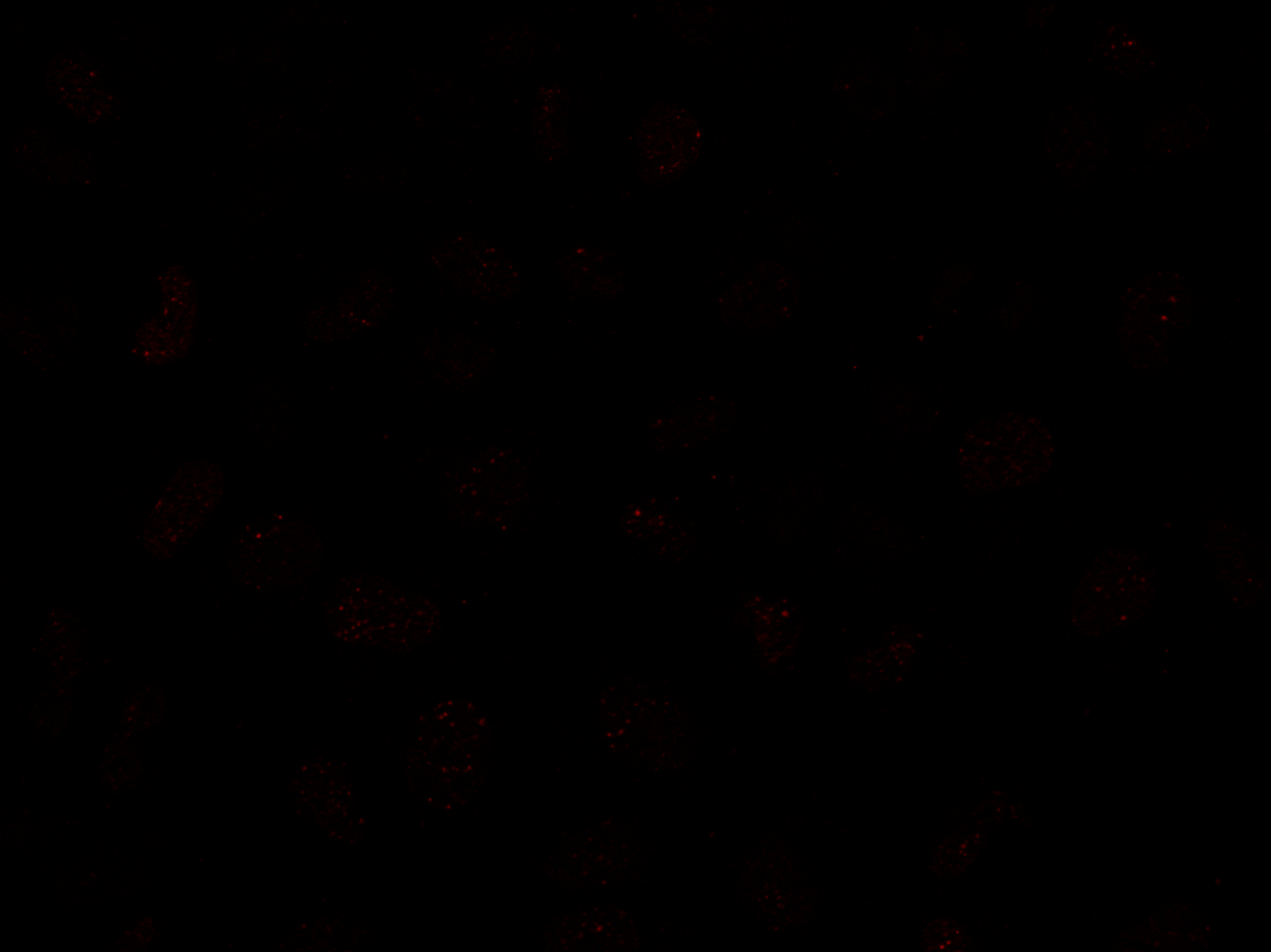

Supplement: Supplementary file 8 — Figure EV1 Source Data [file 44321_2025_354_MOESM8_ESM.zip › Fig EV1/Fig EV1/Fig EV1B/0/IP-ApoTome-44_h2ax.tif]

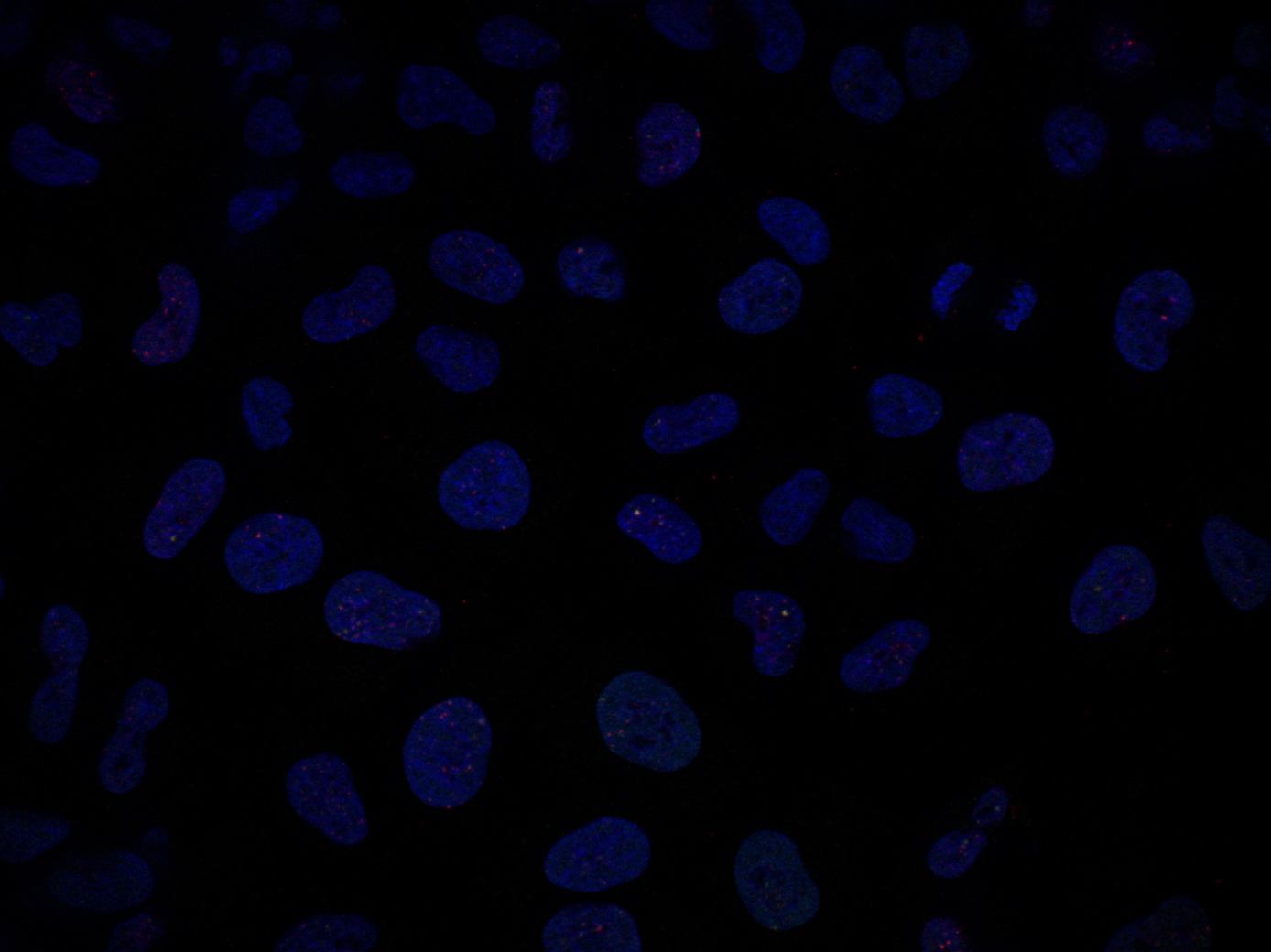

Supplement: Supplementary file 8 — Figure EV1 Source Data [file 44321_2025_354_MOESM8_ESM.zip › Fig EV1/Fig EV1/Fig EV1B/0/IP-ApoTome-44_merge.tif]

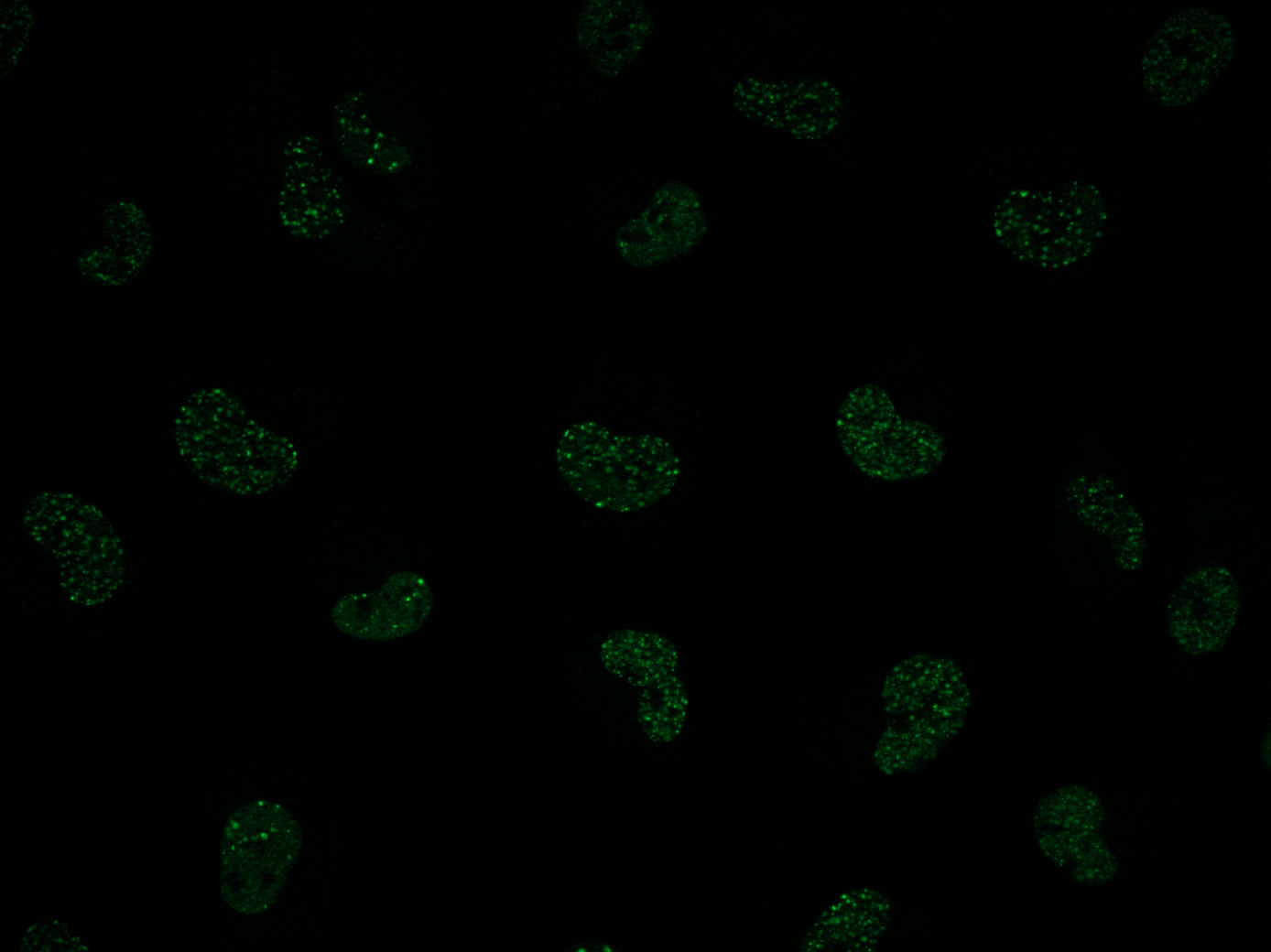

Supplement: Supplementary file 8 — Figure EV1 Source Data [file 44321_2025_354_MOESM8_ESM.zip › Fig EV1/Fig EV1/Fig EV1B/24/IP-ApoTome-38_53bp1.tif]

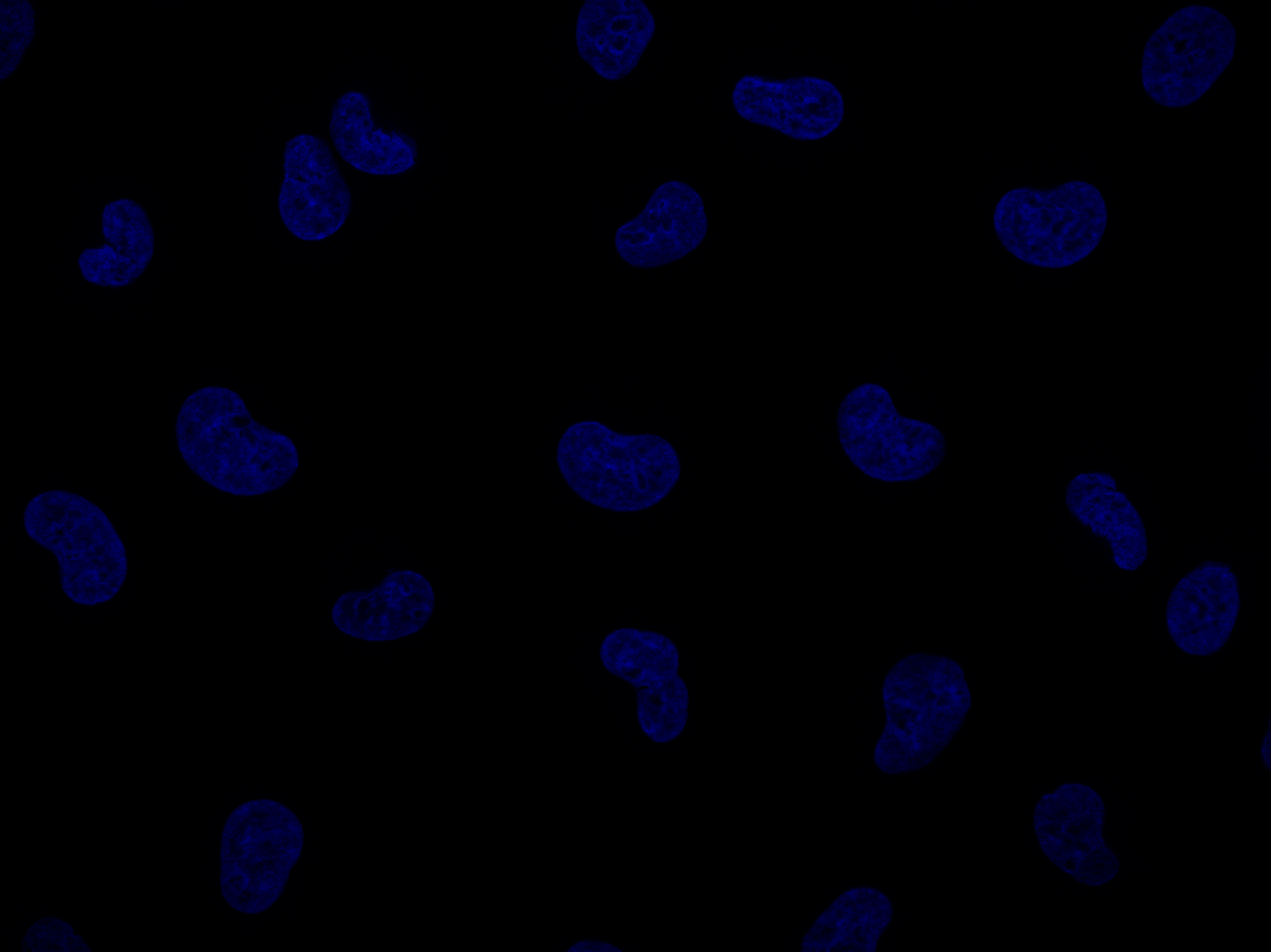

Supplement: Supplementary file 8 — Figure EV1 Source Data [file 44321_2025_354_MOESM8_ESM.zip › Fig EV1/Fig EV1/Fig EV1B/24/IP-ApoTome-38_DAPI.tif]

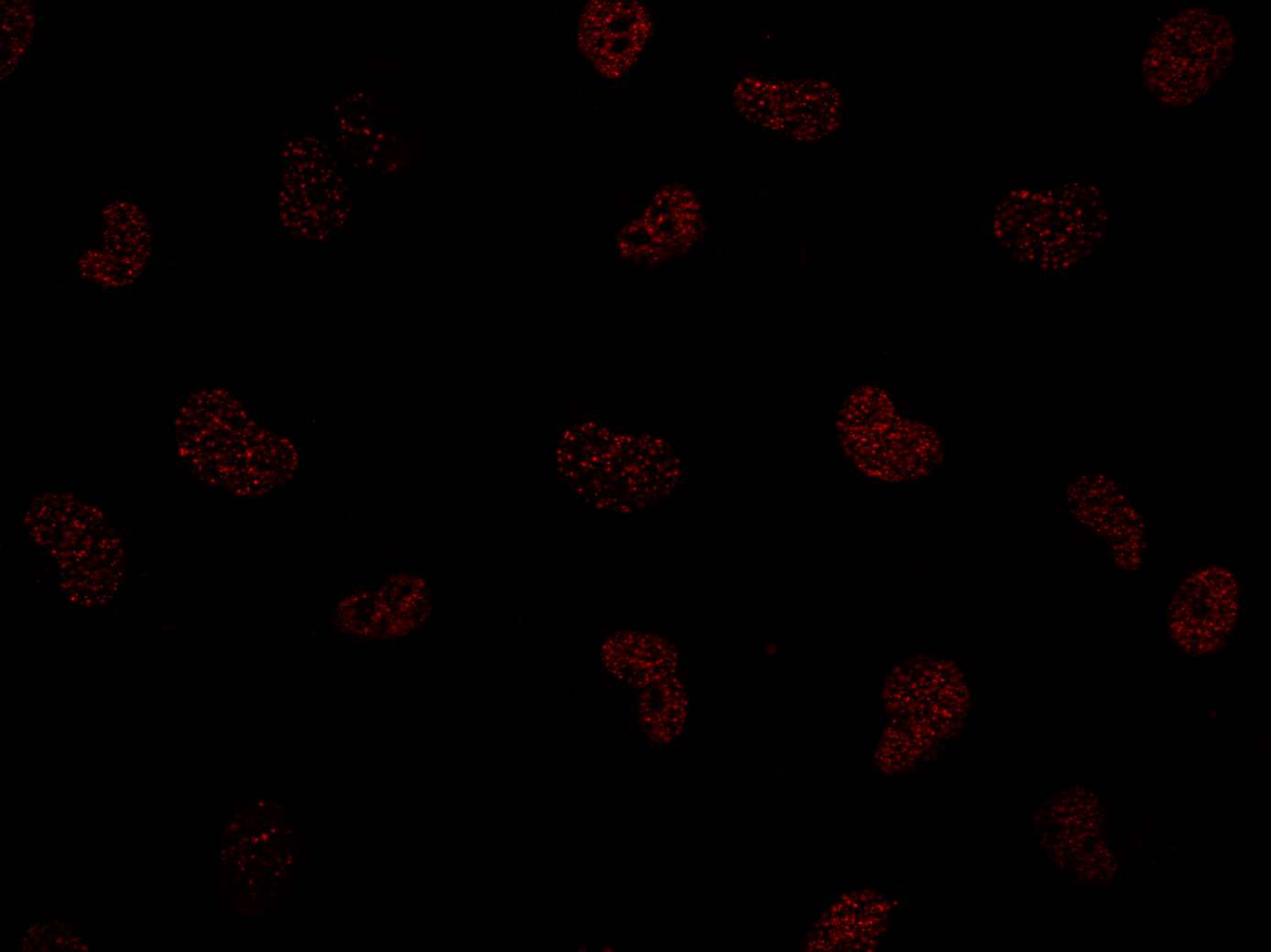

Supplement: Supplementary file 8 — Figure EV1 Source Data [file 44321_2025_354_MOESM8_ESM.zip › Fig EV1/Fig EV1/Fig EV1B/24/IP-ApoTome-38_h2ax.tif]

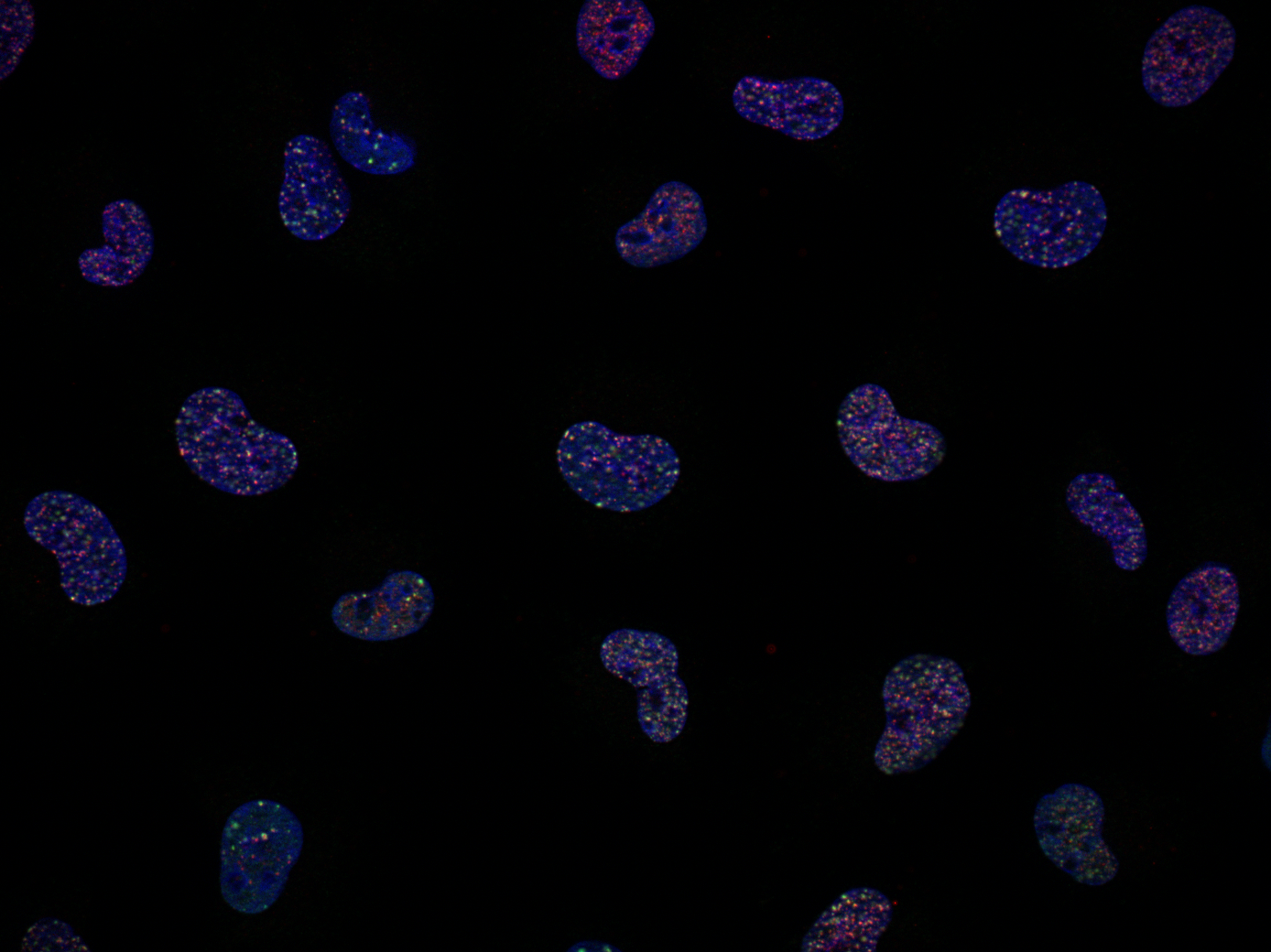

Supplement: Supplementary file 8 — Figure EV1 Source Data [file 44321_2025_354_MOESM8_ESM.zip › Fig EV1/Fig EV1/Fig EV1B/24/IP-ApoTome-38_merge.tif]

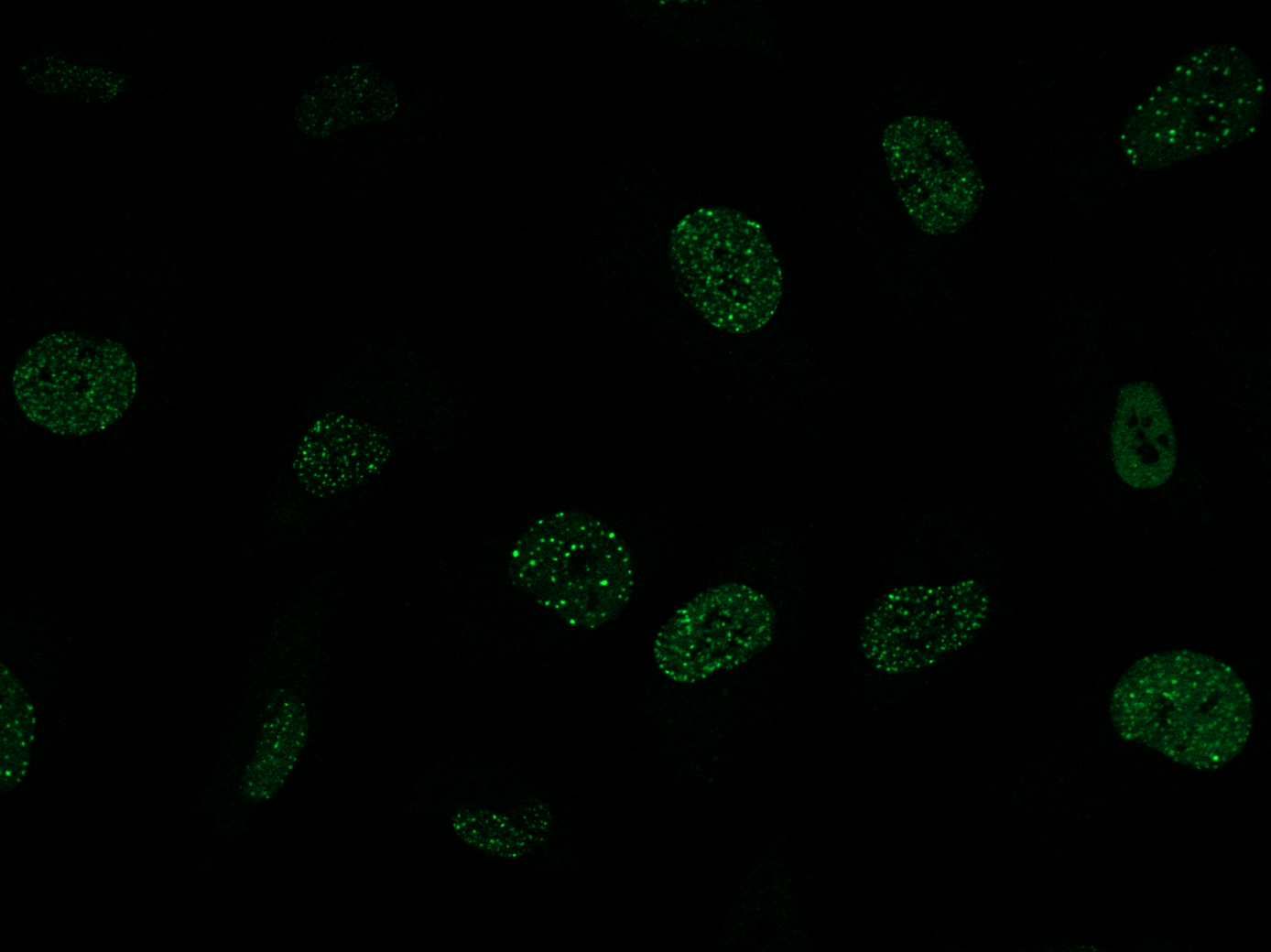

Supplement: Supplementary file 8 — Figure EV1 Source Data [file 44321_2025_354_MOESM8_ESM.zip › Fig EV1/Fig EV1/Fig EV1B/48/IP-ApoTome-33_53BP1.tif]

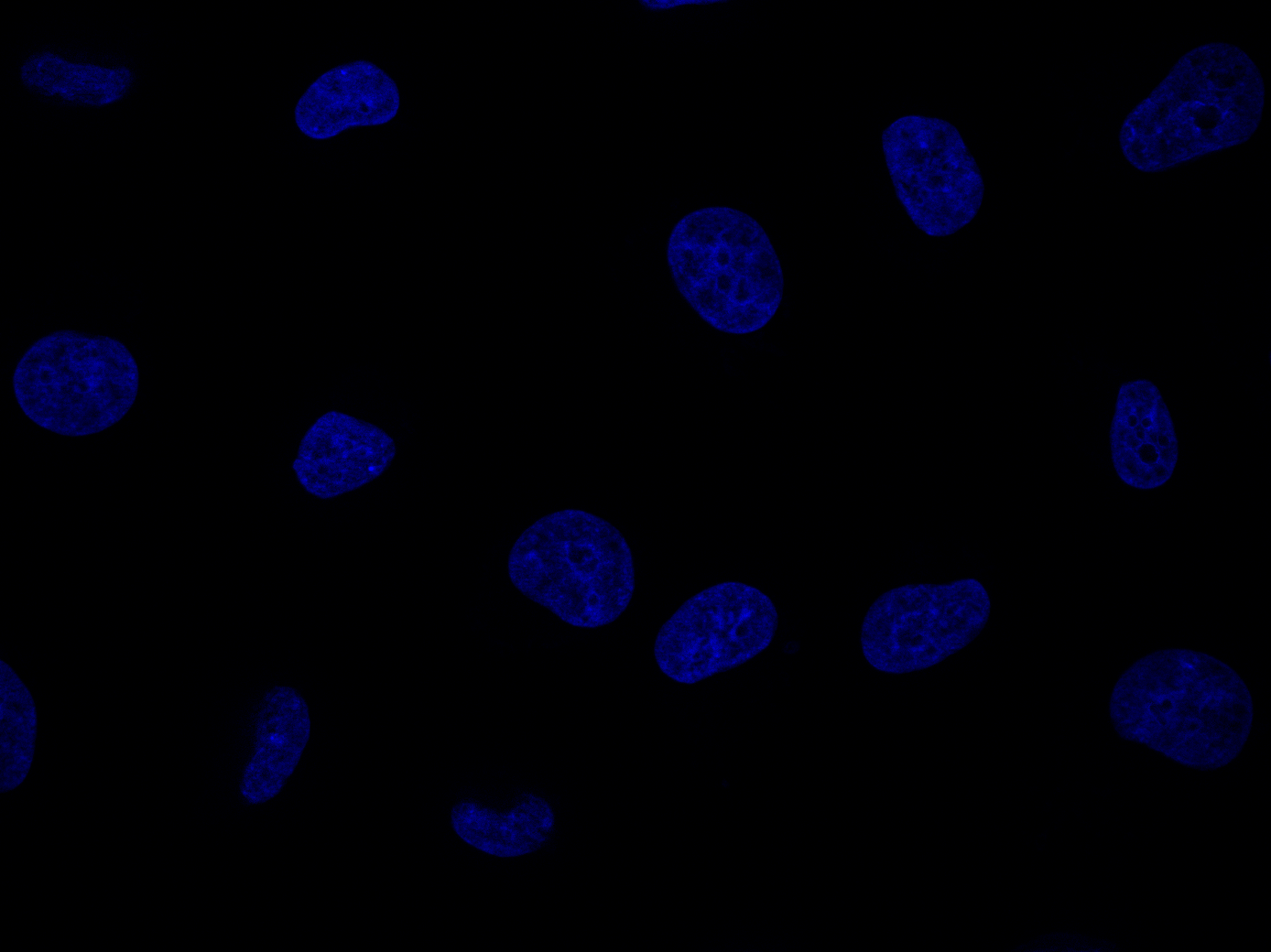

Supplement: Supplementary file 8 — Figure EV1 Source Data [file 44321_2025_354_MOESM8_ESM.zip › Fig EV1/Fig EV1/Fig EV1B/48/IP-ApoTome-33_DAPI.tif]

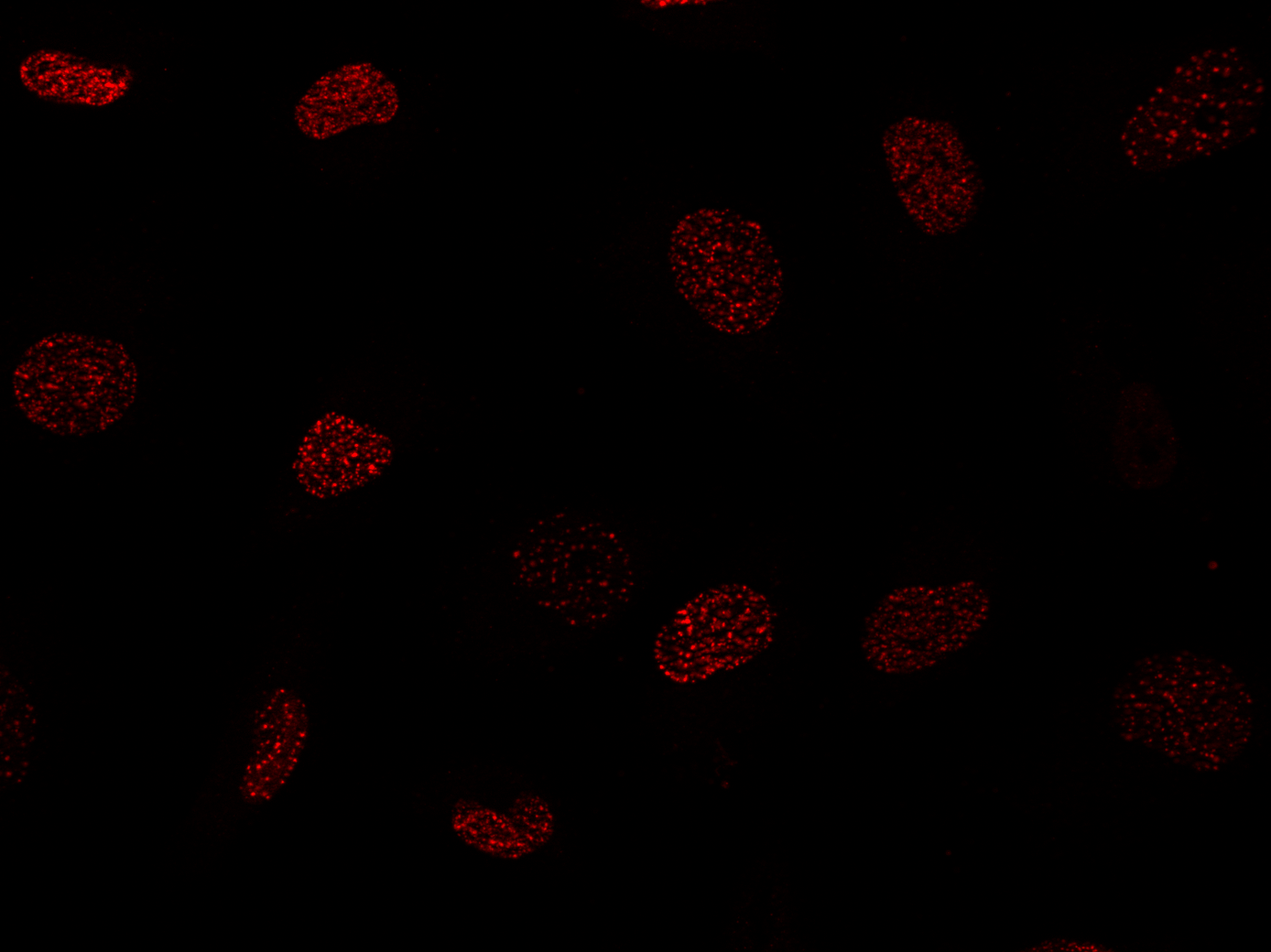

Supplement: Supplementary file 8 — Figure EV1 Source Data [file 44321_2025_354_MOESM8_ESM.zip › Fig EV1/Fig EV1/Fig EV1B/48/IP-ApoTome-33_H2AX.tif]

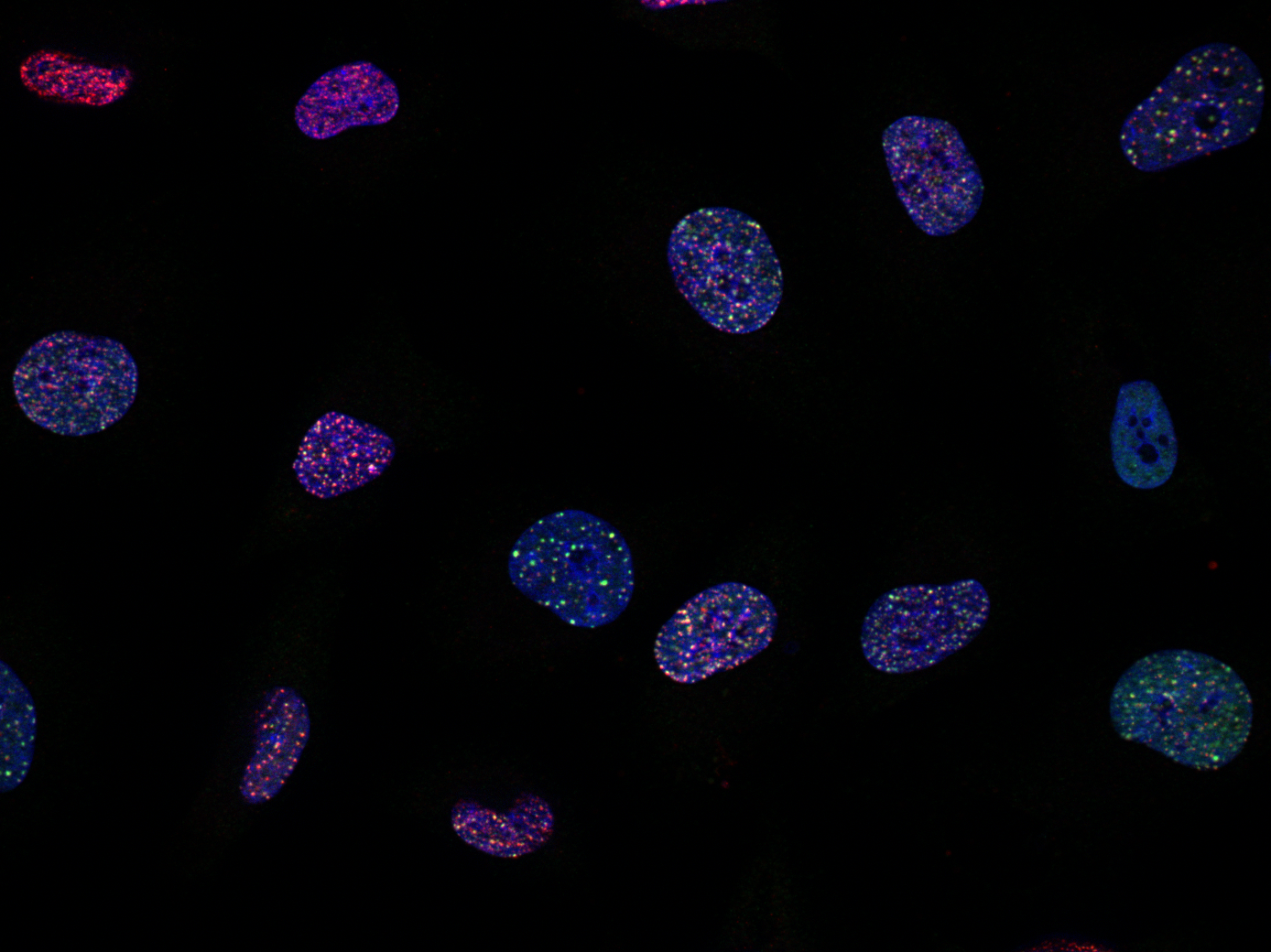

Supplement: Supplementary file 8 — Figure EV1 Source Data [file 44321_2025_354_MOESM8_ESM.zip › Fig EV1/Fig EV1/Fig EV1B/48/IP-ApoTome-33_MERGE.tif]

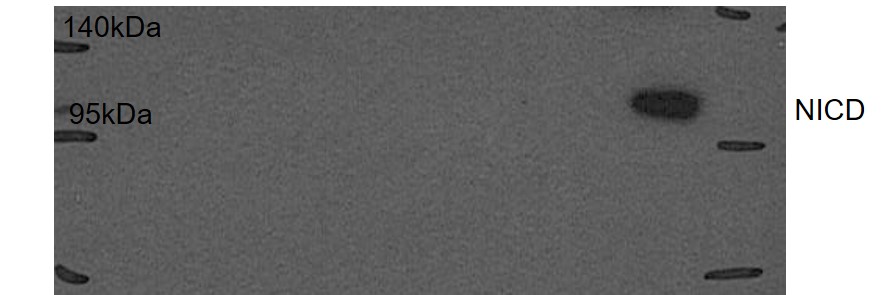

Supplement: Supplementary file 8 — Figure EV1 Source Data [file 44321_2025_354_MOESM8_ESM.zip › Fig EV1/Fig EV1/Fig EV1C/FigEV1C replicate/western blot nicd N2.jpg]

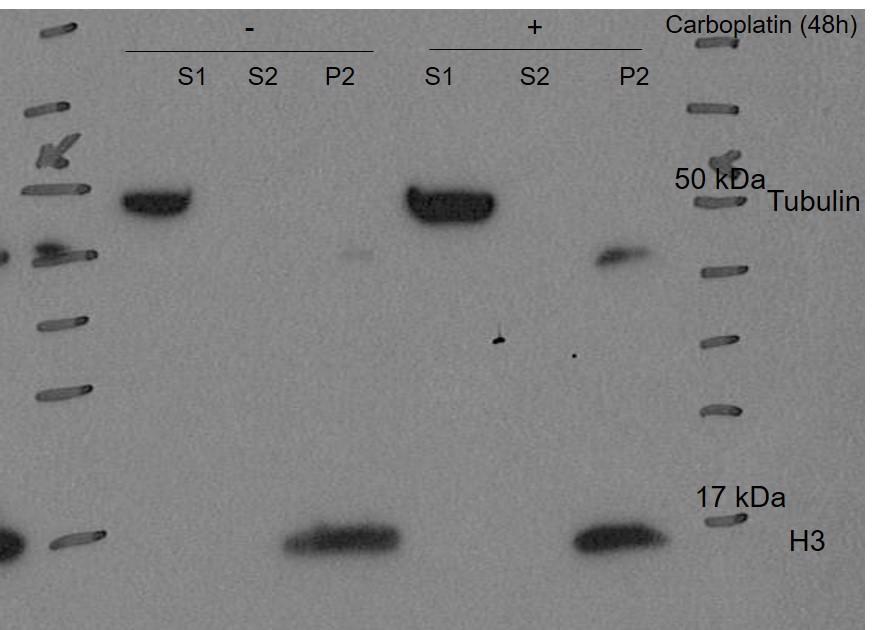

Supplement: Supplementary file 8 — Figure EV1 Source Data [file 44321_2025_354_MOESM8_ESM.zip › Fig EV1/Fig EV1/Fig EV1C/FigEV1C replicate/western blot tubulin, H3 N2.jpg]

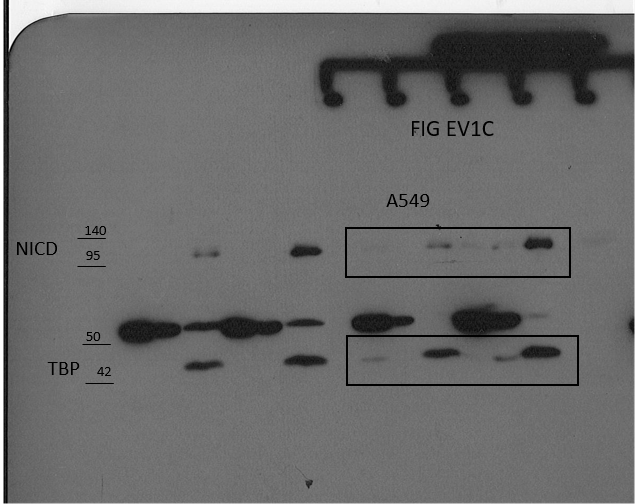

Supplement: Supplementary file 8 — Figure EV1 Source Data [file 44321_2025_354_MOESM8_ESM.zip › Fig EV1/Fig EV1/Fig EV1C/WESTERN BLOT NICD, TBP.png]

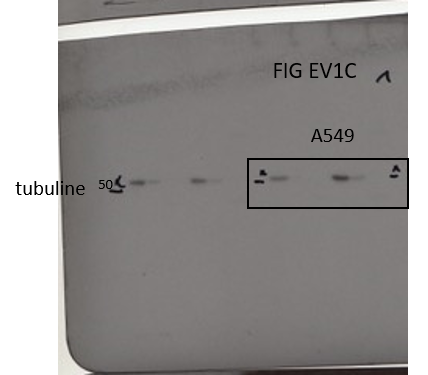

Supplement: Supplementary file 8 — Figure EV1 Source Data [file 44321_2025_354_MOESM8_ESM.zip › Fig EV1/Fig EV1/Fig EV1C/WESTERN BLOT TUBULIN.png]

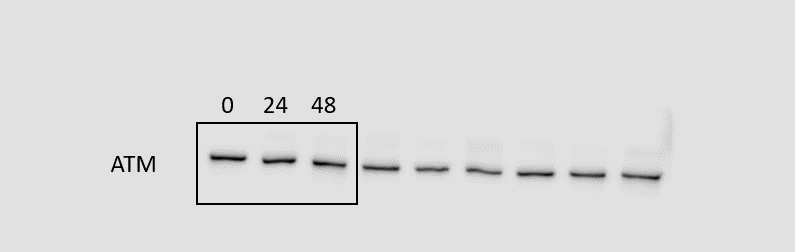

Supplement: Supplementary file 8 — Figure EV1 Source Data [file 44321_2025_354_MOESM8_ESM.zip › Fig EV1/Fig EV1/Fig EV1D/ATM WB #4.png]

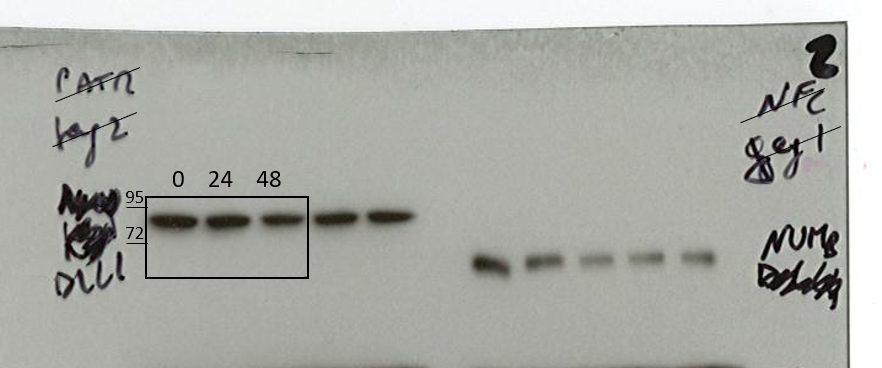

Supplement: Supplementary file 8 — Figure EV1 Source Data [file 44321_2025_354_MOESM8_ESM.zip › Fig EV1/Fig EV1/Fig EV1D/DLL1, wb#2.png]

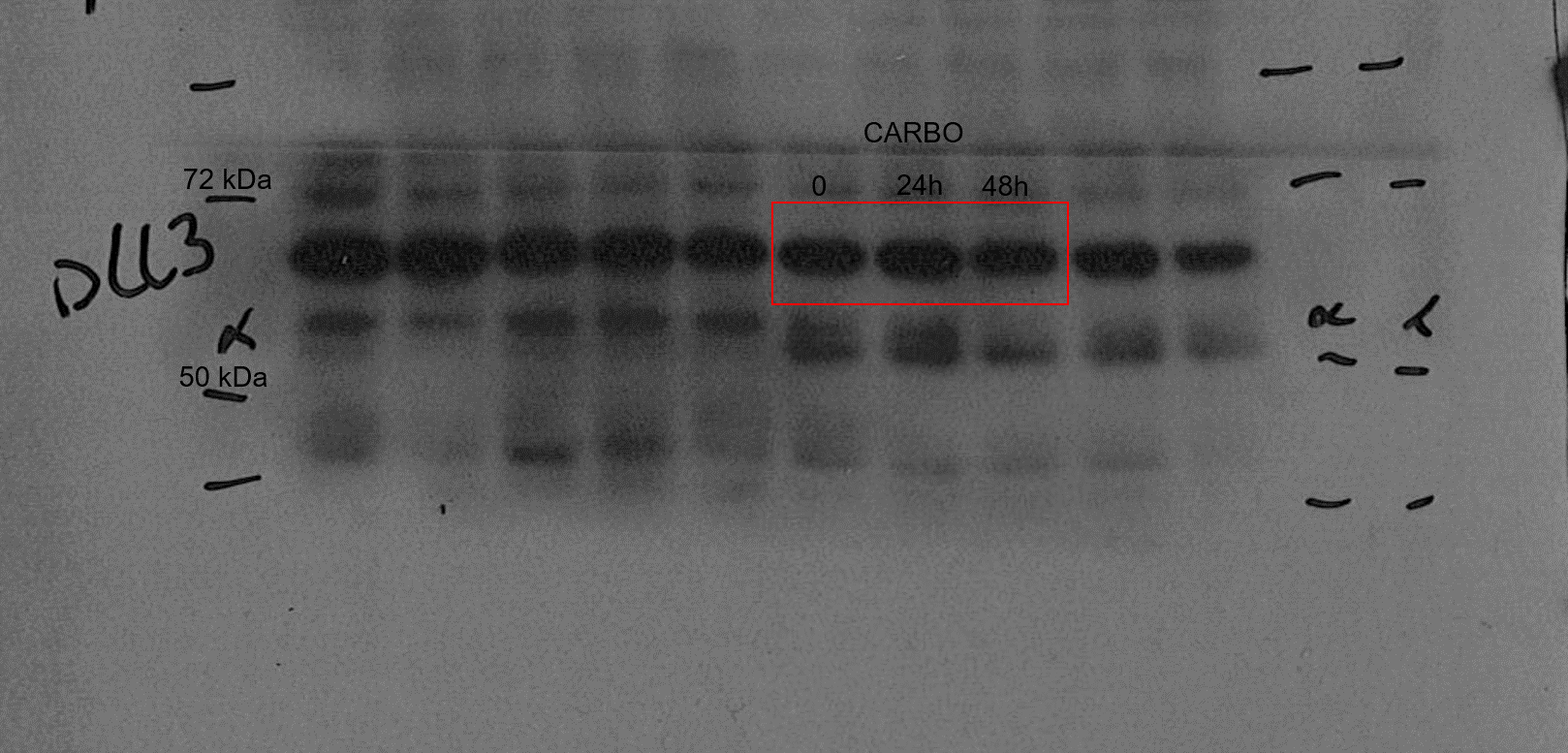

Supplement: Supplementary file 8 — Figure EV1 Source Data [file 44321_2025_354_MOESM8_ESM.zip › Fig EV1/Fig EV1/Fig EV1D/dll3, wb #3.png]

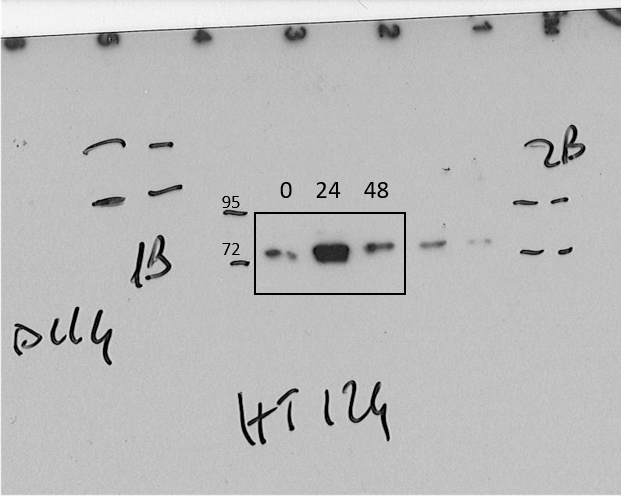

Supplement: Supplementary file 8 — Figure EV1 Source Data [file 44321_2025_354_MOESM8_ESM.zip › Fig EV1/Fig EV1/Fig EV1D/DLL4 WB#1.png]

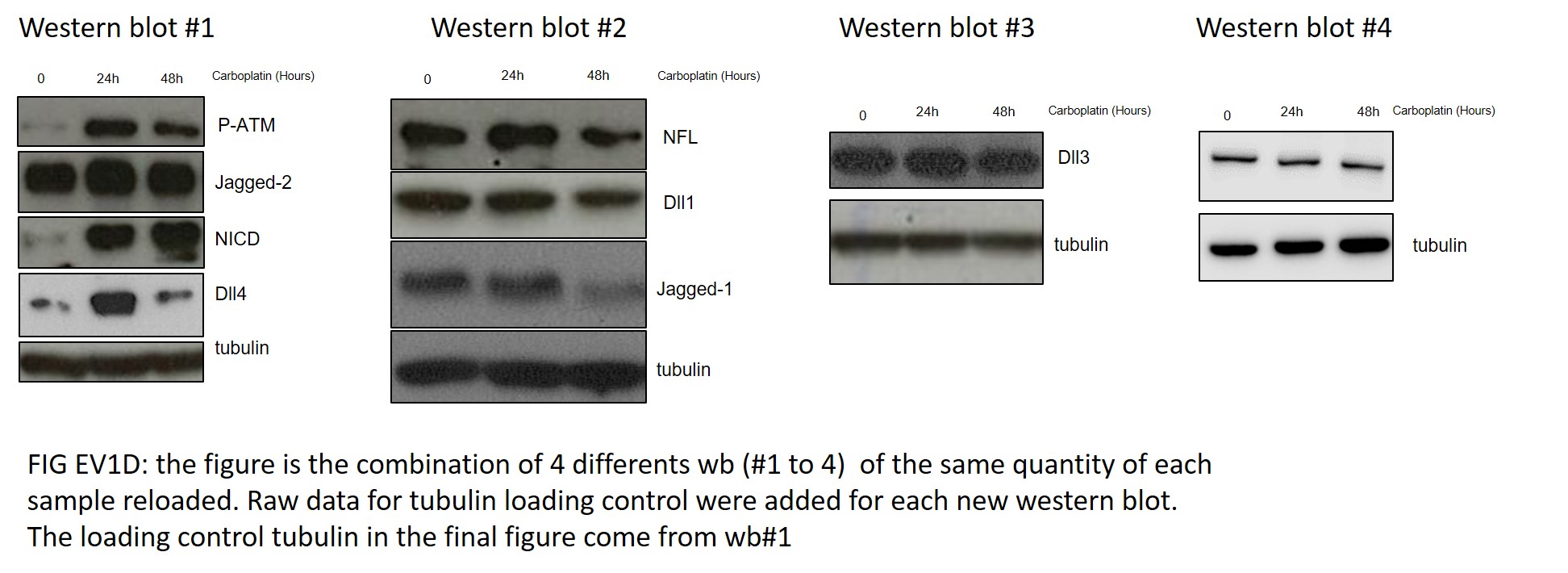

Supplement: Supplementary file 8 — Figure EV1 Source Data [file 44321_2025_354_MOESM8_ESM.zip › Fig EV1/Fig EV1/Fig EV1D/figEV1D.jpg]

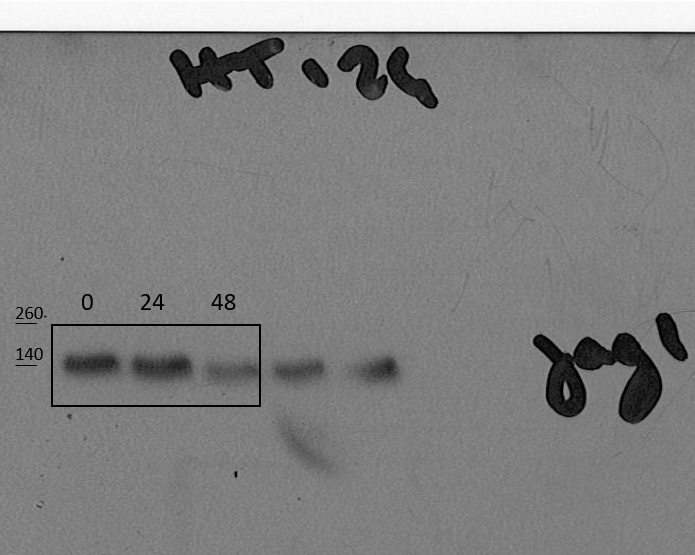

Supplement: Supplementary file 8 — Figure EV1 Source Data [file 44321_2025_354_MOESM8_ESM.zip › Fig EV1/Fig EV1/Fig EV1D/JAG1, wb#2.png]

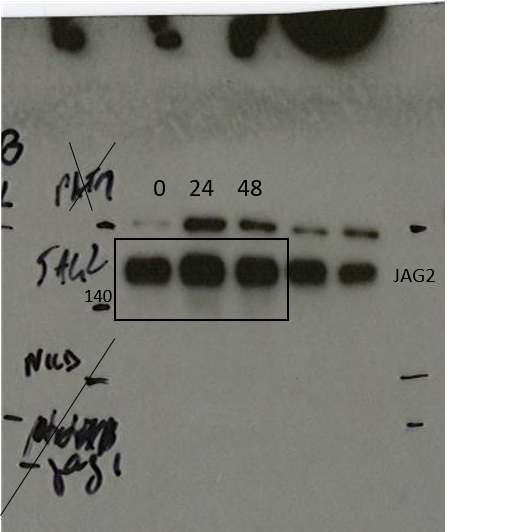

Supplement: Supplementary file 8 — Figure EV1 Source Data [file 44321_2025_354_MOESM8_ESM.zip › Fig EV1/Fig EV1/Fig EV1D/JAG2 #1.png]

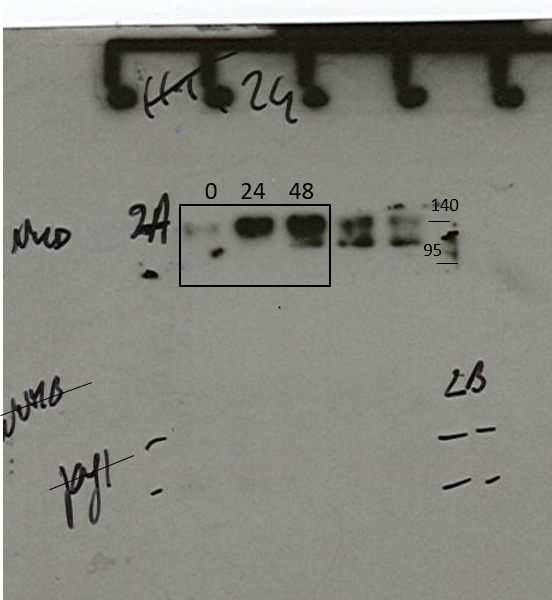

Supplement: Supplementary file 8 — Figure EV1 Source Data [file 44321_2025_354_MOESM8_ESM.zip › Fig EV1/Fig EV1/Fig EV1D/NICD#1.png]

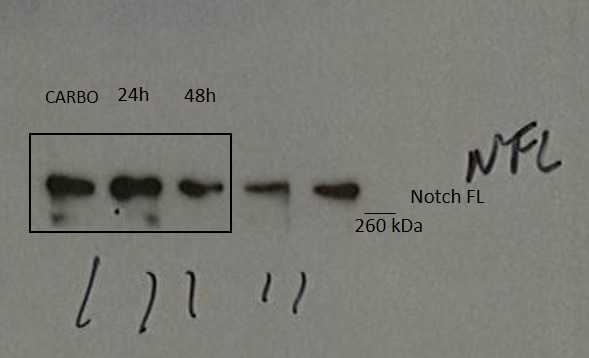

Supplement: Supplementary file 8 — Figure EV1 Source Data [file 44321_2025_354_MOESM8_ESM.zip › Fig EV1/Fig EV1/Fig EV1D/Notch FL wb#2.jpg]

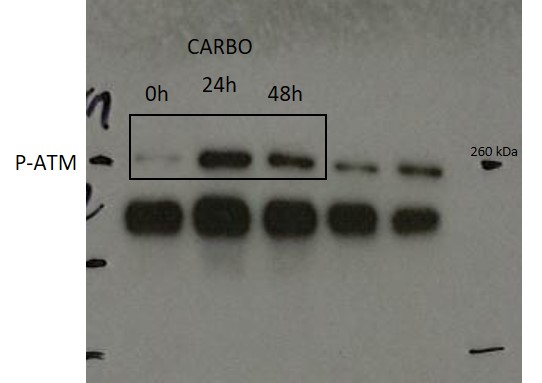

Supplement: Supplementary file 8 — Figure EV1 Source Data [file 44321_2025_354_MOESM8_ESM.zip › Fig EV1/Fig EV1/Fig EV1D/P-ATM, wb#1.png.jpg]

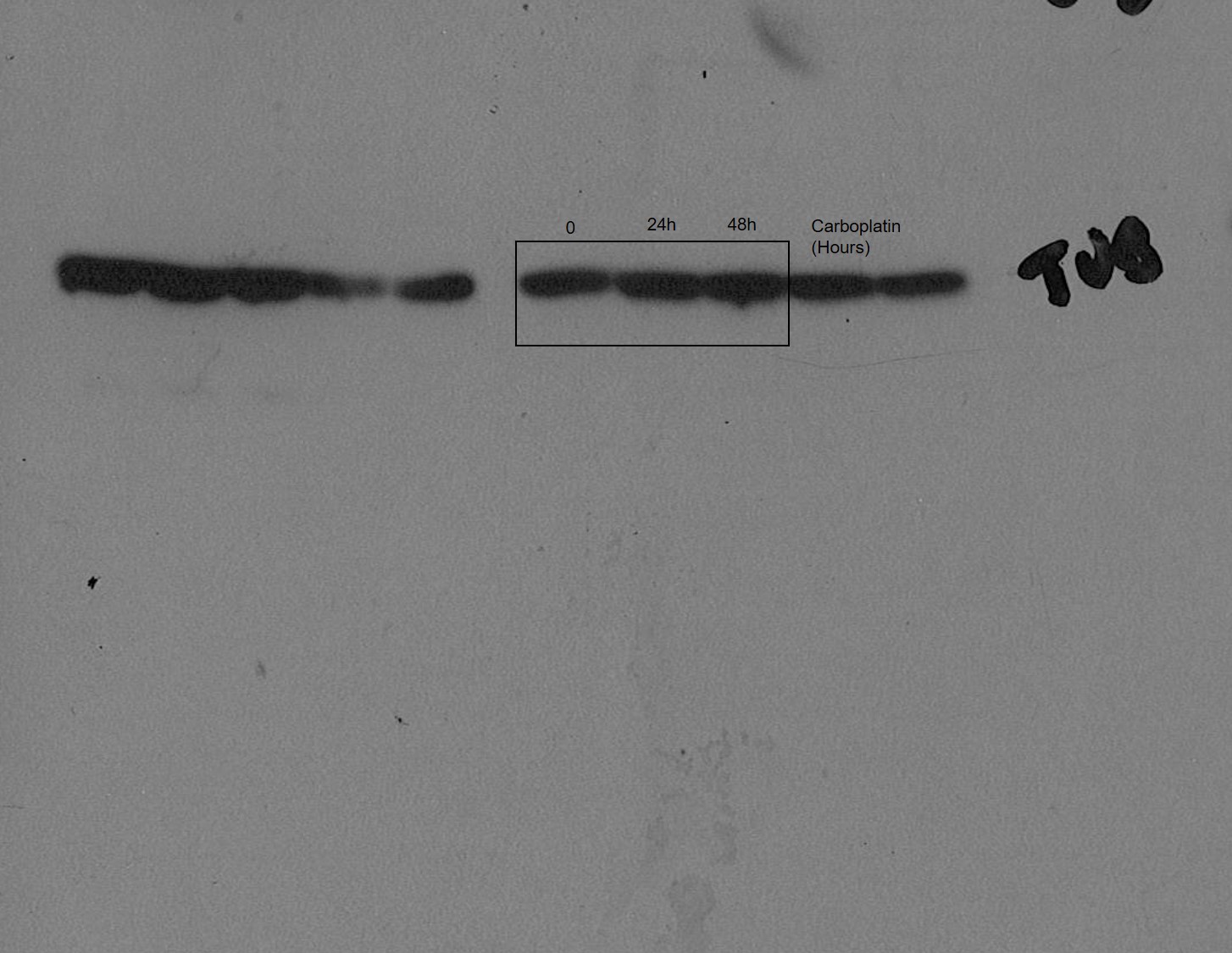

Supplement: Supplementary file 8 — Figure EV1 Source Data [file 44321_2025_354_MOESM8_ESM.zip › Fig EV1/Fig EV1/Fig EV1D/tubulin wb #2.jpg]

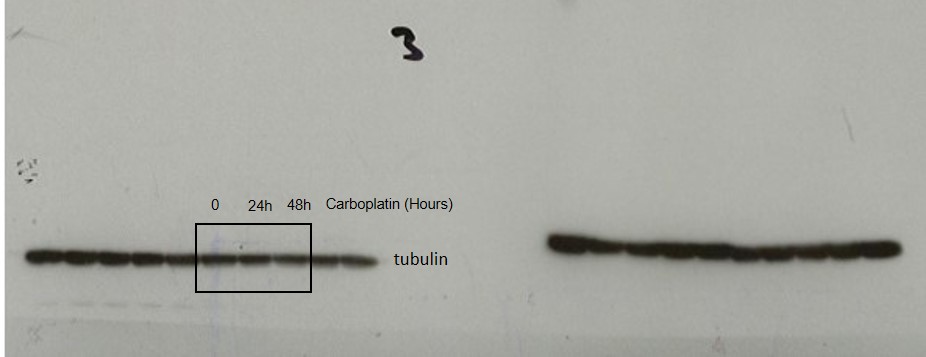

Supplement: Supplementary file 8 — Figure EV1 Source Data [file 44321_2025_354_MOESM8_ESM.zip › Fig EV1/Fig EV1/Fig EV1D/tubulin wb #3.jpg]

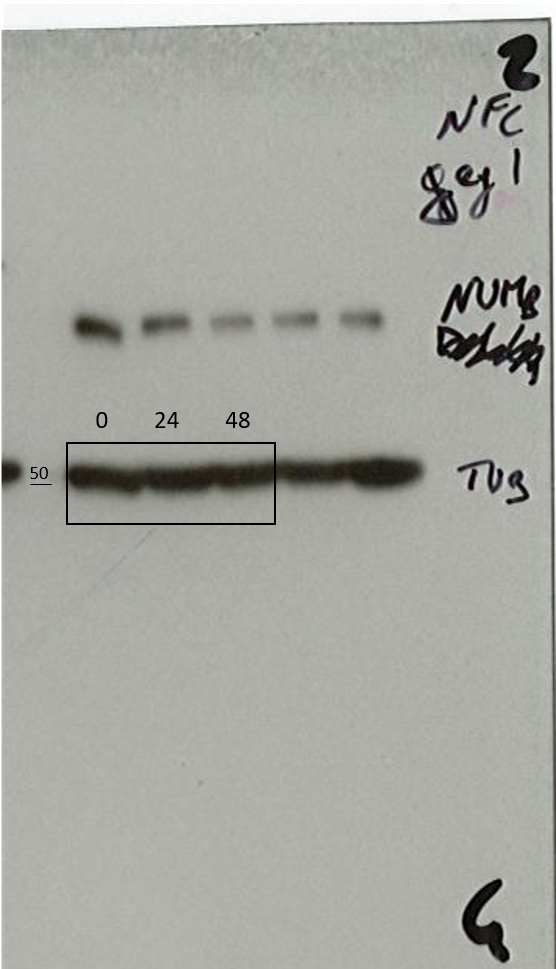

Supplement: Supplementary file 8 — Figure EV1 Source Data [file 44321_2025_354_MOESM8_ESM.zip › Fig EV1/Fig EV1/Fig EV1D/tubulin wb#1.png]

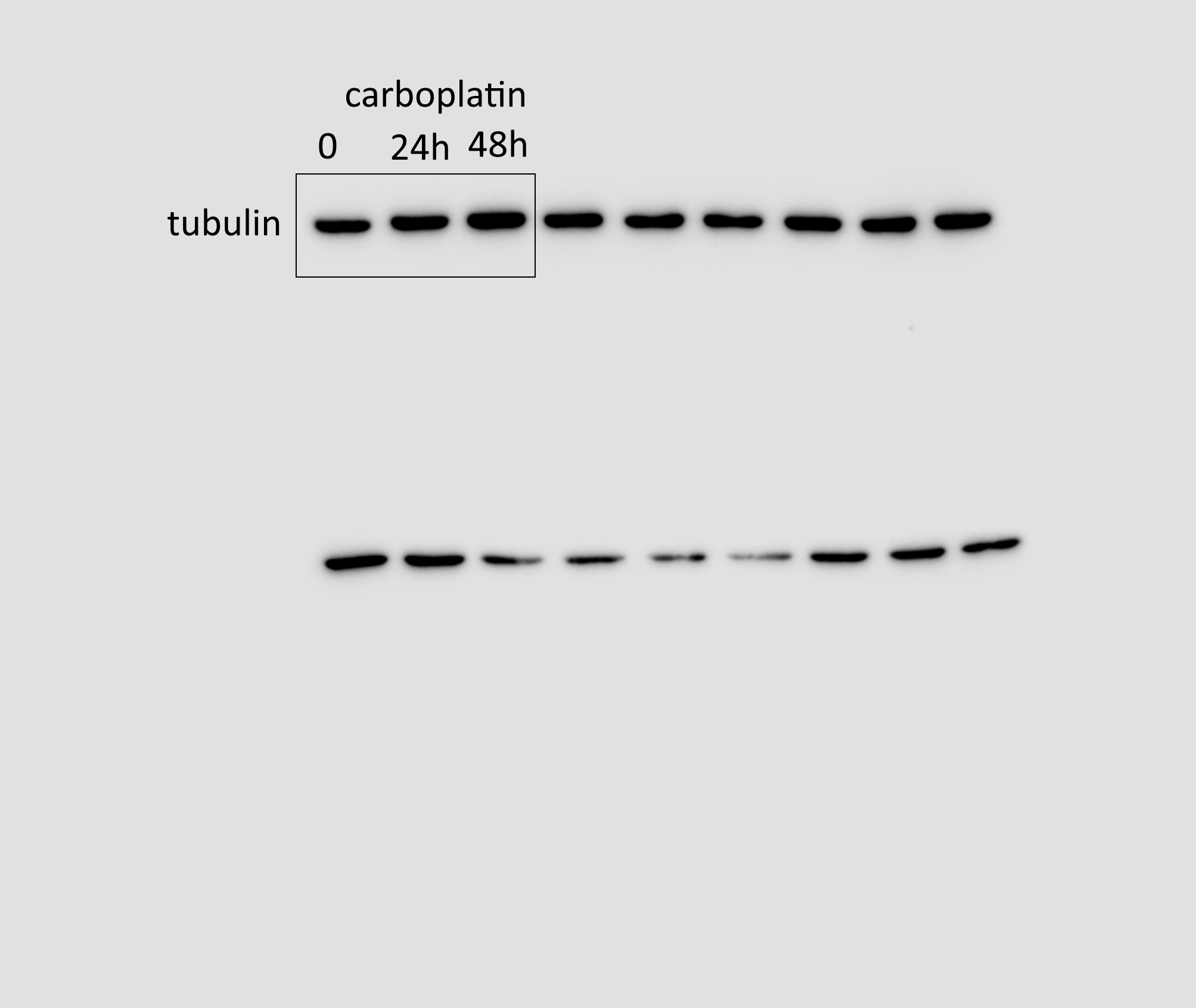

Supplement: Supplementary file 8 — Figure EV1 Source Data [file 44321_2025_354_MOESM8_ESM.zip › Fig EV1/Fig EV1/Fig EV1D/tubulin, wb#4.jpg]

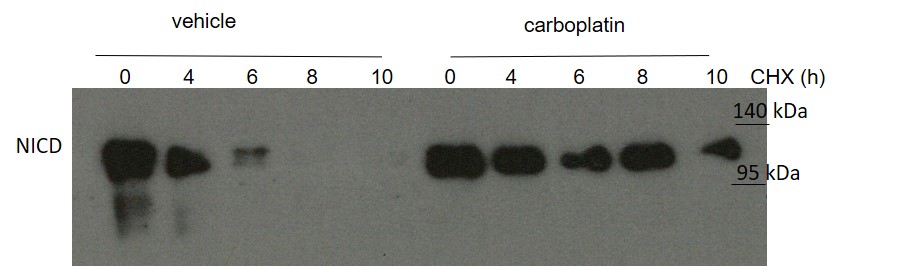

Supplement: Supplementary file 8 — Figure EV1 Source Data [file 44321_2025_354_MOESM8_ESM.zip › Fig EV1/Fig EV1/Fig EV1E/Fig EV1E replicat/western blot nicd N2.jpg]

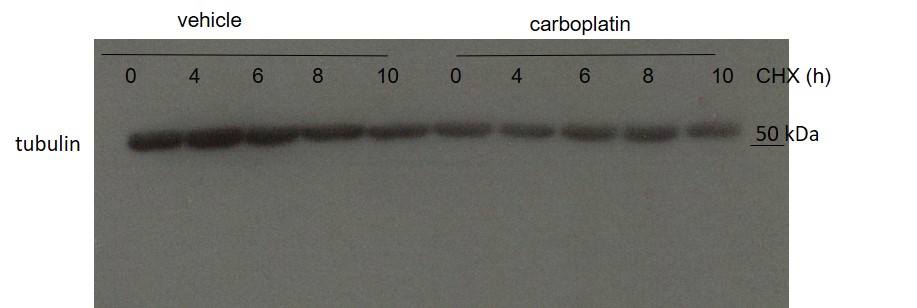

Supplement: Supplementary file 8 — Figure EV1 Source Data [file 44321_2025_354_MOESM8_ESM.zip › Fig EV1/Fig EV1/Fig EV1E/Fig EV1E replicat/western blot tubulin N2.jpg]

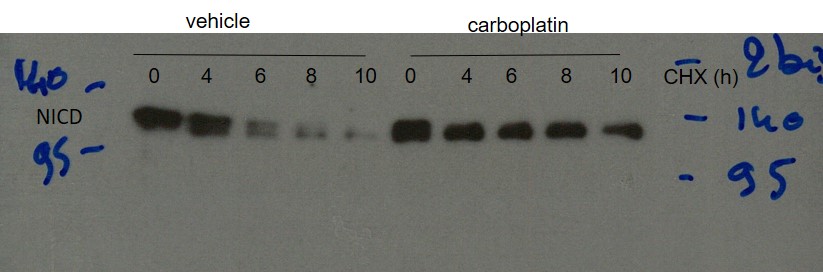

Supplement: Supplementary file 8 — Figure EV1 Source Data [file 44321_2025_354_MOESM8_ESM.zip › Fig EV1/Fig EV1/Fig EV1E/western blot nicd.jpg]

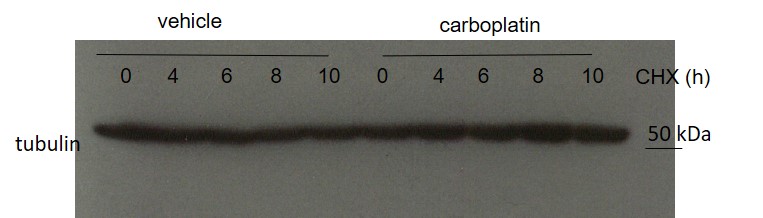

Supplement: Supplementary file 8 — Figure EV1 Source Data [file 44321_2025_354_MOESM8_ESM.zip › Fig EV1/Fig EV1/Fig EV1E/western blot tubulin.jpg]

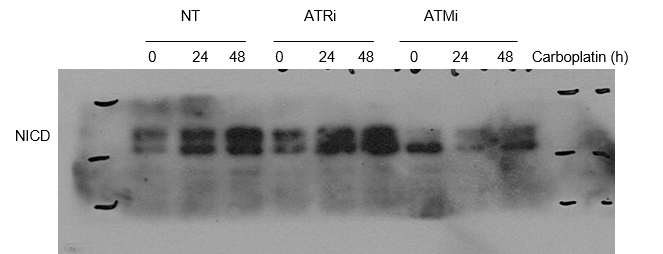

Supplement: Supplementary file 9 — Figure EV2 Source Data [file 44321_2025_354_MOESM9_ESM.zip › Fig EV2/Fig EV2A/Fig EV2A replicat/Western blot NICD N2.png]

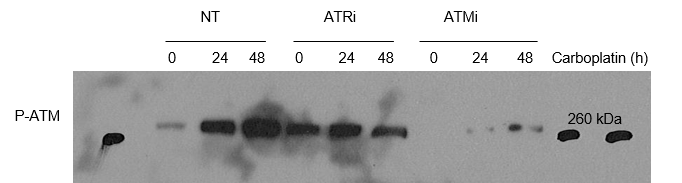

Supplement: Supplementary file 9 — Figure EV2 Source Data [file 44321_2025_354_MOESM9_ESM.zip › Fig EV2/Fig EV2A/Fig EV2A replicat/Western blot P-ATM N2.png]

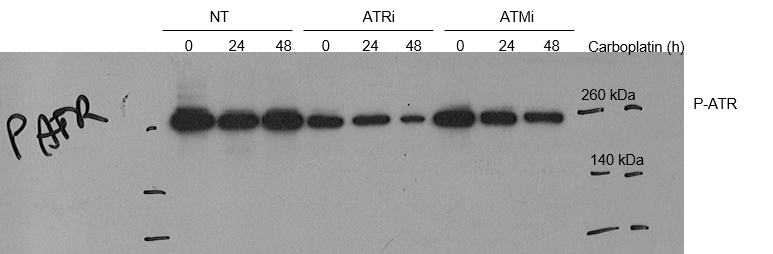

Supplement: Supplementary file 9 — Figure EV2 Source Data [file 44321_2025_354_MOESM9_ESM.zip › Fig EV2/Fig EV2A/Fig EV2A replicat/Western blot P-ATR N2.png]

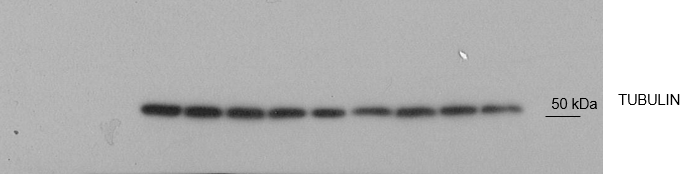

Supplement: Supplementary file 9 — Figure EV2 Source Data [file 44321_2025_354_MOESM9_ESM.zip › Fig EV2/Fig EV2A/Fig EV2A replicat/Western blot tubulin N2.png]

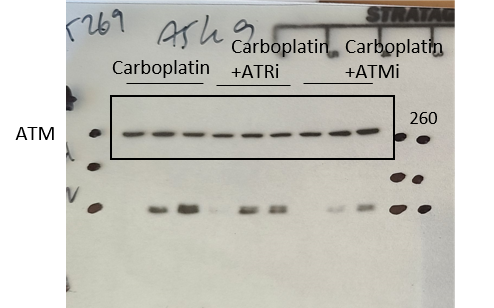

Supplement: Supplementary file 9 — Figure EV2 Source Data [file 44321_2025_354_MOESM9_ESM.zip › Fig EV2/Fig EV2A/Western blot-ATM.png]

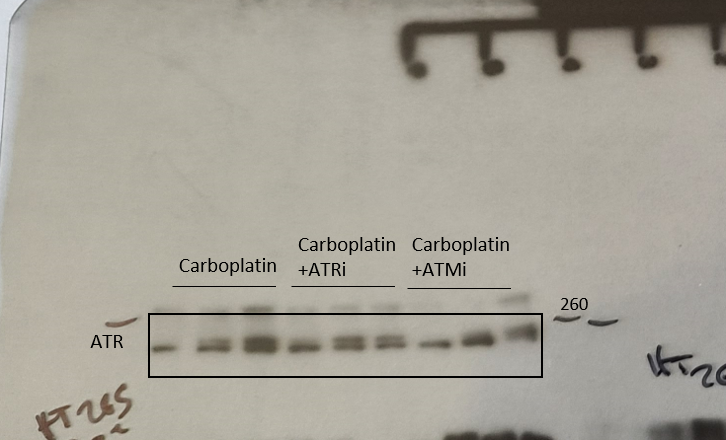

Supplement: Supplementary file 9 — Figure EV2 Source Data [file 44321_2025_354_MOESM9_ESM.zip › Fig EV2/Fig EV2A/Western blot-ATR.png]

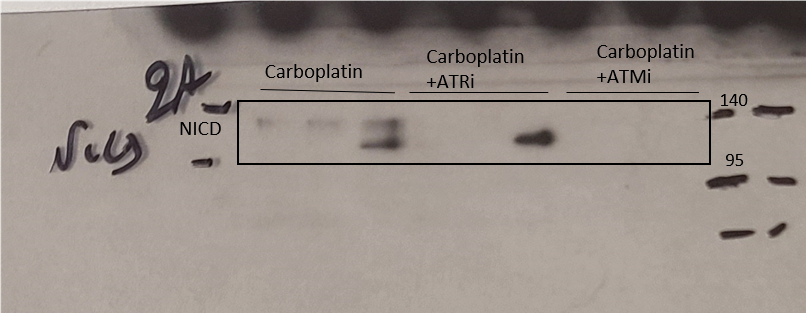

Supplement: Supplementary file 9 — Figure EV2 Source Data [file 44321_2025_354_MOESM9_ESM.zip › Fig EV2/Fig EV2A/Western blot-NICD.png]

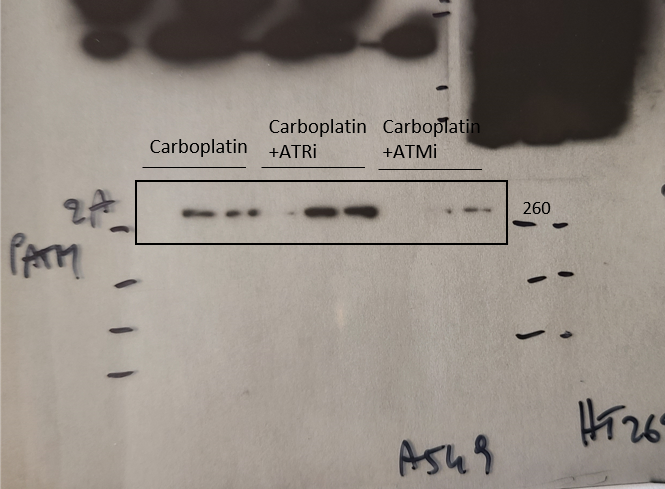

Supplement: Supplementary file 9 — Figure EV2 Source Data [file 44321_2025_354_MOESM9_ESM.zip › Fig EV2/Fig EV2A/Western blot-P-ATM.png]

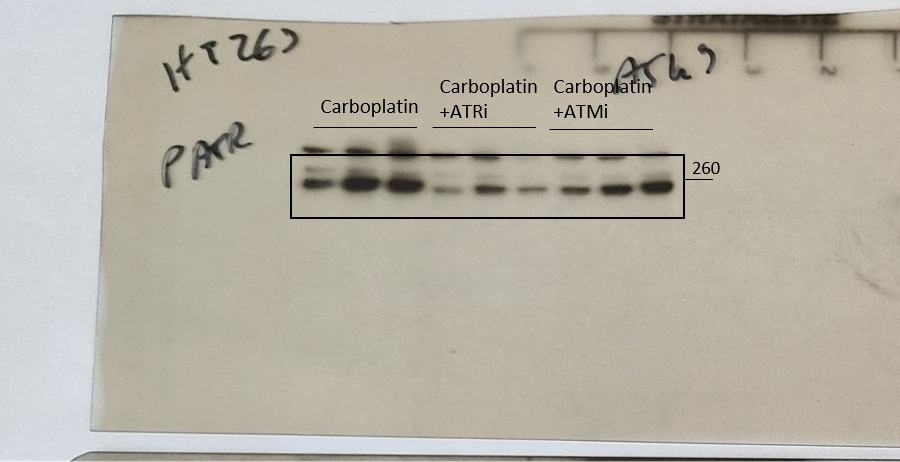

Supplement: Supplementary file 9 — Figure EV2 Source Data [file 44321_2025_354_MOESM9_ESM.zip › Fig EV2/Fig EV2A/Western blot-P-ATR.png]

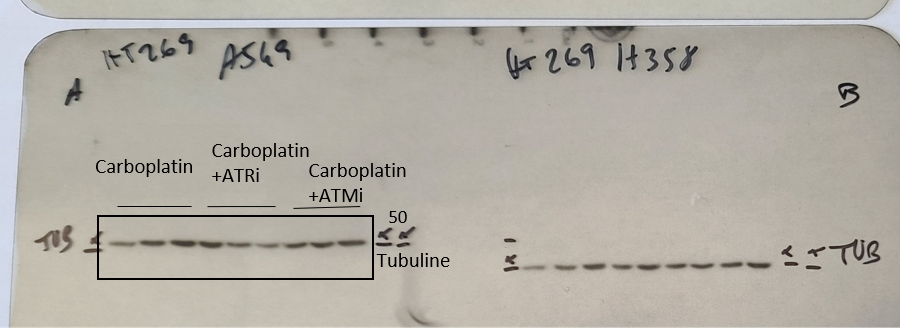

Supplement: Supplementary file 9 — Figure EV2 Source Data [file 44321_2025_354_MOESM9_ESM.zip › Fig EV2/Fig EV2A/Western blot-tubulin.png]

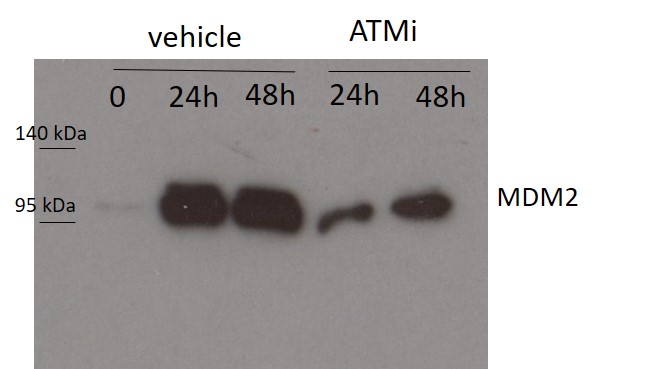

Supplement: Supplementary file 9 — Figure EV2 Source Data [file 44321_2025_354_MOESM9_ESM.zip › Fig EV2/Fig EV2B/FigEV2B replicat/western blot MDM2 N2.jpg]

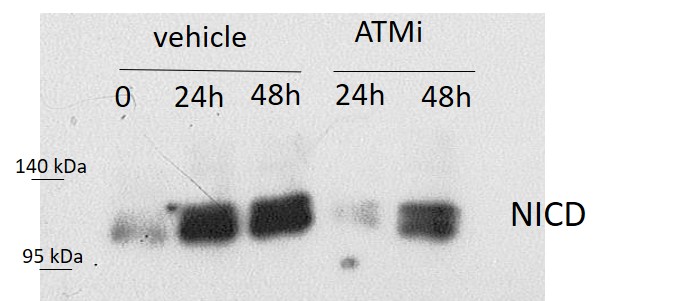

Supplement: Supplementary file 9 — Figure EV2 Source Data [file 44321_2025_354_MOESM9_ESM.zip › Fig EV2/Fig EV2B/FigEV2B replicat/western blot nicd N2.jpg]

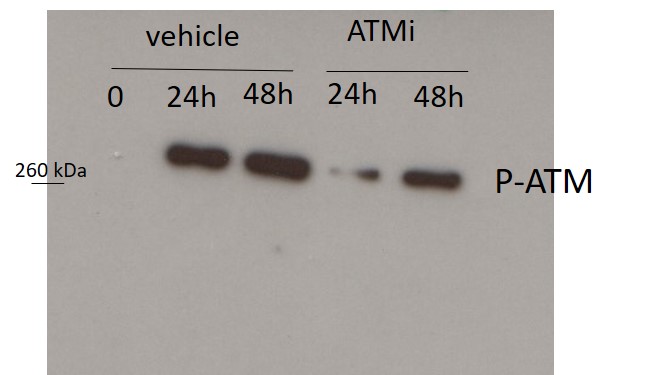

Supplement: Supplementary file 9 — Figure EV2 Source Data [file 44321_2025_354_MOESM9_ESM.zip › Fig EV2/Fig EV2B/FigEV2B replicat/western blot P-ATM N2.jpg]

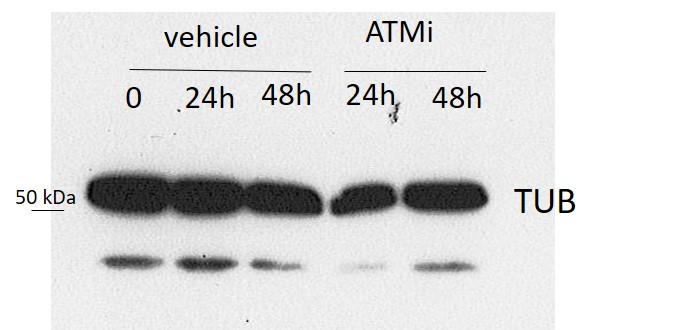

Supplement: Supplementary file 9 — Figure EV2 Source Data [file 44321_2025_354_MOESM9_ESM.zip › Fig EV2/Fig EV2B/FigEV2B replicat/western blot tubulin N2.jpg]

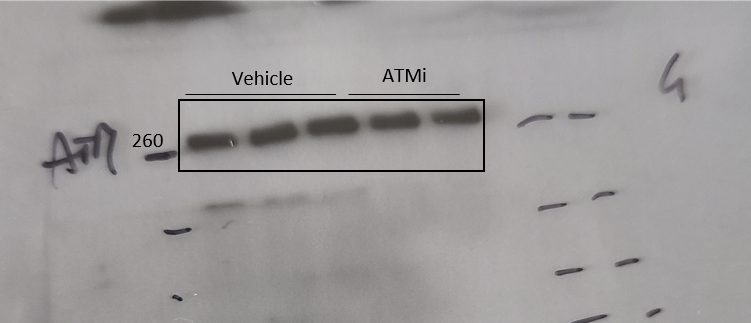

Supplement: Supplementary file 9 — Figure EV2 Source Data [file 44321_2025_354_MOESM9_ESM.zip › Fig EV2/Fig EV2B/western blot ATM.png]

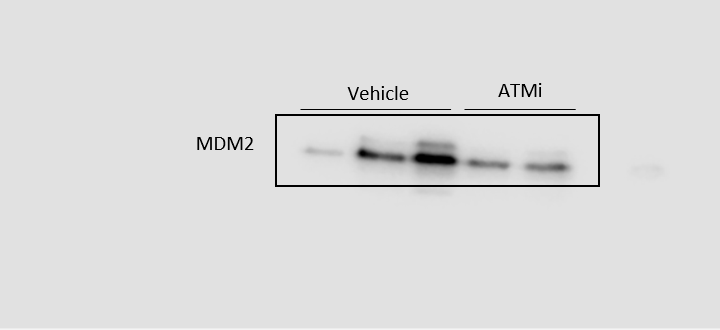

Supplement: Supplementary file 9 — Figure EV2 Source Data [file 44321_2025_354_MOESM9_ESM.zip › Fig EV2/Fig EV2B/western blot MDM2.png]

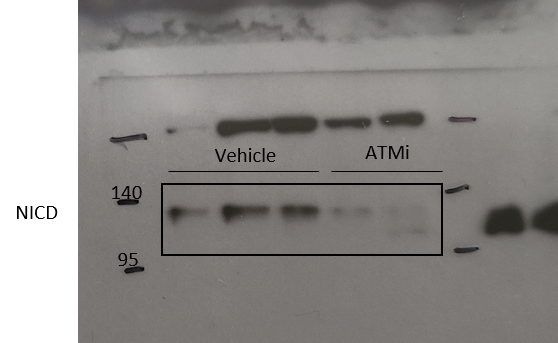

Supplement: Supplementary file 9 — Figure EV2 Source Data [file 44321_2025_354_MOESM9_ESM.zip › Fig EV2/Fig EV2B/western blot NICD.png]

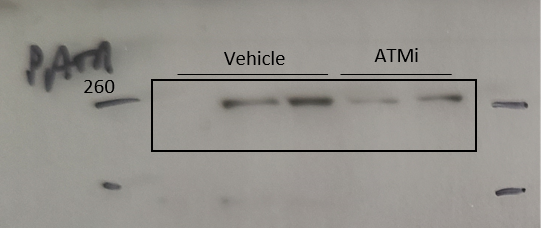

Supplement: Supplementary file 9 — Figure EV2 Source Data [file 44321_2025_354_MOESM9_ESM.zip › Fig EV2/Fig EV2B/western blot P-ATM.png]

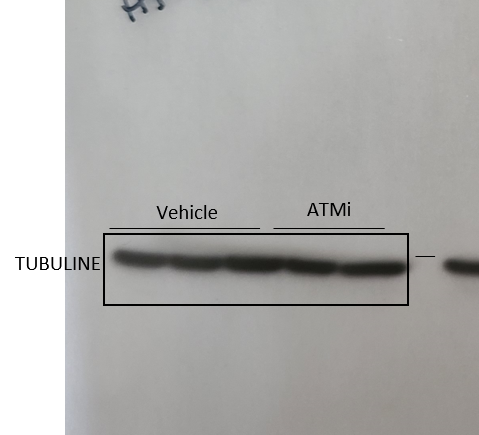

Supplement: Supplementary file 9 — Figure EV2 Source Data [file 44321_2025_354_MOESM9_ESM.zip › Fig EV2/Fig EV2B/western blot TUBULIN.png]

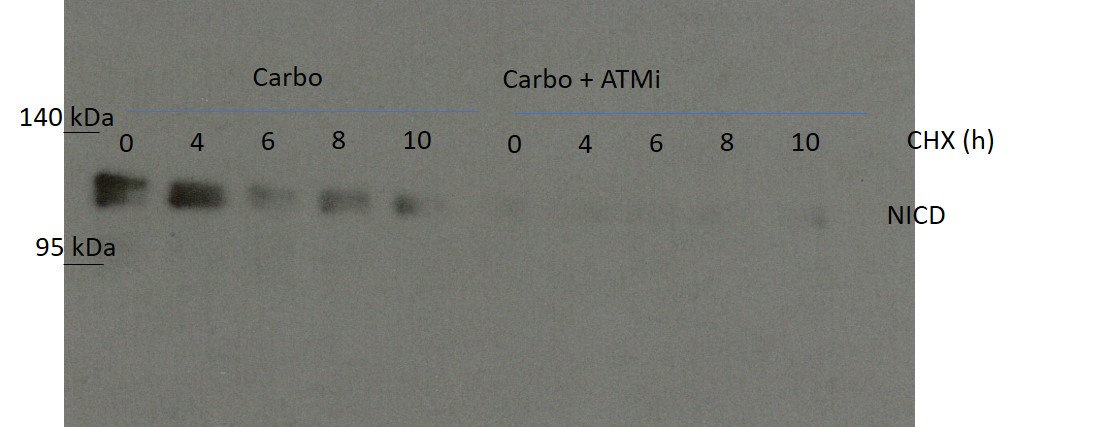

Supplement: Supplementary file 9 — Figure EV2 Source Data [file 44321_2025_354_MOESM9_ESM.zip › Fig EV2/Fig EV2C/Fig EV2C replicat/western blot nicd N2.jpg]

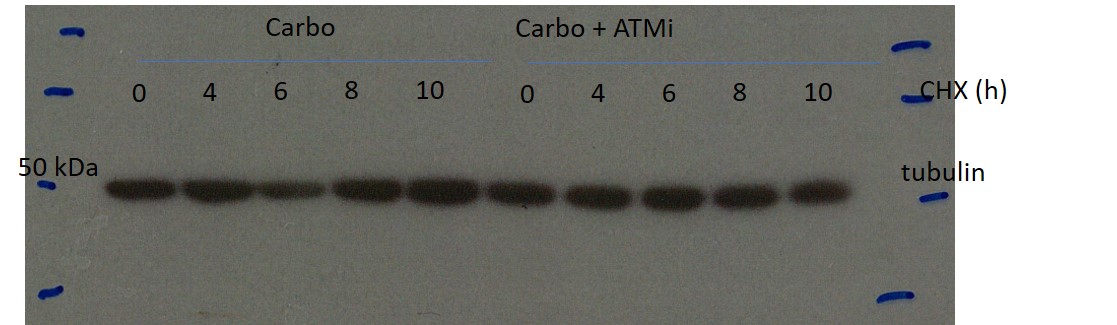

Supplement: Supplementary file 9 — Figure EV2 Source Data [file 44321_2025_354_MOESM9_ESM.zip › Fig EV2/Fig EV2C/Fig EV2C replicat/western blot TUBULIN N2.jpg]

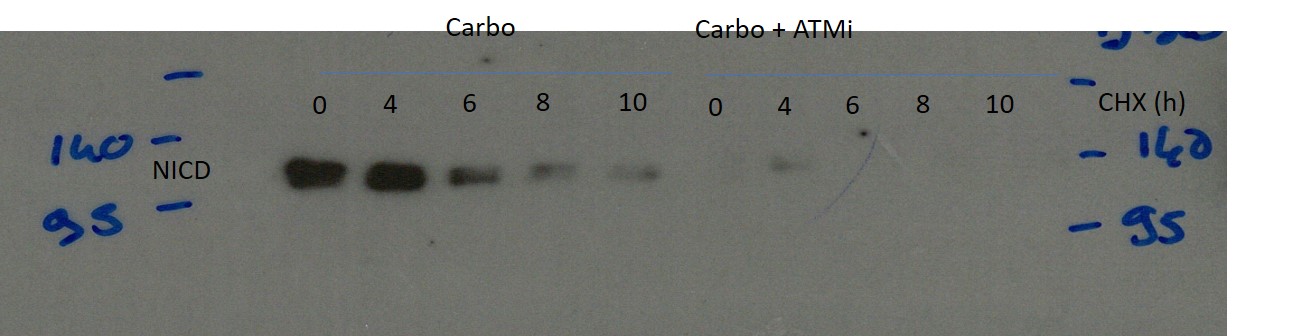

Supplement: Supplementary file 9 — Figure EV2 Source Data [file 44321_2025_354_MOESM9_ESM.zip › Fig EV2/Fig EV2C/western blot nicd.jpg]

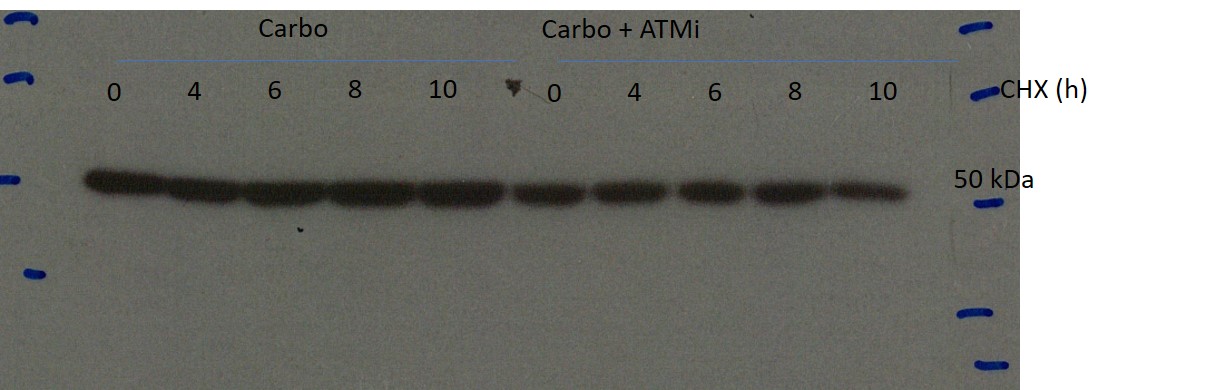

Supplement: Supplementary file 9 — Figure EV2 Source Data [file 44321_2025_354_MOESM9_ESM.zip › Fig EV2/Fig EV2C/western blot tubulin.jpg]

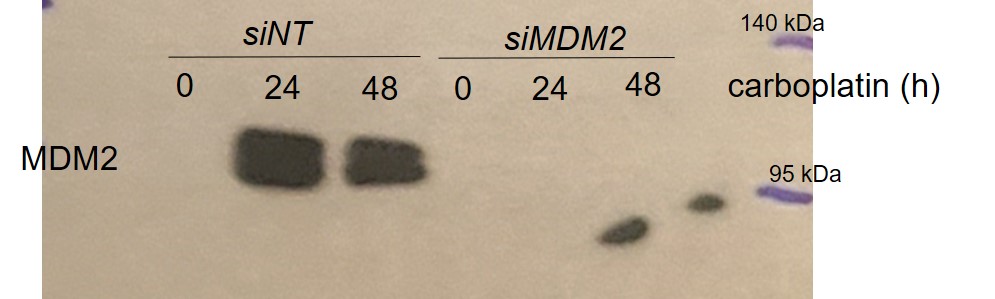

Supplement: Supplementary file 9 — Figure EV2 Source Data [file 44321_2025_354_MOESM9_ESM.zip › Fig EV2/Fig EV2D/Fig EV2D replicat/western blot MDM2 N2.jpg]

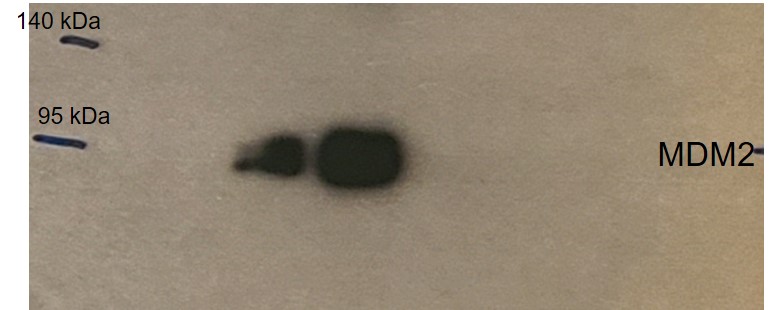

Supplement: Supplementary file 9 — Figure EV2 Source Data [file 44321_2025_354_MOESM9_ESM.zip › Fig EV2/Fig EV2D/Fig EV2D replicat/western blot MDM2 N3.jpg]

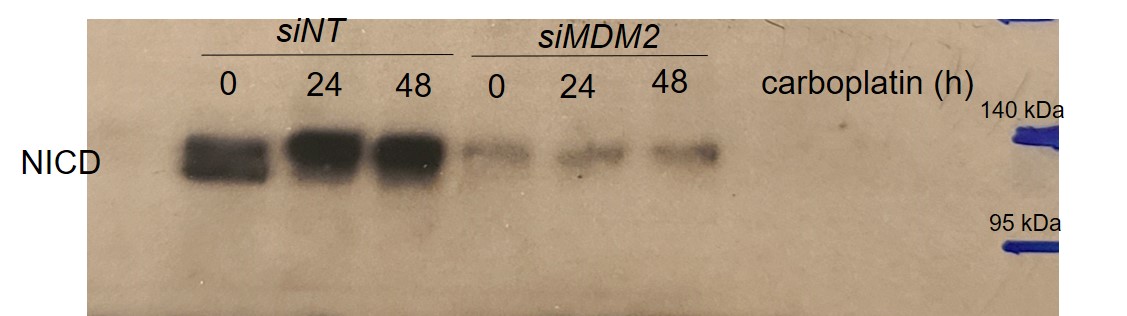

Supplement: Supplementary file 9 — Figure EV2 Source Data [file 44321_2025_354_MOESM9_ESM.zip › Fig EV2/Fig EV2D/Fig EV2D replicat/western blot NICD N2.jpg]

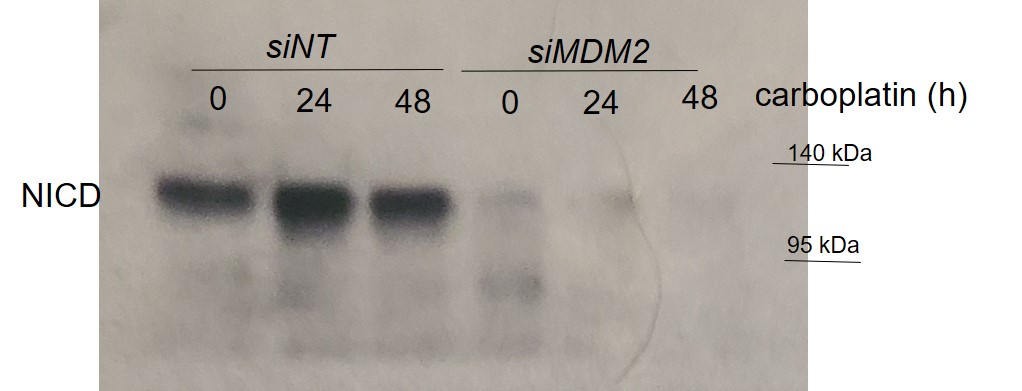

Supplement: Supplementary file 9 — Figure EV2 Source Data [file 44321_2025_354_MOESM9_ESM.zip › Fig EV2/Fig EV2D/Fig EV2D replicat/western blot NICD N3.jpg]

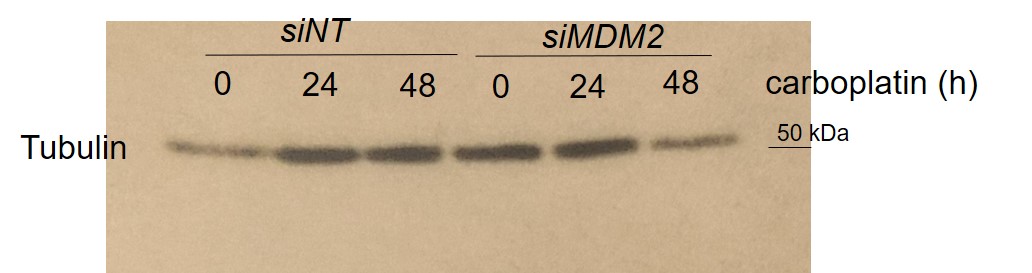

Supplement: Supplementary file 9 — Figure EV2 Source Data [file 44321_2025_354_MOESM9_ESM.zip › Fig EV2/Fig EV2D/Fig EV2D replicat/western blot tubulin N2.jpg]

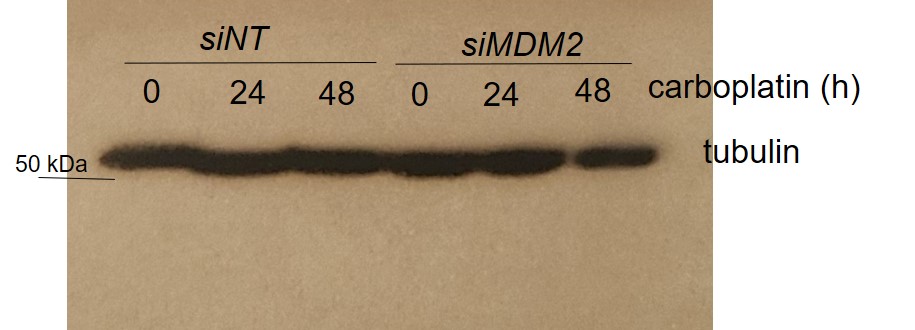

Supplement: Supplementary file 9 — Figure EV2 Source Data [file 44321_2025_354_MOESM9_ESM.zip › Fig EV2/Fig EV2D/Fig EV2D replicat/western blot tubulin N3.jpg]

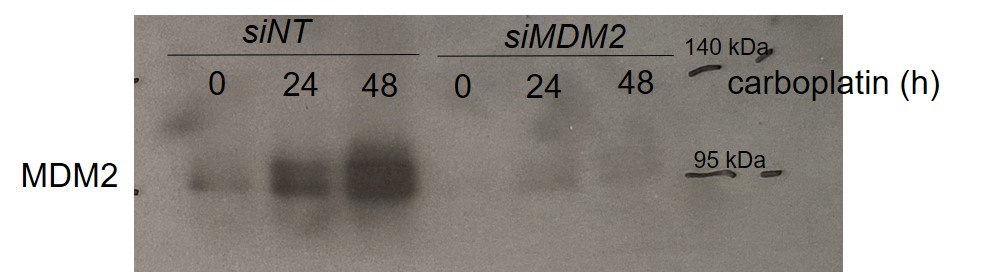

Supplement: Supplementary file 9 — Figure EV2 Source Data [file 44321_2025_354_MOESM9_ESM.zip › Fig EV2/Fig EV2D/WESTERN BLOT mdm2.jpg]

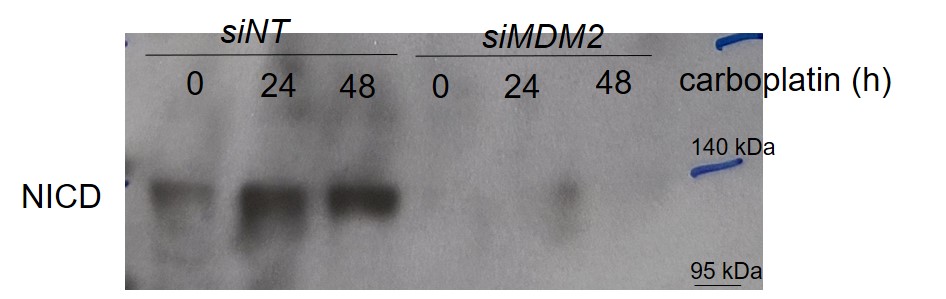

Supplement: Supplementary file 9 — Figure EV2 Source Data [file 44321_2025_354_MOESM9_ESM.zip › Fig EV2/Fig EV2D/WESTERN BLOT NICD.jpg]

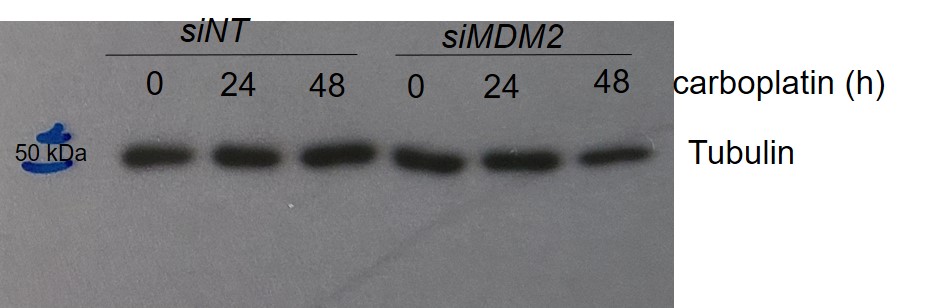

Supplement: Supplementary file 9 — Figure EV2 Source Data [file 44321_2025_354_MOESM9_ESM.zip › Fig EV2/Fig EV2D/WESTERN BLOT TUBULIN.jpg]
