# Supplementary figures and images for: NOTCH1 intracellular domain stabilization by MDM2 plays a major role in NSCLC response to platinum (part 3 of 3)
Source: EMBO Mol Med. 2026 Jan 16;18(2):514–41. doi: 10.1038/s44321-025-00354-9 (PMC12905330; doi:10.1038/s44321-025-00354-9)

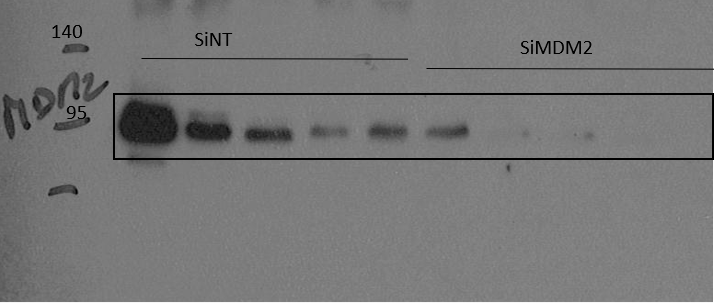

Supplement: Supplementary file 9 — Figure EV2 Source Data [file 44321_2025_354_MOESM9_ESM.zip › Fig EV2/Fig EV2E/Fig EV2E replicat/western blot MDM2 N2.png]

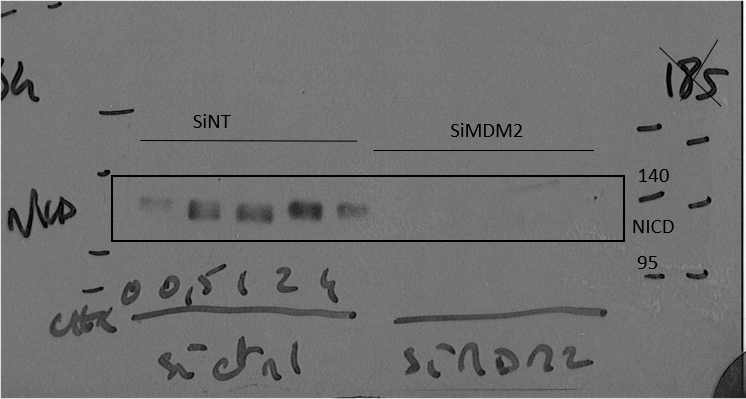

Supplement: Supplementary file 9 — Figure EV2 Source Data [file 44321_2025_354_MOESM9_ESM.zip › Fig EV2/Fig EV2E/Fig EV2E replicat/western blot NICD N2.png]

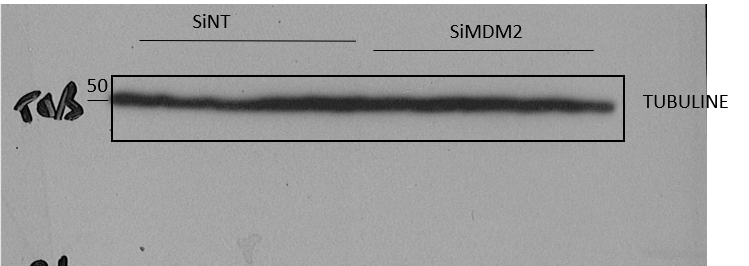

Supplement: Supplementary file 9 — Figure EV2 Source Data [file 44321_2025_354_MOESM9_ESM.zip › Fig EV2/Fig EV2E/Fig EV2E replicat/western blot TUBULIN N2.png]

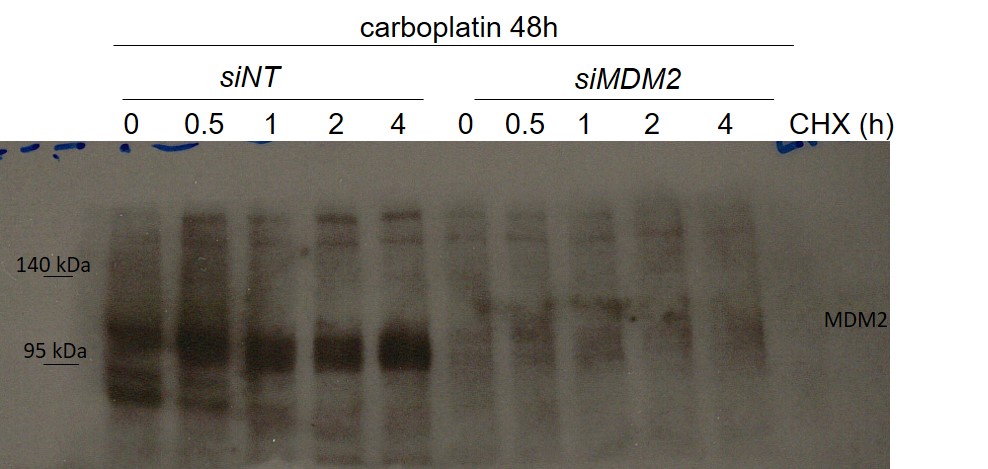

Supplement: Supplementary file 9 — Figure EV2 Source Data [file 44321_2025_354_MOESM9_ESM.zip › Fig EV2/Fig EV2E/western blot MDM2.jpg]

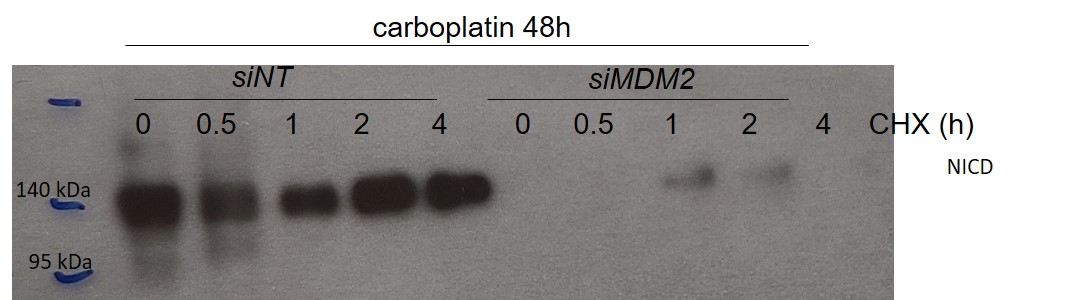

Supplement: Supplementary file 9 — Figure EV2 Source Data [file 44321_2025_354_MOESM9_ESM.zip › Fig EV2/Fig EV2E/western blot NICD.jpg]

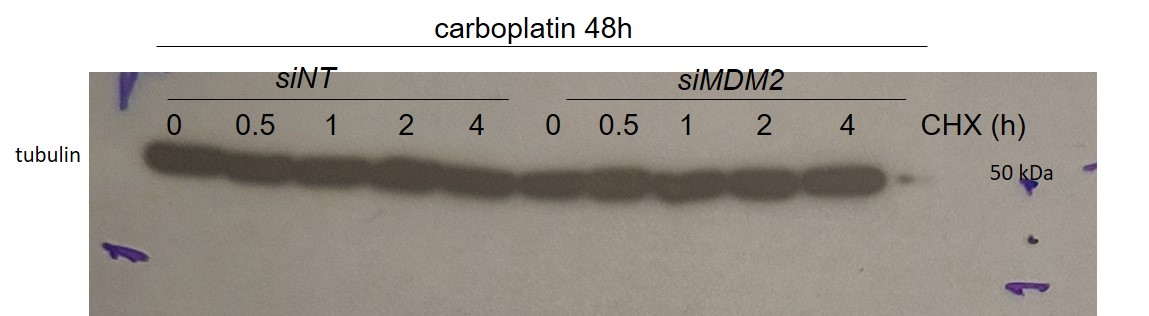

Supplement: Supplementary file 9 — Figure EV2 Source Data [file 44321_2025_354_MOESM9_ESM.zip › Fig EV2/Fig EV2E/western blot tubulin.jpg]

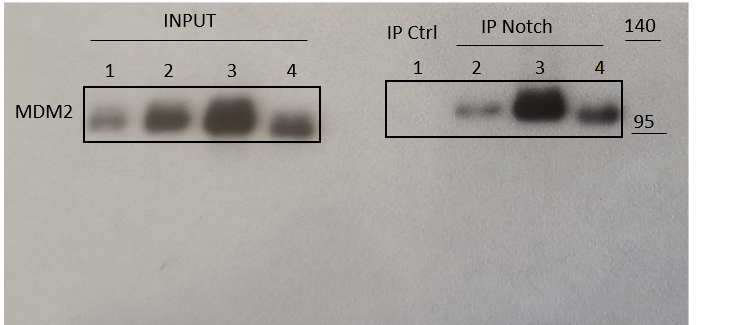

Supplement: Supplementary file 10 — Figure EV3 Source Data [file 44321_2025_354_MOESM10_ESM.zip › Fig EV3/Western blot MDM2 INPUT-IP.png]

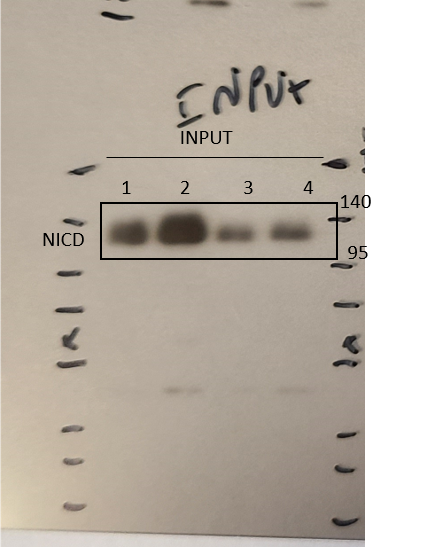

Supplement: Supplementary file 10 — Figure EV3 Source Data [file 44321_2025_354_MOESM10_ESM.zip › Fig EV3/Western blot NICD INPUT.png]

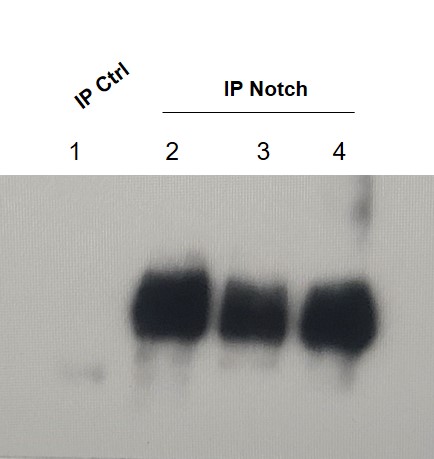

Supplement: Supplementary file 10 — Figure EV3 Source Data [file 44321_2025_354_MOESM10_ESM.zip › Fig EV3/western blot nicd- IP.jpg]

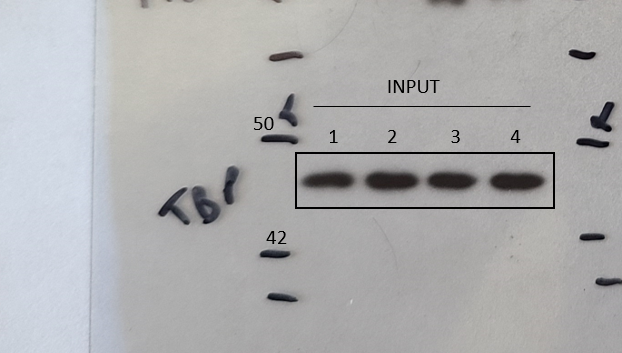

Supplement: Supplementary file 10 — Figure EV3 Source Data [file 44321_2025_354_MOESM10_ESM.zip › Fig EV3/Western blot TBP INPUT.png]
