# Supplementary material for: NOTCH1 intracellular domain stabilization by MDM2 plays a major role in NSCLC response to platinum
Source: EMBO Mol Med. 2026 Jan 16;18(2):514–41. doi: 10.1038/s44321-025-00354-9 (PMC12905330; doi:10.1038/s44321-025-00354-9)
Supplement: Supplementary file 13 — Expanded View Figures [file 44321_2025_354_MOESM13_ESM.pdf]

Expanded View Figures

Figure EV1. DNA damage induces NICD stabilization.

(**A**) Western blotting of the indicated proteins in A549 cells exposed to 100 µM Carboplatin ($n = 2$), 25 µM irinotecan ($n = 2$), or γ-irradiation (4 Gy) ($n = 1$) for the indicated time. P-ATM, phosphorylated (activated) ATM. (**B**) Immunofluorescence analysis of γH2AX (in red) and 53BP1 (in green) in A549 cells after 24 or 48 h of incubation with 100 µM Carboplatin ($n = 1$). Nuclei were stained with DAPI (in blue). Scale bar: 10 µm. (**C**) Western blotting of the indicated proteins after cellular fractionation of A549 cells incubated (+) or not (−) with 100 µM Carboplatin for 48 h ($n = 2$). S1, cytoplasmic fraction; S2, nucleoplasmic fraction; P2, chromatin fraction; TUBULIN, loading control for S1; TBP, loading control for P2. (**D**) Western blotting of the indicated proteins in A549 cells incubated with 100 µM Carboplatin for the indicated time ($n = 1$). (**E**) Western blotting of the indicated proteins in A549 cells incubated or not with 100 µM Carboplatin for 48 h and with 50 µg/µl Cycloheximide (CHX) for the indicated time before the end of Carboplatin treatment ($n = 2$). Upper panel: Schematic representation of the experimental design. In the legend, *n* indicates the number of biological replicates. Source data are available online for this figure.

 © The Author(s)

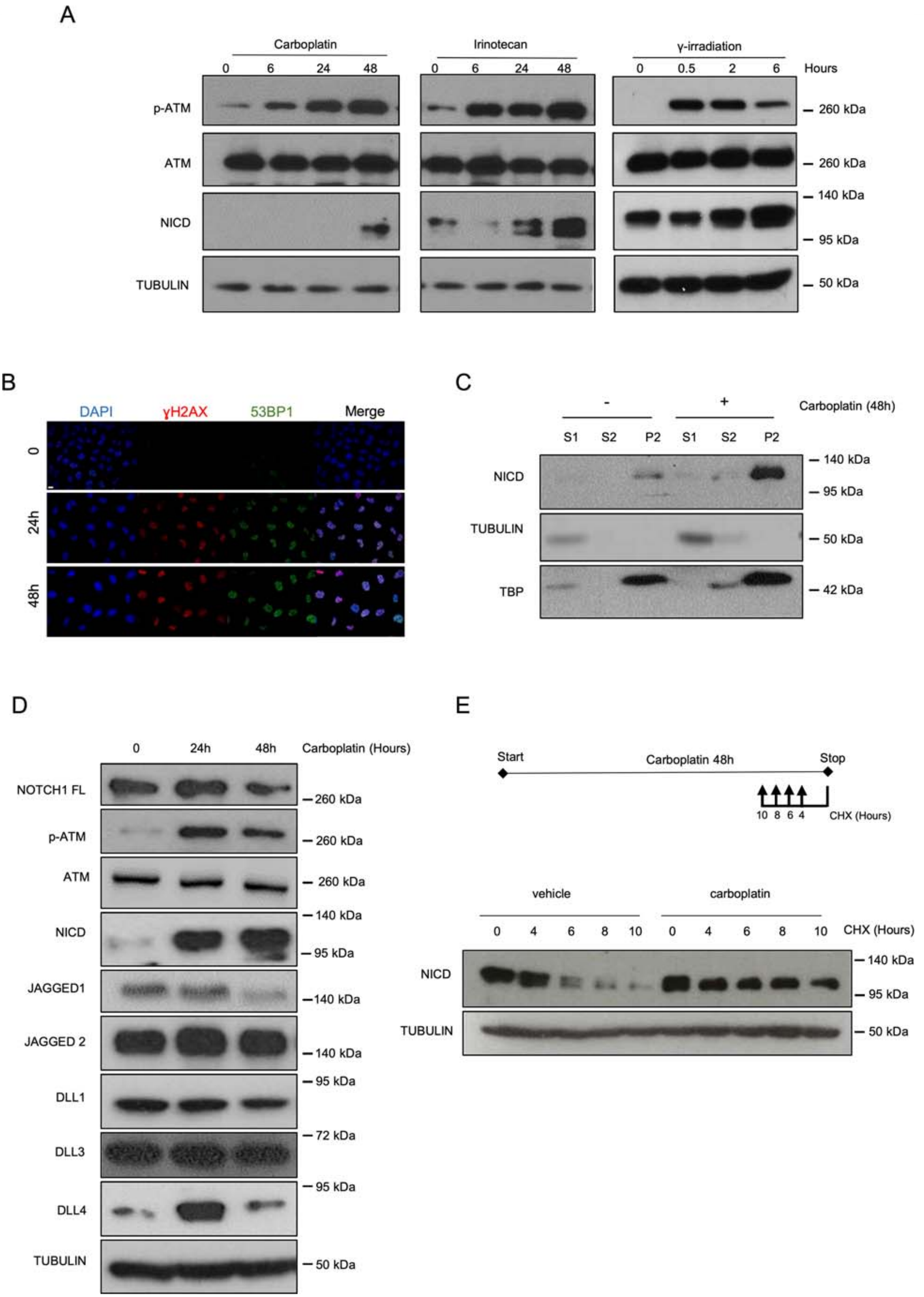

A
Carboplatin
Irinotecan
γ-irradiation
0 6 24 48
0 6 24 48
0 0.5 2 6 Hours
p-ATM
ATM
NICD
TUBULIN
260 kDa
260 kDa
140 kDa
95 kDa
50 kDa
B
DAPI
γH2AX
53BP1
Merge
0
24h
48h
C
-
+
Carboplatin (48h)
S1 S2 P2 S1 S2 P2
NICD
TUBULIN
TBP
140 kDa
95 kDa
50 kDa
42 kDa
D
0 24h 48h Carboplatin (Hours)
NOTCH1 FL
p-ATM
ATM
NICD
JAGGED1
JAGGED 2
DLL1
DLL3
DLL4
TUBULIN
260 kDa
260 kDa
260 kDa
140 kDa
95 kDa
140 kDa
140 kDa
95 kDa
72 kDa
95 kDa
50 kDa
E
Start
Carboplatin 48h
Stop
10 8 6 4
CHX (Hours)
vehicle
carboplatin
0 4 6 8 10 0 4 6 8 10 CHX (Hours)
NICD
TUBULIN
140 kDa
95 kDa
50 kDa

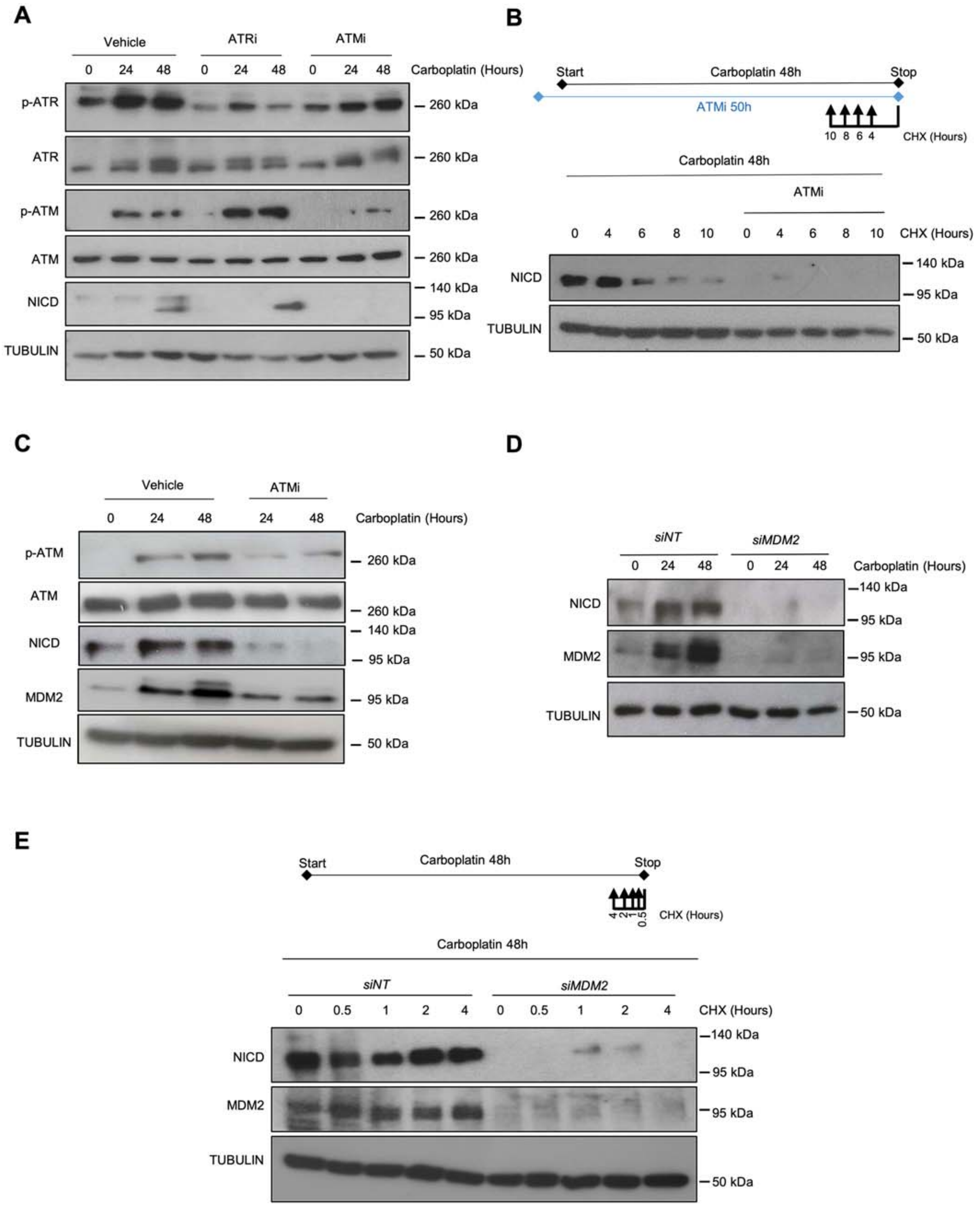
A
Vehicle
ATRi
ATMi
0 24 48 0 24 48 0 24 48 Carboplatin (Hours)
p-ATR
ATR
p-ATM
ATM
NICD
TUBULIN
260 kDa
260 kDa
260 kDa
260 kDa
140 kDa
95 kDa
50 kDa
B
Start
Carboplatin 48h
Stop
ATMi 50h
10 8 6 4
CHX (Hours)
Carboplatin 48h
ATMi
0 4 6 8 10 0 4 6 8 10 CHX (Hours)
NICD
TUBULIN
140 kDa
95 kDa
50 kDa
C
Vehicle
ATMi
0 24 48 24 48 Carboplatin (Hours)
p-ATM
ATM
NICD
MDM2
TUBULIN
260 kDa
260 kDa
140 kDa
95 kDa
95 kDa
50 kDa
D
siNT
siMDM2
0 24 48 0 24 48 Carboplatin (Hours)
NICD
MDM2
TUBULIN
140 kDa
95 kDa
95 kDa
50 kDa
E
Start
Carboplatin 48h
Stop
4 2 1 0.5
CHX (Hours)
Carboplatin 48h
siNT
siMDM2
0 0.5 1 2 4 0 0.5 1 2 4 CHX (Hours)
NICD
MDM2
TUBULIN
140 kDa
95 kDa
95 kDa
50 kDa

 © The Author(s)

Figure EV2. NICD stabilization is ATM- and MDM2-dependent.

(**A**) A549 cells were incubated with 100 μM Carboplatin and with/without 10 μM KU-55933 (ATM inhibitor) or Ceralasertib (ATR inhibitor) for the indicated time followed by immunoblotting to detect the expression of the indicated proteins ($n = 2$). p, phosphorylated. (**B**) A549 cells were incubated with 100 μM Carboplatin for 48 h and with or without 10 μM KU-55933 (ATM inhibitor; ATMi). 50 μg/μl Cycloheximide (CHX) was added for the indicated time before the end of Carboplatin treatment ($n = 2$). Upper panel: Schematic representation of the experimental design. (**C**) Western blotting of the indicated proteins in A549 cells incubated with 100 μM Carboplatin for the indicated time, with or without 10 μM KU-55933 (ATM inhibitor; ATMi) ($n = 2$). (**D**) Western blotting of the indicated proteins in A549 cells transfected with a non-targeting *siRNA* (*siNT*) or with a *siRNA* against MDM2 (*siMDM2*) for 6 h, followed by addition of 100 μM Carboplatin for the indicated time ($n = 3$). (**E**) At 6 h post-transfection, A549 cells transfected with *siMDM2* or *siNT* were incubated with 100 μM Carboplatin for 48 h and with 50 μg/μl of Cycloheximide (CHX) for the indicated time before the end of Carboplatin treatment ($n = 2$). Upper panel: schematic representation of the experimental design. In the legend, *n* indicates the number of biological replicates. Source data are available online for this figure.

© The Author(s)

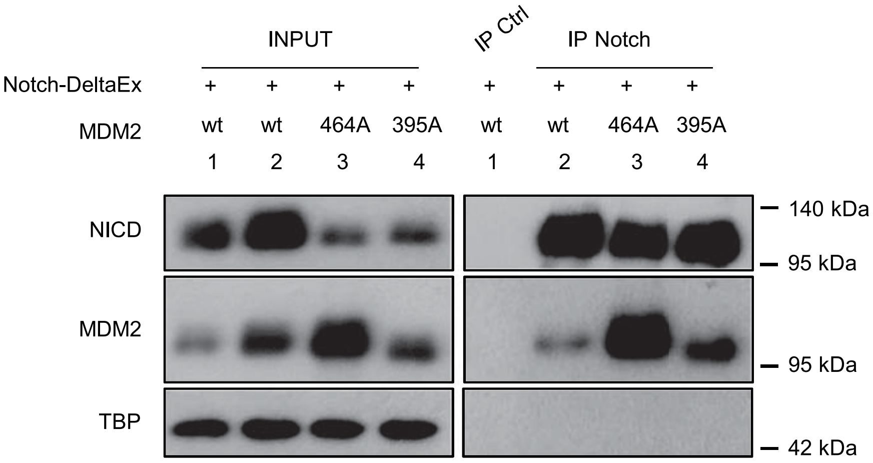

Figure EV3. NICD and MDM2 interaction.

293T cells were transfected with NOTCH1-DeltaEx, empty vector (pcDNA), MDM2 WT, MDM2 464 A or MDM2 395 A for 48 h. Chromatin extracts were used for NOTCH1 immunoprecipitation and the levels of the indicated proteins were measured by western blotting. INPUT: total cell extracts. IP ctrl: immunoprecipitation control with an unspecific mouse antibody, IP Notch: immunoprecipitation with an anti-NOTCH1 antibody. TBP was used as loading control in INPUT. Source data are available online for this figure.

 © The Author(s)

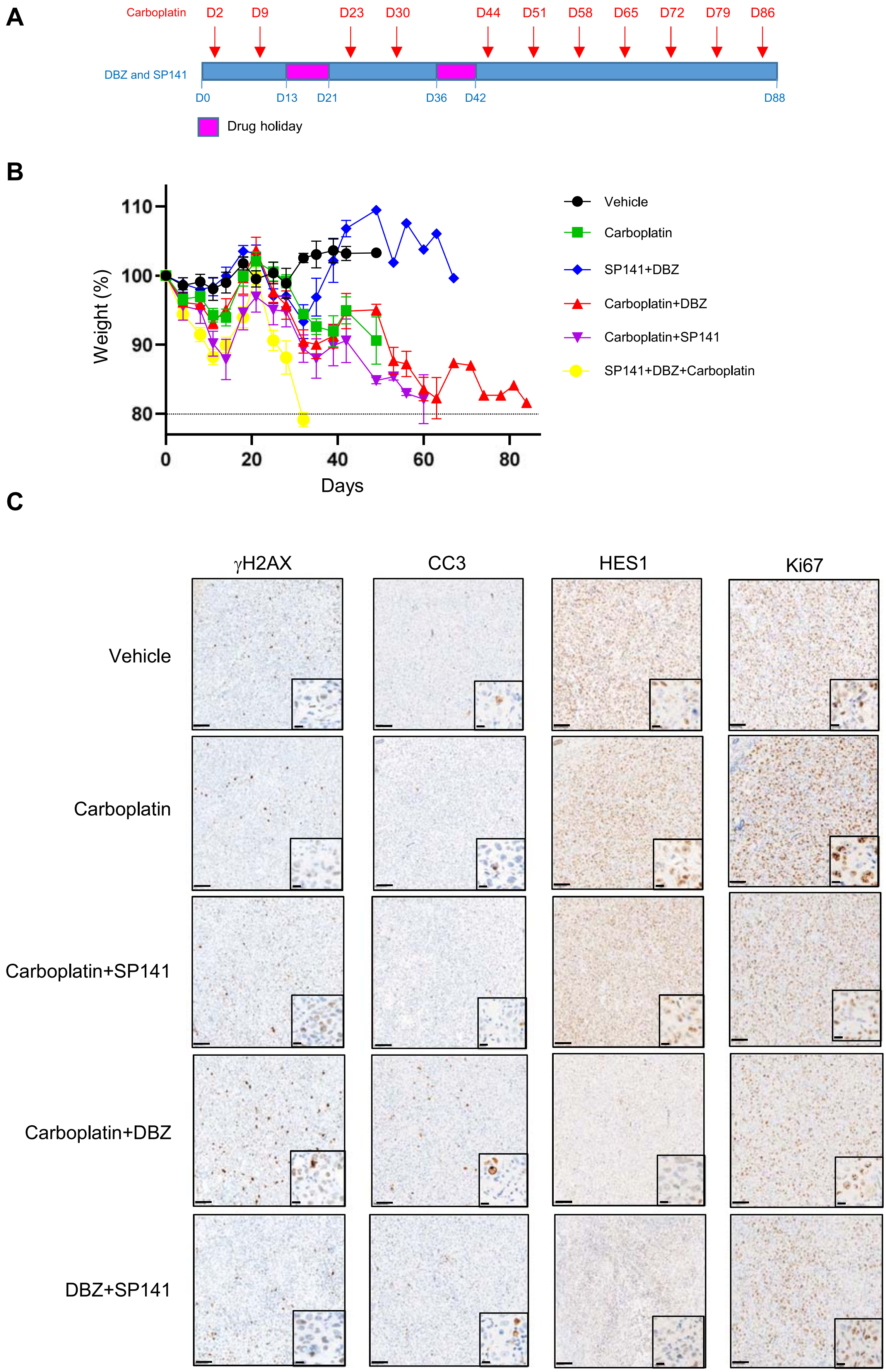
A
Carboplatin
D2
D9
D23
D30
D44
D51
D58
D65
D72
D79
D86
DBZ and SP141
D0
D13
D21
D36
D42
D88
Drug holiday
B
Weight (%)
110
100
90
80
0
20
40
60
80
Days
Vehicle
Carboplatin
SP141+DBZ
Carboplatin+DBZ
Carboplatin+SP141
SP141+DBZ+Carboplatin
C
γH2AX
CC3
HES1
Ki67
Vehicle
Carboplatin
Carboplatin+SP141
Carboplatin+DBZ
DBZ+SP141

Figure EV4. In vivo treatments.

(**A**) Schedule of the in vivo treatments. Carboplatin (50 mg/kg/day) was administered once per week (red arrow on top). SP141 (30 mg/kg/day) and DBZ (2.2 mg/kg/day) were administered 5 days per week. Pink color depicts the drug holidays. (**B**) Weight change curves in the different treatment groups as in Fig. 5B throughout the treatment period. Weights were normalized at each time point to day 0 and then represented as an average of all mice in each group. (**C**) Representative images of the IHC performed in PDX tumor samples from the indicated groups (data quantification in Figs. 5D and EV5A). Scale bars: 100 µm; Inset scale bars: 20 µm. Source data are available online for this figure.

 © The Author(s)

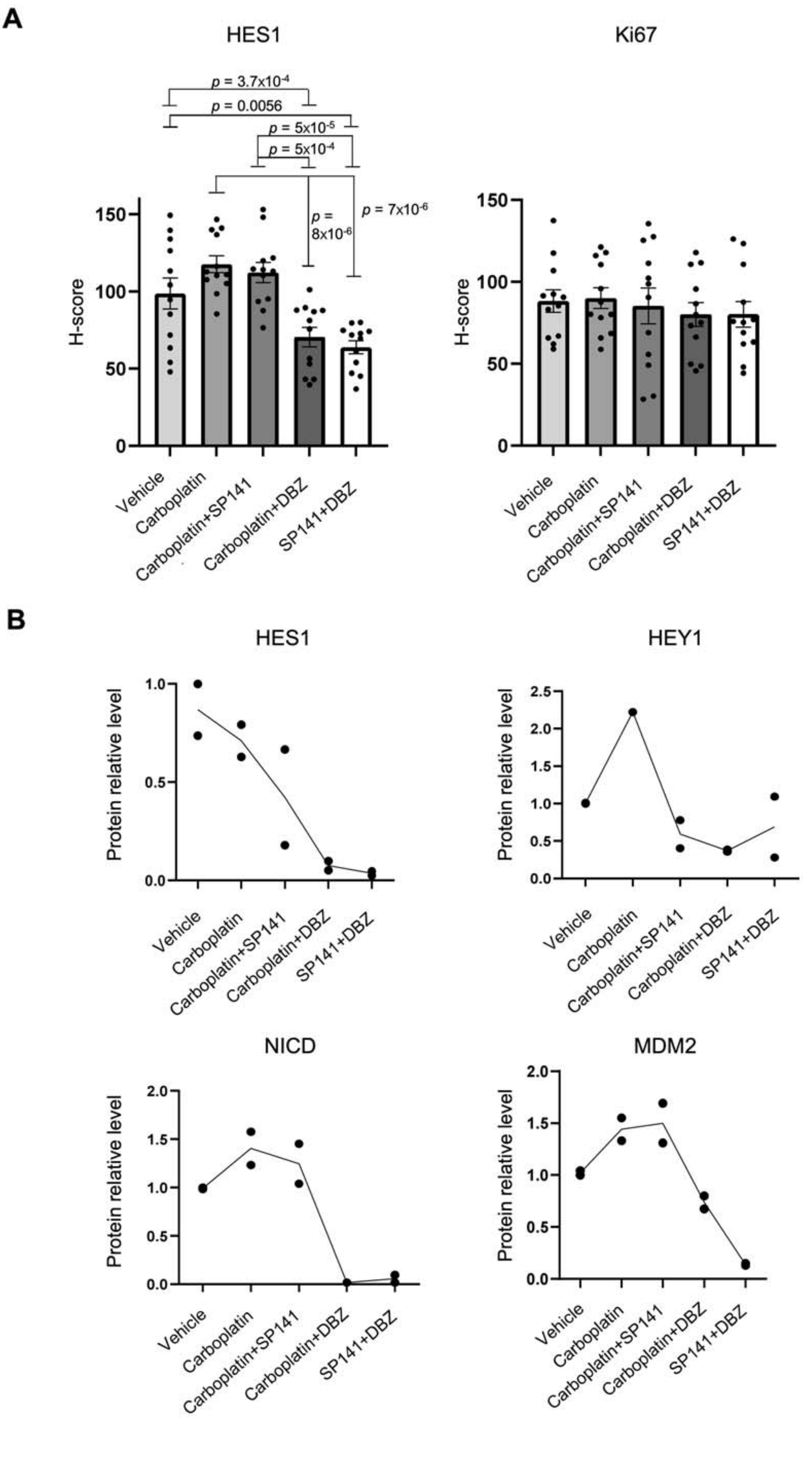
A
HES1
p = 3.7x10-4
p = 0.0056
p = 5x10-5
p = 5x10-4
p = 8x10-6
p = 7x10-6
H-score
Vehicle
Carboplatin
Carboplatin+SP141
Carboplatin+DBZ
SP141+DBZ
Ki67
B
HES1
HEY1
NICD
MDM2
Protein relative level

Figure EV5. IHC and western blot quantifications.

(**A**) IHC analysis of HES1 and Ki67 expression in PDX (TP57) xenografts from nude mice treated with vehicle, Carboplatin alone, Carboplatin + SP141, Carboplatin + DBZ, and SP141 + DBZ ($n = 12$ measures from 3 different tumors for all groups). Data are the mean ± SEM and they correspond to the analysis of 4 fields (original magnification, ×20) per tumour. *P* values were obtained by 1-way ANOVA followed by Tukey's multiple comparison test). (**B**) Quantification of the western blots in Fig. 5E. The level of each protein was normalized to GAPDH in the same condition. Then, protein signal in each lane was normalized to that in lane 1 (vehicle) set at 1. In the legend, *n* indicates the number of biological replicates. Source data are available online for this figure.

 © The Author(s)
